# Integrated NEK7-inflammasome and platelet transcriptomic signature generates mechanistic hypotheses in heart failure

**DOI:** 10.1371/journal.pone.0352124

**Published:** 2026-06-26

**Authors:** Zelan Wu, Zhisheng Zheng, Yongkang Li, Botao Li, Shiyu Pi, Daiqin Wu, Zhangrong Chen, Xia Li, Wei Li, Fangjie Dai

**Affiliations:** 1 Department of Cardiovascular Medicine, the Affiliated Hospital of Guizhou Medical University, Guiyang, Guizhou, China; 2 The Key Laboratory of Myocardial Remodeling Research, The Affiliated Hospital of Guizhou Medical University, Guiyang, Guizhou, China; 3 Guizhou Medical University, Guiyang, Guizhou, China; 4 Guizhou Precision Medicine Institute, the Affiliated Hospital of Guizhou Medical University, Guiyang, Guizhou, China; Augusta University, TAIWAN

## Abstract

**Background:**

The relationship between NEK7-NLRP3 inflammasome activation and elevated platelet activity in the progression of heart failure (HF) is not well understood. Additionally, a comprehensive gene network linking these processes to cardiac fibrosis has not been established. Our study aimed to explore the transcriptomic associations between NEK7-inflammasome, platelet activation and cardiac fibrosis in heart failure (HF), and to generate pathogenic hypotheses.

**Methods:**

We integrated bulk RNA-Seq (GSE116250, GSE57338) and scRNA-Seq (GSE183852) data from human HF samples. Through differential expression analysis, WGCNA, and machine learning techniques (LASSO and SVM-RFE), we identified key genes. A candidate diagnostic nomogram was subsequently developed and internally validated. We also conducted functional enrichment and immune infiltration analyses, and molecular docking studies. Key findings were explored in an angiotensin II-induced murine HF model using quantitative PCR.

**Results:**

We identified a novel four-gene signature, *TIMP2*, *COL16A1*, *MDK*, and *ISLR*, that shows co-expression patterns with *NEK7* and platelet‑activation‑related gene sets. These genes were significantly upregulated in human HF tissues and showed similar expression trends in murine hearts, although *ISLR* exhibited a non-significant increase. A preliminary discrimination model based on these genes showed preliminary discriminatory efficacy in the training cohort (AUC = 0.993), however, this model requires further validation in prospective, multi-center cohorts before any clinical application can be considered. Functional enrichment analysis indicated their synergistic involvement in the TGF-β signaling pathway. Immune profiling underscored a correlation with CD56dim natural killer cell infiltration, identifying fibroblasts as the primary expressors of these genes. Pseudotime trajectory analysis illustrated dynamic expression patterns during fibroblast activation, and potential therapeutic compounds, such as β-Heparin and WZ4002, were predicted via molecular docking.

**Conclusion:**

This study introduces a novel fibro-inflammatory gene module linked to NEK7-mediated inflammation, platelet activation, and TGF-β signaling in HF. These findings generate integrated mechanistic hypotheses for HF pathogenesis and provide a foundation for subsequent diagnostic and mechanistic explorations.

## Introduction

Heart failure (HF), affecting over 60 million people globally, is characterized by cardiac impairment that leads to dyspnea and limitations in physical activity [1]. It constitutes the final stage of various cardiac disorders, such as coronary artery disease, hypertensive heart disease, and cardiomyopathies [2]. These conditions are driven by myocardial remodeling, neurohormonal dysregulation, and inflammatory imbalances [3]. Currently, the diagnosis of HF primarily relies on echocardiography and the measurement of natriuretic peptides. Although echocardiography is accessible and cost-effective, it presents limitations in assessing the hemodynamic status [4]. Natriuretic peptides, on the other hand, are challenged by issues in diagnosing HFpEF, particularly by producing false negatives in obese patients [4]. More advanced techniques such as cardiac MRI and strain imaging provide benefits; however, they are limited by their cost and accessibility [5]. Emerging biomarkers, including galectin-3 and soluble ST2, show potential [6], yet they are hindered by standardization and cost issues. Consequently, exploring the transcriptomic associations underlying inflammation, thrombosis and fibrosis in HF to generate pathogenic hypotheses remains a critical and unmet need.

Recent research indicates that dysregulation of the immune microenvironment plays a significant role in the progression of HF, with macrophages orchestrating fibrotic responses and T lymphocytes contributing to myocardial hypertrophy [7]. Beyond individual cell types, the complex interactions between inflammatory signaling and other pathological processes, such as platelet activation and extracellular matrix (ECM) remodeling, are increasingly recognized as critical drivers of HF. Nonetheless, the key molecular networks capable of integrating these diverse pathways are still poorly defined.

NEK7, a core activator of the NLRP3 inflammasome, plays pivotal roles in chronic inflammation [8] and myocardial ischemia [9]. NEK7 potentially facilitates the binding to NLRP3’s LRR and NACHT domains, which may induce conformational shifts and oligomerization, and potentially promote the recruitment of ASC and pro-caspase-1 to form the NLRP3 inflammasome complex. The assembly dependent on NEK7 activates caspase-1, which processes pro-IL-1β and pro-IL-18 into bioactive cytokines and cleaves gasdermin D to form membrane pores, driving pyroptotic inflammation and myocardial injury [9]. In murine models of HF, activation of NLRP3 amplifies IL-1β secretion, thus accelerating cardiac remodeling [10]. Despite the critical role of NEK7 in NLRP3-mediated cardiac pathophysiology, its regulatory mechanisms and therapeutic potential remain incompletely characterized. Platelet activation also plays a significant role in HF pathogenesis through morphological changes, granule release, and aggregation [11]. Activated platelets release inflammatory mediators, such as P-selectin and CD40L [12,13], and pro-fibrotic factors, including TGF-β and platelet factor 4 [14,15], which contribute to microvascular dysfunction [16,17] and ECM remodeling [14,15]. Although the platelet-activating factor (PAF) contributes to NEK7-mediated inflammasome activation, the molecular bridge that potentially links NEK7-driven inflammation, platelet hyperactivity, and the resultant myocardial remodeling is yet to be fully elucidated.

We hypothesized that a cluster of genes might co-express with NEK7 and show transcriptional association with platelet activation pathways, potentially representing a crucial integrative link among inflammation, thrombosis, and fibrosis in HF. Identifying such a multi-gene signature could provide deeper insights into the pathogenesis of HF and offer diagnostic value superior to that of single molecules. This study utilized integrated bulk RNA-seq and scRNA-seq data to identify HF-associated gene signatures related to *NEK7* and platelet activation. Our objectives were to identify a potential transcriptomic signature co-expressed with NEK7 and platelet activation, to analyze their functional correlations and immune interactions, and to generate integrated mechanistic hypotheses for HF pathogenesis. Single-cell analysis was employed to map expression patterns and intercellular communication networks. Additionally, transcription factor-miRNA analysis predicted regulatory mechanisms, and molecular docking identified potential therapeutics. Collectively, these approaches provided new insights into the pathogenesis of HF and paved the way for the development of multi-target diagnostic and therapeutic strategies.

## Materials and methods

### Source of data

The Gene Expression Omnibus (GEO) database (http://www.ncbi.nlm.nih.gov/geo/) was utilized to obtain datasets related to HF. The GSE116250 dataset on the GPL16791 platform consisted of samples from 50 left ventricular tissues and 14 control samples [18]. The GSE57338 dataset (GPL11532) was used as the validation set, containing 177 samples of left ventricular tissue and 136 control samples [19]. the GSE5406 dataset (GPL96) was also used as the validation set, consisting of 194 samples of left ventricular tissue and 16 control samples [20]. Additionally, the GSE183852 dataset, based on the GPL24676 sequencing platform, comprised 5 transmural left ventricular apical tissue samples and 2 control samples [21]. To validate the comparability of GSE5406 with the model-development dataset (GSE116250) and initial validation dataset (GSE57338), we extracted baseline characteristics including species, tissue source, sample grouping, data type, sequencing platform, and sample size (Table 1). All three datasets were derived from human left ventricular tissues, with consistent grouping of heart failure (HF) and non-failing (NF) controls. GSE57338 and GSE5406 were microarray datasets normalized using the Robust Multi-Array Analysis (RMA) algorithm, while GSE116250 was a high-throughput sequencing dataset standardized uniformly. Batch effects were evaluated using the sva package in R, and no significant batch bias was observed after standardization, ensuring the three datasets were comparable and suitable for external validation [22].

**Table 1:** Standardization of the three datasets.

GSE number	Platform	Sequencing type	Tissue	Country	Group
GSE116250	GPL16791	High-throughput sequencing	Left ventricular	USA	37 DCM, 13 ICM, 14 NF
GSE57338	GPL11532	Microarray	Left ventricular	USA	82 IDCM, 95 ICM, 136 NF
GSE5406	GPL96	Microarray	Left ventricular	USA	86 IDCM, 108 ICM, 16 NF

**Abbreviations:** DCM, Dilated Cardiomyopathy; IDCM, Idiopathic Dilated Cardiomyopathy; ICM, Ischemic Cardiomyopathy; NF, Non-failing.

The platelet activation-related pathway (REACTOME_PLATELET_ACTIVATION_SIGNALING_AND_AGGREGATION) was accessed by searching for the keyword “Platelet” in the Molecular Signatures Database (MSigDB) (https://www.gsea-msigdb.org/gsea/msigdb). The 261 genes within this pathway were designated as platelet activation-related genes (PCRGs).

### Differential gene expression identification

The “DESeq2” package (v 1.38.0) [23] was employed to perform differential expression analysis on the GSE116250 dataset, with the aim of identifying differentially expressed genes (DEGs). Genes were filtered based on thresholds of *P.*adj < 0.05 and |log_2_Fold Change (FC)| > 1. Volcano and heatmap plots were generated using the “ggplot2” (v 3.4.1) [24] and “ComplexHeatmap” (v 2.14.0) [25] packages, respectively. The top 10 upregulated and downregulated DEGs, selected based on their |log_2_FC| values, were highlighted in both the volcano plot and the heatmap.

### Weighted gene co-expression network analysis (WGCNA)

To identify gene modules co-expressed with *NEK7* and platelet activation, we first intersected PCRGs with DEGs to pinpoint those PCRGs that were significantly differentially expressed between the HF group and the control group.

The “GSVA” package (v 1.46.0) [23] was then used to calculate the enrichment scores of these differentially expressed PCRGs. A Wilcoxon test confirmed their differential expression between the two groups (*P* < 0.05). A similar analysis was performed for *NEK7* expression.

Based on these findings, the enrichment scores of differentially expressed PCRGs and *NEK7* expression levels were used as phenotypes to construct a co-expression network using the “WGCNA” package (v 1.71) [26]. The method of constructing a signed network was adopted. During the calculation of adjacency relationships, only positive correlations (r > 0) were retained. By excluding negative correlations, this approach minimizes the interference from irrelevant reverse expression relationships related to functions and focuses the analysis on the synergistic regulatory modules related to disease progression. Based on the hclust function, we performed sample clustering to eliminate outliers and ensure data quality. In the signed network setting, the pickSoftThreshold function was used to select the optimal soft threshold power (β) within the range of 1–20. Dynamic tree cutting was used to identify co-expression modules (minimum size of 100 genes), merging those with a similarity greater than 0.4. The module that showed the strongest positive correlation with both PCRG scores and *NEK7* expression was selected as the key module. Further analysis identified genes in this module that met dual thresholds (|MM| > 0.8, |GS| > 0.2) as key module genes [26].

### Acquisition of candidate genes and their functional analysis

The “ggvenn” package (v 1.7.3) [27] was employed to intersect DEGs with key module genes to identify candidate genes. To explore the related biological functions and pathways, Gene Ontology (GO) and Kyoto Encyclopedia of Genes and Genomes (KEGG) enrichment analyses were conducted on the candidate genes using the “clusterProfiler” package (v 4.2.2) [28], with significance set at *P* < 0.05. The GO analysis included categories such as cellular component (CC), molecular function (MF), and biological process (BP). Subsequently, these candidate genes were uploaded to the Search Tool for the Retrieval of Interacting Genes/Proteins (STRING) database (http://www.string-db.org/), employing an interaction score threshold of>0.15 to construct the protein-protein interaction (PPI) network.

### Recognition of key genes

To identify key genes in the GSE116250 dataset, this study applied two machine learning algorithms to conduct feature selection and dimensionality reduction on previously selected candidate genes. First, the Least Absolute Shrinkage and Selection Operator (LASSO) regression analysis was utilized, employing the “glmnet” software package (v 4.1.4) (https://www.jstatsoft.org/v33/i01). A logistic regression model was constructed, and 10-fold cross-validation was used to determine the optimal penalty coefficient (lambda). The cross-validation aimed to minimize the deviation, resulting in the selection of lambda = 0.000160471. This model retained 10 feature genes with non-zero regression coefficients, effectively selecting features and controlling multicollinearity [29]. Second, the Support Vector Machine Recursive Feature Elimination (SVM-RFE) algorithm was concurrently used, executed through the “e1071” software package (v 4.7–1.1) [30]. This method also assessed the model performance using 10-fold cross-validation. By recursively eliminating features, it identified a combination of 5 feature genes corresponding to the lowest average error rate. The common candidate key genes selected by these algorithms were identified as the intersection and visualized using the “ggvenn” software package (v 1.7.3). To identify differentially expressed candidate biomarkers between the HF and control groups, Wilcoxon tests (*P* < 0.05) were performed on datasets GSE116250 and GSE57338.

### Nomogram construction and evaluation

To mitigate overfitting, logistic regression with L2 regularization (ridge regression, penalty = 1) was performed using the “rms” package (v 6.5−0) [31] to construct the nomogram, with the maximum iteration number (maxit) set to 1000 to ensure convergence. The formula for calculating the line graph is: logit(P)=β₀ + β_1_ × TIMP2 + β_2_ × COL16A1+β_3_ × MDK + β_4_ × ISLR, where P is the probability of HF, β_0_ is the intercept, and β_1_-β_4_ are the regression coefficients. For model stability assessment, 10 iterations of 10-fold repeated cross-validation were performed on the training set (GSE116250) using the “glmnet” package to calculate the mean AUC, sensitivity, and specificity. Bootstrap optimism correction with 1000 resamplings was conducted using the “rms” package to calculate the corrected AUC and calibration curves for the training and validation sets, correcting for overfitting bias. For cross-dataset calibration assessment, integrated calibration curves and stratified calibration curves were plotted by combining GSE116250, GSE57338, and GSE5406 datasets.To evaluate the discrimination of the nomogram, the “pROC” package (v 1.18.0) [32] was used to generate a Receiver Operating Characteristic (ROC) curve, and the area under the curve (AUC) was calculated to evaluate the nomogram’s predictive value (an AUC > 0.7, approaching 1, indicates higher diagnostic accuracy). Calibration curves were plotted, and Hosmer-Lemeshow test was performed (*P* > 0.05 indicates good calibration). For external validation sets (GSE57338, GSE5406), logistic calibration was used to correct for systematic bias in gene expression scales before plotting calibration curves. Decision curve analysis (DCA) was performed using the “rmda” package (v 1.6) [33] to evaluate the clinical net benefit of the nomogram. Furthermore, the expression levels of the biomarkers were verified in the additional external validation dataset GSE5406.

### Analysis of the basic characteristics of key genes

To investigate the expression specificity of key genes, *NEK7* and these genes were analyzed using the Human Protein Atlas (HPA) database (https://www.proteinatlas.org/) to display their expression across various tissues. Additionally, the chromosomal distribution of *NEK7* and the key genes was visualized using the “RCircos” package (v 1.2.2) [34]. To determine their subcellular localization, *NEK7* and the key genes were examined in the Universal Protein Resource (UniProt) database (https://www.uniprot.org/), which provided information on their subcellular distribution. Concurrently, Spearman correlation analysis (|correlation (cor)| > 0.3 and *P <* 0.05) was performed using the “psych” package (v 2.2.9) [35] to assess the correlations among *NEK7* and key genes in both HF and control groups of the GSE116250 study.

### Gene set enrichment analysis (GSEA)

GSEA was conducted via the “ClusterProfiler” package (v 4.2.2) to explore the biological functions of biomarkers in HF development. Spearman correlations between *NEK7*, key genes, and all genes in GSE116250 were calculated using the “psych” package (v 2.2.9). Genes were ranked by correlation coefficients to generate lists for *NEK7* and each key gene, followed by GSEA with the “c2.cp.kegg_legacy.v2024.1.Hs.symbols” reference set from “org.Hs.e.g.,db” (v 3.16.0) [36] (*P*.adj < 0.05, |Normalized Enrichment Score (NES)| > 1). The top five signaling pathways, sorted in descending order according to *P*.adj value, of the *NEK7* and key genes enrichment results were visualized.

### Immune cell infiltration analysis

Immune cell infiltration analysis in GSE116250 examined differences in the immune microenvironment between the HF and control groups. Infiltration scores for 28 immune cells [37] were computed using the “GSVA” package (v 1.46.0). After filtering samples by confidence, Wilcoxon rank‑sum tests were performed to compare infiltration levels between the two groups. To control the false discovery rate (FDR) under multiple testing, the raw P values were adjusted using the Benjamini‑Hochberg (BH) procedure, and differential immune cells (DICs) were defined as those with adjusted *P*  .adj < 0.05. Spearman correlations were employed via the “psych” package (v 2.2.9) to explore associations among DICs and between *NEK7*/key genes and DICs (|cor| > 0.3, *P* < 0.05).

### Construction of molecular regulatory networks

To investigate the regulatory mechanisms of *NEK7* and key genes, their transcription factors (TFs) were predicted using the JASPAR database in NetworkAnalyst. miRNAs interacting with them were predicted from TarBase v9.0 (NetworkAnalyst) and miRDB, with the intersection of the results from these two databases considered.

### Drug prediction and molecular docking

We queried the DSigDB database through “enrichR” [38] to predict candidate drugs/compounds interacting with *NEK7* and key genes (*P <* 0.05), thereby identifying potential HF-targeting drugs. For each candidate, we selected the drug with the highest binding score and lowest *P*-value for molecular docking. The 3D structures of target proteins were downloaded from the RCSB PDB, and ligand structures were obtained from PubChem. Molecular docking was conducted using the CB-Dock2 online server, and binding affinity was evaluated as the total score (absolute Vina score). A total score > 4.0 kcal/mol, > 5.0 kcal/mol, and >7.0 kcal/mol indicated moderate, good, and strong binding activity, respectively.

### scRNA-seq data analysis

scRNA-seq data from GSE183852 were analyzed using the “Seurat” package [39]. Initial filtering excluded cells with fewer than 200 detected genes and genes present in fewer than 3 cells; retained cells/genes met the following criteria: 1,000–35,000 features per cell, fewer than 10,000 total reads per cell, and less than 10% mitochondrial reads. The filtered data were normalized using the LogNormalize method with a scale factor of 10,000 via the “NormalizeData” function, and the top 2,000 highly variable genes were identified via “FindVariableFeatures”. PCA was used to analyze their distribution in the HF and control groups, with “JackStraw” computing PC gene P-values to select significant PCs (*P* < 0.05). Unsupervised clustering (resolution = 0.4) via “FindNeighbors” and “FindClusters” was visualized by UMAP. Cell types were annotated using literature-derived marker genes [40], and marker gene expression in clusters was analyzed.

### Acquisition of key cells

To screen for genes related to key cells, infiltration proportions of cell types in GSE183852 HF and control groups were visualized via “ggplot2” (v 3.4.1). *NEK7* and key gene expression across cell types were analyzed, with Wilcoxon tests identifying differential expression between groups within each cell type (*P* < 0.05). Key cell populations were selected for high abundance in both groups, detectable NEK7/key gene expression, and significant group expression differences.

### Pseudo-time and functional enrichment analysis

Key cell populations underwent secondary clustering using the aforementioned approach. In GSE183852, the Monocle algorithm analyzed their pseudo-time, visualizing *NEK7* and key gene expression patterns across developmental stages. “FindAllMarkers” identified genes influencing differentiation in HF samples (retaining positive markers with min.pct = 0.25, logfc.threshold = 0.25). Critical cells were subgrouped by these markers, with a heatmap displaying the top 30 DEGs. ClusterProfiler performed functional enrichment (GO: BP, CC, MF; KEGG) on differentiation-related genes (*P*.adj < 0.05).

### Cell communication and TF regulatory analysis

To analyze interactions among critical cell populations in GSE183852 for both HF and control groups, the CellChat package (v 1.6.1) [41] was utilized to construct cell communication networks, focusing on critical cell interactions to generate maps. Additionally, the viper package (v 1.32.0) [42] analyzed regulatory relationships within critical cells, inferring TF activities across states and their downstream regulatory impacts.

### Animal model of HF

To investigate the functional roles of key genes identified from public databases, HF was induced in male C57BL/6 mice (8–10 weeks old) provided by Cyagen Biosciences Inc., Suzhou, China, via continuous subcutaneous infusion of angiotensin II (Ang II). All procedures were approved by the Animal Ethics Committee of Guizhou Medical University (Approval No. 2200536; Date: March 3, 2022). The experiments adhered strictly to institutional guidelines for the humane care and use of laboratory animals.

### Animal husbandry and welfare

All mice were maintained under specific pathogen-free (SPF) conditions in the barrier facility of the Laboratory Animal Center at Guizhou Medical University. Environmental parameters were strictly controlled, including temperature (22 ± 1°C), humidity (50 ± 10%), and a 12-hour light/dark cycle. Mice were group-housed, with three to four animals per cage, and had ad libitum access to a standard breeding diet and water. Bedding was replaced twice weekly. All personnel involved in the study received standardized training in laboratory rodent handling, procedural techniques, and postoperative care.

### Humane endpoints and health monitoring

Humane endpoint criteria were established in accordance with the guidelines from NC3Rs. To implement these endpoints, animal health was systematically assessed through daily observations of general appearance (posture, coat condition), activity levels, and behavior, in addition to weekly body weight measurements. Immediate euthanasia was mandated if any of the following predefined criteria were met: (1) a body weight loss exceeding 20% of baseline; (2) severe lethargy, prostration, or unresponsiveness; (3) overt signs of severe distress, such as labored breathing or hunched posture; or (4) any unforeseen severe clinical event, such as seizures or paralysis. No animals died prior to meeting these endpoint criteria.

### Model induction and surgical procedure

Mice were randomly assigned to either the Ang II infusion group (or the normal control (NC) group. Angiotensin II (Ang II, Macklin, catalog #A920840) was dissolved in sterile saline to deliver 1.5 mg/kg/day via osmotic minipumps (Alzet Model 2004, catalog #2004) [43]. All mice underwent subcutaneous implantation of a minipump for continuous delivery over four weeks. Pumps in the Ang II group contained the Ang II solution, while those in the NC group contained only saline.

Surgery was performed under aseptic conditions. Anesthesia was induced and maintained with inhaled isoflurane (4% for induction, 1.5–2% for maintenance). A small interscapular incision was made to create a subcutaneous pocket where a primed minipump was inserted. The incision was closed with absorbable sutures. Postoperative analgesia was administered via subcutaneous injection of buprenorphine (every 12 hours) for at least 48 hours.

At the experimental endpoint, four weeks post-implantation, cardiac function was evaluated by echocardiography. Following assessment, animals were euthanized by cervical dislocation under deep anesthesia (5% isoflurane), with death confirmed by the absence of pedal and corneal reflexes.

### Echocardiography and heart morphology

Transthoracic echocardiography was performed on isoflurane-anesthetized (0.5–1%) mice prior to sacrifice using a SiliconWave 60 system equipped with an L38-22K3 transducer (30 MHz). M-mode images were analyzed by a blinded operator to determine functional and structural parameters, including left ventricular ejection fraction (LVEF), left ventricular fractional shortening (LVFS), left ventricular end-diastolic diameter (LVEDD), left ventricular end-systolic diameter (LVESD), diastolic and systolic left ventricular posterior wall thickness (LVPWTd, LVPWTs), and diastolic and systolic interventricular septal thickness (IVSd, IVSs). Following sacrifice, hearts were excised, photographed, and weighed to calculate the heart weight to body weight (HW/BW) ratio.

### Reverse transcription quantitative polymerase chain reaction (RT-qPCR)

Total RNA was extracted from heart tissues utilizing TRIzol reagent (Vazyme, catalog #R401-01). Reverse transcription was conducted using the HP All-in-one qRT Master Mix II RT203-Ver.1 kit (Kunming Yungeng Biotechnology, catalog #24Y0124). Quantitative PCR was performed, with normalization to GAPDH across three biological replicates per group and three technical replicates per sample. Relative gene expression levels were determined using the 2 ⁻ ^ΔΔCt^ method (*P* < 0.05) [44]. This section represents a preliminary mRNA expression detection with a limited animal sample size (n = 3 per group).

### Statistical analysis

Bioinformatic analyses were performed using R software (v4.2.2). Statistical significance was established at *P* < 0.05. Data from animal experiments were analyzed through GraphPad Prism 10.4.2, employing unpaired t-tests or Wilcoxon rank-sum tests as appropriate. The results are expressed as the mean ± SEM, with statistical significance set at *P* < 0.05.

## Results

### Acquisition of candidate genes

**Fig 1:**
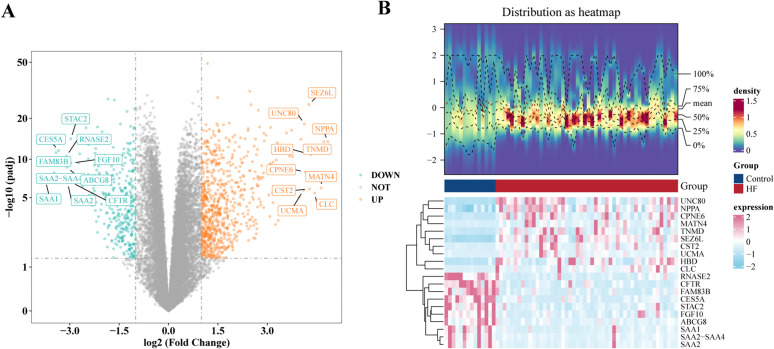
Differential Expression Analysis of Genes in HF versus Control Groups. (A) Volcano plot showing DEGs between the HF and control groups. The x-axis represents log_2_ FC, and the y-axis shows -log10(*P*.adj). Significant up-regulated genes are highlighted in orange, down-regulated genes in cyan, and non-significant genes in gray. (B) Heatmap of top 10 up- and down-regulated genes based on |log_2_FC|. The heatmap presents relative expression levels using a color scale, while the density distribution above the heatmap indicates the percentage of gene expression within each group.

In the dataset GSE116250, 1,073 DEGs were identified between the HF and control groups. Among these, 744 genes were up-regulated and 329 were down-regulated (*P.*adj < 0.05, |log_2_FC| > 1) (Fig 1A,B, S1 Table).

### Acquisition of key module genes

**Fig 2:**
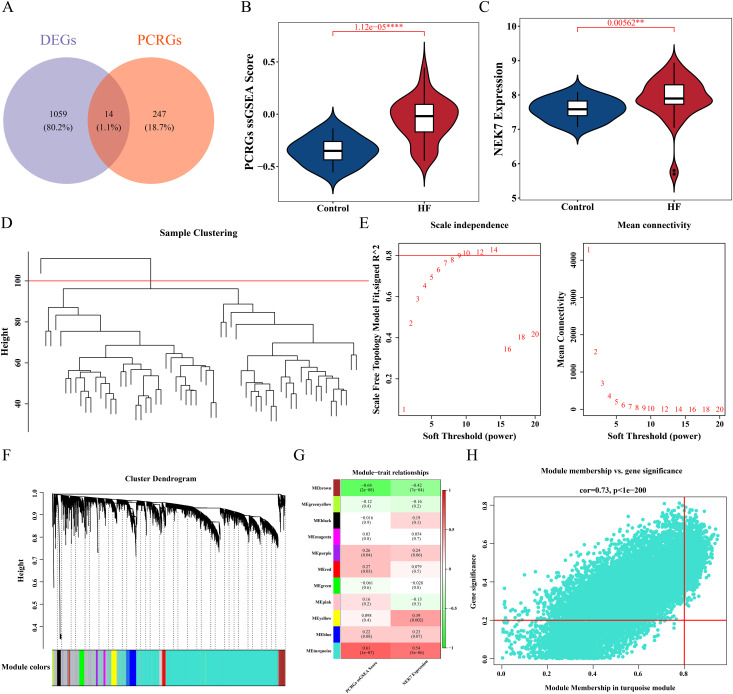
Identification and Analysis of DEGs and PCRGs in HF. (A) Venn diagram showing the intersection between DEGs and PCRGs, identifying 14 differentially expressed PCRGs. (B) Comparison of ssGSEA scores for the 14 PCRGs, revealing significant differences between the HF and control groups (P = 0.00001). (C) *NEK7* expression levels were significantly higher in HF compared to controls (*P* = 0.00562), suggesting its association with HF. (D) Sample clustering dendrogram after removing an outlier from the training set. (E) Determination of the optimal soft threshold (β = 10) for WGCNA analysis based on R² and mean connectivity. (F) Gene module cluster dendrogram merged at a height of 0.4, resulting in 11 modules. (G) Heatmap highlighting the turquoise module as key due to its highest correlation with ssGSEA scores of PCRGs and NEK7 expression. (H) Scatter plot selecting 1,121 key module genes based on |MM| > 0.8 and |GS| > 0.2 criteria.

DEGs were intersected with 261 PCRGs (S2 Table), identifying 14 differential PCRGs (*APOA1, ISLR, LEFTY2, GNB3, F2R, TGFB2, SERPINA3, F2RL2, KNG1, GP5, F5, LCK, F2RL3, GNG4*) (Fig 2A). In the HF versus control groups, ssGSEA scores of these 14 PCRGs (*P* = 0.00001) (Fig 2B, S3 Table) and NEK7 expression (*P* = 0.00562) were higher in HF (Fig 2C). In the training set, one outlier was removed before performing WGCNA (Fig 2D). The optimal soft threshold (β = 10) was determined when R² = 0.8, approximating a scale-free distribution (Fig 2E). Merging modules at height 0.4 yielded 11 modules (excluding gray) (Fig 2F). The turquoise module, which showed the highest correlation with both the ssGSEA scores of the 14 PCRGs and *NEK7* expression, was identified as key (Fig 2G). Filtering by |MM| > 0.8 and |GS| > 0.2 identified 1,121 key module genes (Fig 2H, S4 Table).

### The functions and pathways of candidate genes

**Fig 3:**
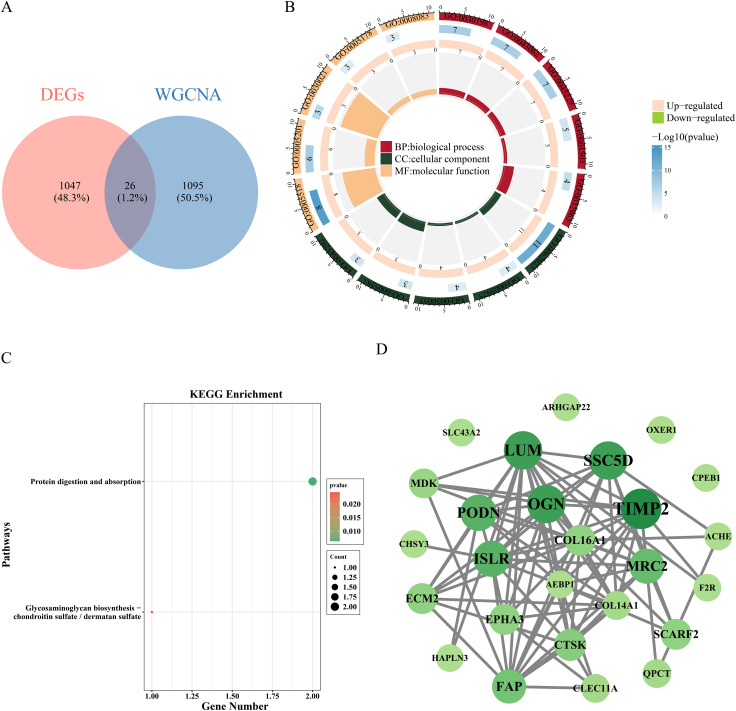
Functional and Pathway Analysis of Candidate Genes in HF. (A) Venn diagram depicting the intersection between DEGs and key module genes from WGCNA, identifying 26 candidate genes. (B) Circular plot illustrating GO enrichment analysis of the 26 candidate genes, with significant enrichment in 237 GO terms (BPs, CCs, MFs). (C) KEGG pathway analysis indicating notable enrichment in protein digestion/absorption and glycosaminoglycan biosynthesis pathways. (D) PPI network analysis highlighting LUM, SSC5D, TIMP2, OGN, PODN, MRC2, and ISLR as potential key regulatory factors due to high interaction scores.

DEGs were intersected with key module genes, resulting in the identification of 26 candidate genes (*MDK, ISLR, TIMP2, COL16A1, SSC5D, PODN, COL14A1, LUM, OXER1, SCARF2, SLC43A2, F2R, QPCT, AEBP1, ACHE, ARHGAP22, OGN, MRC2, CTSK, CLEC11A, CPEB1, HAPLN3, ECM2, EPHA3, CHSY3*, and *FAP*) (Fig 3A).

Functional enrichment analysis characterized the biological functions and pathways of these candidate genes. They were significantly enriched in 237 GO entries (173 BPs, 27 CCs, 37 MFs; *P* < 0.05), including collagen metabolic process, extracellular structure organization, and ECM structural constituent for compression resistance (Fig 3B, S5 Table). Additionally, two KEGG pathways were enriched: protein digestion and absorption, and glycosaminoglycan biosynthesis, chondroitin sulfate/dermatan sulfate (*P* < 0.05) (Fig 3C, S6 Table). PPI analysis identified LUM, SSC5D, TIMP2, OGN, PODN, MRC2, and ISLR as key network regulators with more interactions (score>0.15) (Fig 3D).

### Screened key genes: *TIMP2, COL16A1, MDK* and *ISLR*

**Fig 4:**
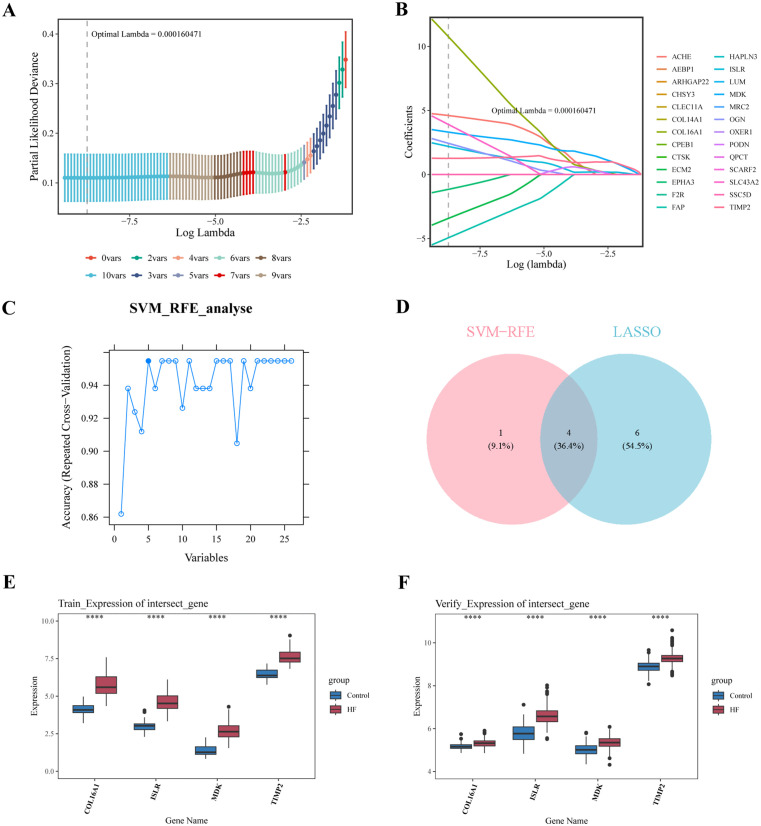
Identification of Key Genes in HF. (A-B) LASSO regression analysis identifying 10 characteristic genes with regression coefficients not penalized to zero at a lambda value of 0.000160471. (C) SVM-RFE analysis determining the highest model prediction accuracy with five characteristic genes: *TIMP2*, *COL16A1*, *MDK*, *ISLR*, and *PODN*. (D) Overlapping the characteristic genes from LASSO and SVM-RFE analyses resulted in four candidate key genes: *TIMP2*, *COL16A1*, *MDK*, and *ISLR*. (E-F) Expression analysis confirmed that these four candidate key genes were significantly up-regulated in both datasets compared to control groups, identifying them as key genes (*P* < 0.05).

LASSO regression identified 10 characteristic genes (*MDK, ISLR, TIMP2, COL16A1, SLC43A2, ACHE, OGN, ECM2, EPHA3, FAP*) with non-zero coefficients at a lambda value of 0.000160471 (Fig 4A, B). SVM-RFE analysis achieved the highest prediction accuracy using five genes: *TIMP2, COL16A1, MDK, ISLR, PODN* (Fig 4C). The overlap of these analyses yielded four candidate key genes: T*IMP2, COL16A1, MDK, ISLR* (Fig 4D).

Expression analysis demonstrated that these four genes were significantly up-regulated in the HF versus control groups across datasets with consistent trends. Thus, these genes were identified as key genes (*P* < 0.05) (Fig 4E, F).

### Candidate nomogram predictive performance validation and reliability assessment

**Fig 5:**
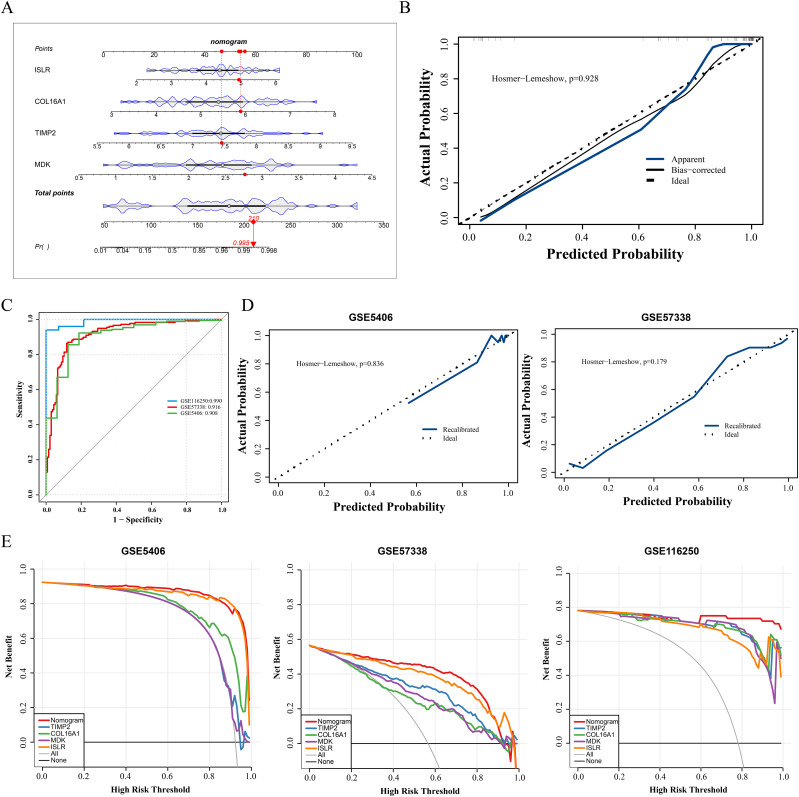
Development and comprehensive validation of the four-gene signature-based candidate diagnostic nomogram for heart failure. (A) Nomogram constructed using L2 regularization (ridge regression) to prevent overfitting, incorporating *TIMP2*, *COL16A1*, *MDK*, and *ISLR* for HF risk prediction. (B) Calibration curve of the nomogram in the training set (GSE116250) after 1000-times bootstrap optimism correction; Hosmer–Lemeshow test *P* = 0.928 indicates excellent calibration. (C) Combined receiver operating characteristic (ROC) curves of the nomogram in the training set (GSE116250, AUC = 0.990), initial validation set (GSE57338, AUC = 0.916), and additional external validation set (GSE5406, AUC = 0.908). (D) Calibration curves of the nomogram in two external validation sets (GSE57338 and GSE5406) after logistic calibration to correct systematic expression bias; Hosmer–Lemeshow test *P* = 0.179 for GSE57338 and *P* = 0.836 for GSE5406. (E) Decision curve analysis (DCA) of the nomogram and single genes in GSE116250, GSE57338, and GSE5406, showing superior net benefit of the nomogram across most high-risk thresholds. The model was validated by 10 iterations of 10-fold repeated cross-validation, 1000-times bootstrap optimism correction, and cross-dataset calibration to ensure stability and generalizability.

First, the comparability of the three datasets was verified: baseline characteristics including species, tissue source, sample grouping and sequencing platform were highly consistent, and batch effect examination using the sva package confirmed no significant bias after unified standardization (Table 1). The candidate diagnostic nomogram was constructed incorporating L2 regularization (ridge regression) to effectively prevent overfitting (Fig 5A). 10 iterations of 10‑fold repeated cross‑validation on the training set GSE116250 yielded a mean AUC of 0.989, mean sensitivity of 0.972 and mean specificity of 0.965, verifying robust model stability.Bootstrap optimism correction with 1000 resamplings generated a corrected AUC of 0.985 for the training set; the calibration curves closely fitted the ideal diagonal, and the Hosmer–Lemeshow test yielded *P* = 0.928, indicating excellent calibration (Fig 5B). ROC analysis showed the nomogram achieved an AUC of 0.990 (95% CI: 0.980–1.000) in the training set GSE116250, 0.916 in the initial validation set GSE57338, and 0.908 in the additional external validation set GSE5406, with all values exceeding 0.9 and demonstrating superior discriminative ability across datasets (Fig 5C). Integrated cross‑dataset calibration confirmed that predicted probabilities were highly consistent with actual observed probabilities without significant deviation. For the two external validation sets, logistic calibration corrected systematic gene‑expression scaling bias; the recalibrated curves closely approximated the ideal line, with Hosmer–Lemeshow *P* = 0.179 for GSE57338 and *P* = 0.836 for GSE5406 (Fig 5D). Decision curve analysis (DCA) across GSE116250, GSE57338 and GSE5406 showed that the nomogram provided a significantly higher net benefit than any single‑gene model across most high‑risk threshold intervals (Fig 5E), supporting its reliable predictive performance in bioinformatic analysis. The detailed regression coefficients of the nomogram model are listed in Table 2.

**Table 2:** Nomogram model coefficients.

	coefficients	S.E.	Wald Z	Pr（>|Z|）
Intercept	−153.3800	125.8659	−1.22	0.2230
*TIMP2*	19.1478	16.7436	1.14	0.2528
*COL16A1*	1.4294	3.1712	0.45	0.6522
*MDK*	13.3594	11.3635	1.18	0.2397
*ISLR*	−3.1256	4.8941	−0.64	0.5231

**Note:** The model was constructed with L2 regularization and validated via bootstrap optimism correction and cross-dataset calibration to ensure stability.

### Exploration of basic properties in key genes

**Fig 6:**
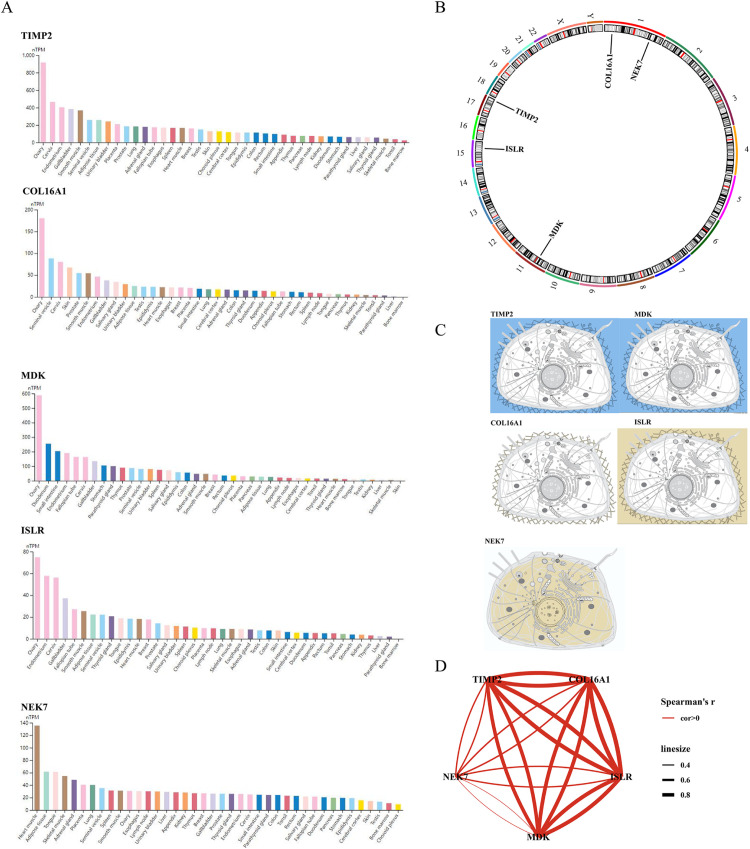
Exploration of Basic Properties in Key Genes. (A) Expression levels of key genes (*NEK7*, *TIMP2*, *COL16A1*, *MDK*, and *ISLR*) in myocardial tissue. (B) Chromosomal localization of the key genes: *NEK7* and *COL16A1* on chromosome 1, *MDK* on chromosome 11, *ISLR* on chromosome 15, and *TIMP2* on chromosome 17. (C) Subcellular localization of the key genes: *TIMP2*, *MDK*, and *ISLR* localized outside the cell membrane, *NEK7* primarily in the cytoplasm, and *COL16A1* in the ECM. (D) Correlation analysis indicating positive correlations among all key genes, with the highest correlation between *COL16A1* and *ISLR* (cor = 0.92, *P* < 0.001).

*NEK7* exhibited the highest expression in myocardial tissue (Fig 6A). Chromosomal localization identified *NEK7* and *COL16A1* on chromosome 1, *MDK* on chromosome 11, *ISLR* on chromosome 15, and *TIMP2* on chromosome 17 (Fig 6B). Subcellular localization findings were as follows: *TIMP2, MDK,* and *ISLR* were located outside the cell membrane; *NEK7* was found in the cytoplasm; *COL16A1* was located in the ECM (Fig 6C). Correlation analysis (|cor| > 0.3, *P* < 0.05) revealed positive correlations among all genes, with *COL16A1* and ISLR showing the strongest correlation (cor = 0.92, *P* < 0.001) (Fig 6D).

### Key genes shared enriched signaling pathways

**Fig 7:**
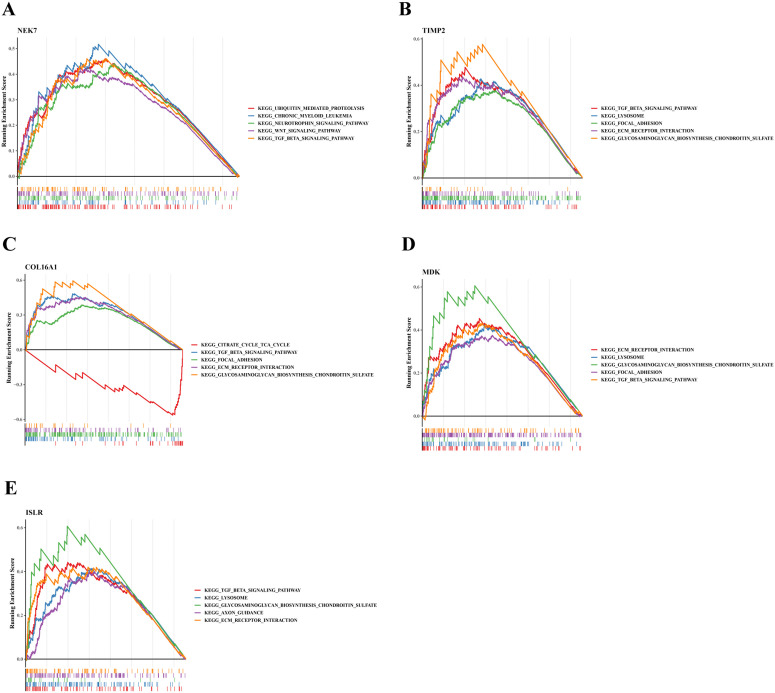
GSEA of Key Genes. (A) GSEA results for *NEK7*, showing enrichment in 20 pathways including TGFβ signaling pathway and WNT signaling pathway. (B) GSEA results for *TIMP2*, showing enrichment in 10 pathways including TGFβ signaling pathway and regulation of actin cytoskeleton. (C) GSEA results for *COL16A1*, showing enrichment in six pathways including TGFβ signaling pathway and ECM receptor interaction. (D) GSEA results for *MDK*, showing enrichment in seven pathways including ECM receptor interaction and TGFβ signaling pathway. (E) GSEA results for *ISLR*, showing enrichment in 12 pathways (e.g., TGFβ signaling pathway, Notch signaling pathway, and ECM receptor interaction).

The results of the GSEA revealed that the top five pathways significantly enriched by the biomarkers were as follows (*P*.adj < 0.05, |NES| > 1). *NEK7* showed potential correlative enrichment in 20 pathways, including the TGFβ signaling pathway and WNT signaling pathway (Fig 7A, S7 Table). *TIMP2* was associated with enrichment in 10 pathways (Fig 7B, S8 Table), such as the TGFβ signaling pathway and the regulation of the actin cytoskeleton. *COL16A1* was involved in six pathways (Fig 7C, S9 Table), including the TGFβ signaling pathway and ECM receptor interaction. *MDK* showed enrichment in seven pathways (Fig 7D, S10 Table), including ECM receptor interaction and the TGFβ signaling pathway. *ISLR* was enriched in 12 pathways (Fig 7E, S11 Table), involving the TGFβ signaling pathway, Notch signaling pathway, and ECM receptor interaction.

### Key genes were implicated in the immune microenvironment of HF

**Fig 8:**
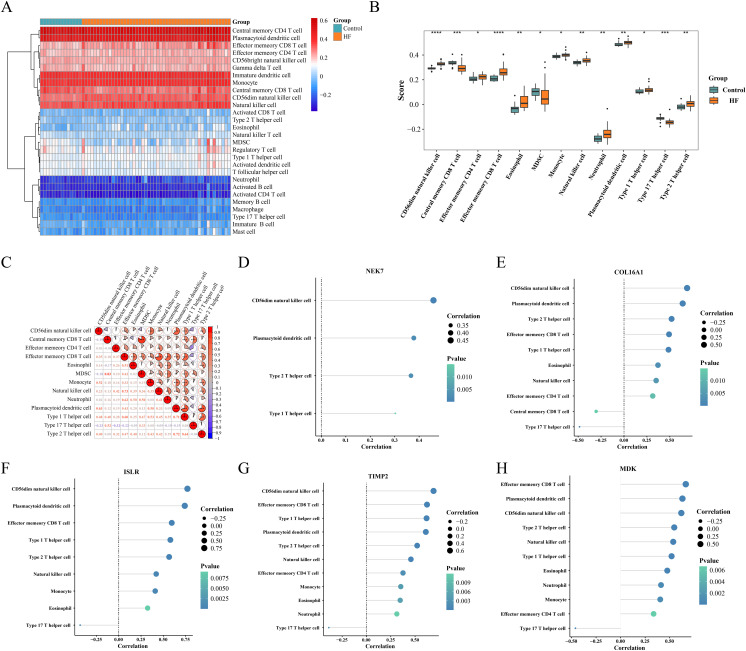
Analysis of Immune Microenvironment in HF. (A) Heatmap showing immune cell infiltration levels in the HF versus control groups. (B) Boxplot comparing 9 DIC types between the HF and control groups. (C) Heatmap showing the correlation between different immune cells. Red indicates positive correlation, purple indicates negative correlation, and the darker the color, the stronger the correlation. (D) Analysis of the Correlation between *NEK7* and divergent immune cells. The x-axis represents the correlation coefficient, and the y-axis represents the different immune cells. Lines pointing to the left indicate negative correlation, and lines pointing to the right indicate positive correlation. (E) Analysis of the Correlation between *COL16A1* and divergent immune cells. (F) Analysis of the Correlation between *ISLR* and divergent immune cells. (G) Analysis of the Correlation between *TIMP2* and divergent immune cells. (H) Analysis of the Correlation between *MDK* and divergent immune cells.

The heatmap (Fig 8A) displayed the abundances of 28 types of immune cell infiltration in the HF and control groups from dataset GSE116250. With a significance level of *P*.adj < 0.05, 9 DICs were identified, including CD56dim natural killer (NK) cells, central memory CD8 T cells, and effector memory CD8 T cells and so on (Fig 8B). The correlation between different immune cells is such that NK cells and Effector memory CD8 T cells show the highest positive correlation (cor = 0.73, *P* < 0.001; |cor| > 0.3, *P*.adj < 0.05) (Fig 8C). The analysis of the correlation between key genes and different immune cell subsets revealed that *NEK7*, *COL16A1*, and *ISLR* were all significantly positively correlated with CD56dim natural killer cells, with correlation coefficients of 0.46 (*P* < 0.001), 0.69 (*P* < 0.001), and 0.77 (*P* < 0.001), respectively (Fig 8D-F, S12 Table); *TIMP2* was significantly negatively correlated with type 17 helper T cells (r = −0.39, P < 0.001) (Fig 8G, S12 Table); and MDK was significantly negatively correlated with effector memory CD8 T cells (r = −0.66, *P* < 0.001) (Fig 8H, S12 Table).

### Molecular regulatory network construction, drug prediction, and molecular docking

As depicted in S1A Fig, in the TF-mRNA-miRNA regulatory network, *NEK7* was predicted to interact with nine TFs and 38 miRNAs, as indicated in S13 and S14 Tables, respectively. Similarly, *COL16A1* was associated with 13 TFs and three miRNAs, MDK with 11 TFs and four miRNAs, *ISLR* with six TFs and five miRNAs, and *TIMP2* with nine TFs and 35 miRNAs. Furthermore, NEK7 was associated with 16 potential drugs, COL16A1 with one, MDK with 15, ISLR with two, and TIMP2 with 25 potential drugs (S1B Fig, S15 Table). Additionally, molecular docking results demonstrated strong binding activities between WZ4002 and NEK7, beta-Heparin and MDK, and Oroxylin A and TIMP2. Carmustine was found to show certain binding activity with ISLR (S1C Fig).

### Annotation of 6 cell types via scRNA-seq data analysis

**Fig 9:**
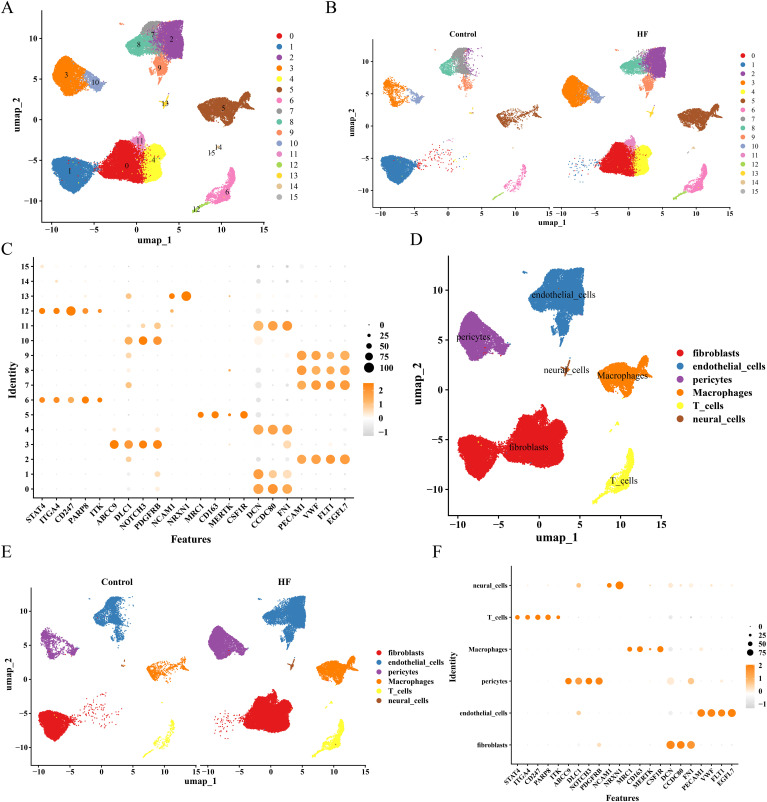
Cell Clustering and Annotation via scRNA-seq Data Analysis. (A) UMAP plot showing the clustering of cells into 16 distinct clusters. (B) UMAP plots comparing cell clusters in the control and HF groups. (C) Dot plot displaying the expression levels of marker genes for each cell type. (D) UMAP plot illustrating the annotation of six cell types: T cells, pericytes, neural cells, macrophages, fibroblasts, and endothelial cells. € UMAP plots comparing the distribution of annotated cell types between the control and HF groups. (F) Dot plot showing the identity scores of the annotated cell types.

Regarding the single-cell dataset GSE183852, prior to quality control, it comprised 49,042 cells and 36,517 genes (S2A Fig). After quality control, 48,392 cells remained with the gene count unchanged (S2B Fig). PCA identified the top 10 highly variable genes, including SPP1 and GNLY (S2C Fig). Analysis of 50 PCs revealed a stable contribution after the top 30; thus, these 30 were selected (*P* < 0.05; S2D-E Fig). Cell clustering categorized the cells into 16 clusters (Fig 9A-B), with marker gene expression illustrated in Fig 9C (S16 Table). The annotation process identified six cell types: T cells, pericytes, neural cells, macrophages, fibroblasts, and endothelial cells (Fig 9D-F).

### Key cell identification

**Fig 10:**
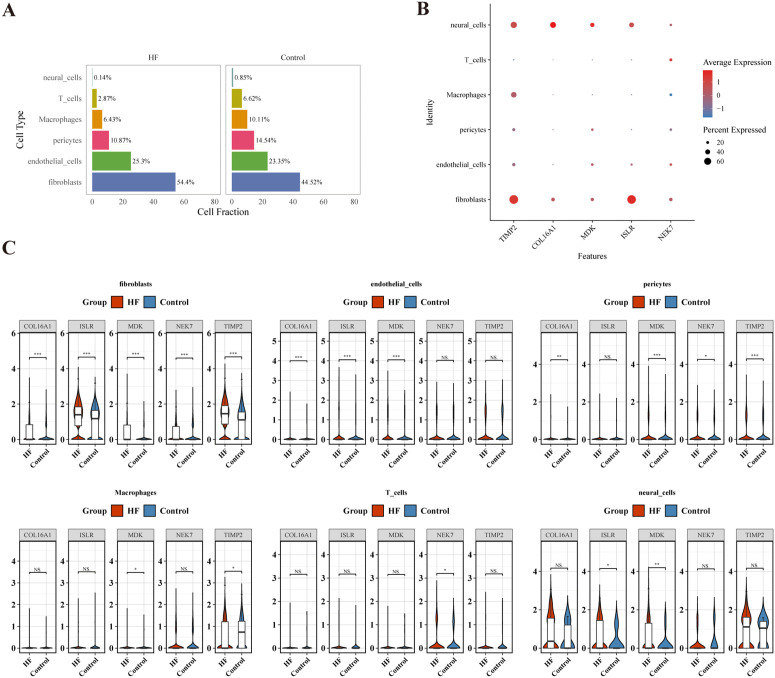
Identification of Key Cells in HF. (A) Proportions of various cell types in the HF and control groups, showing a high proportion of fibroblasts in both groups. (B) Expression levels of *NEK7* and key genes across different cell types, highlighting their high expression in fibroblasts and neural cells. (C) Differential expression analysis of *NEK7* and key genes between the HF and control groups in various cell types, demonstrating significant inter-group differences in fibroblasts.

The infiltration proportions of various cell types between the HF group and the control group indicated that fibroblasts constituted a significant proportion in both groups (Fig 10A). The analysis of expression levels of *NEK7* and key genes across different cell types revealed high expression in fibroblasts and neural cells (Fig 10B). Further analysis of expression differences for *NEK7* and key genes between the two groups across different cell types demonstrated inter-group differences in fibroblasts (Fig 10C). Consequently, fibroblasts were identified as key cells.

### Functional enrichment analysis combined with pseudotime analysis

Secondary clustering of fibroblasts, based on the top 30 principal components (S3A-B Fig), resolved the cells into nine distinct subclusters (S3C-D Fig). Pseudotime analysis identified 15 developmental stages: clusters 0, 2, 4, 6, and 8 dominated the early stages, while clusters 1, 3, 5, 7 dominated the late stages (S3E Fig). Pseudotime expression of key genes in fibroblasts demonstrated that *MDK/ISLR* levels increased in early stages and decreased in late stages; *COL16A1/NEK7* initially decreased then increased; TIMP2 declined continuously, linking these changes to disease development (S3F-G Fig). The FindAllMarkers function identified DEGs in HF samples, subgrouping key cells. The expression of the top 30 DEGs (S3H Fig) showed *BTNL9/VWF/CD36* higher in late stages, and *GJA4/ITGA7/NPY1R* higher in early stages. GO/KEGG enrichment of these 30 genes (S3I Fig) revealed 40 GO entries (28 BPs, 7 CCs, 5 MFs; *P*.adj < 0.05) including blood-brain barrier maintenance and endothelium development (S17 Table), and 80 KEGG pathways (*P*.adj < 0.05) such as cell adhesion molecules and ECM receptor interaction (S18 Table).

### Cell communication and TF regulatory analysis

Cell communication analysis indicated that fibroblasts, as key cells, were the most abundant in both groups. In the HF group, they engaged in 15 ligand-receptor pairs with the strongest interaction with macrophages (S4A Fig); in controls, they engaged in 13 pairs with the strongest macrophage interaction (S4B Fig). The probability of fibroblasts-to-macrophage communication was highest in both groups, with MIF-(CD74 + CD44) as the primary ligand-receptor pair (S4C-D Fig). TF activity analysis (S4E Fig) revealed that HNF4A, ATF3, EBF1 exhibited higher activity in HF, while SIX2, GATA6, JUN were more active in controls, suggesting distinct regulatory roles under physiological and pathological conditions.

### Validation of the Ang II-induced HF Model

**Fig 11:**
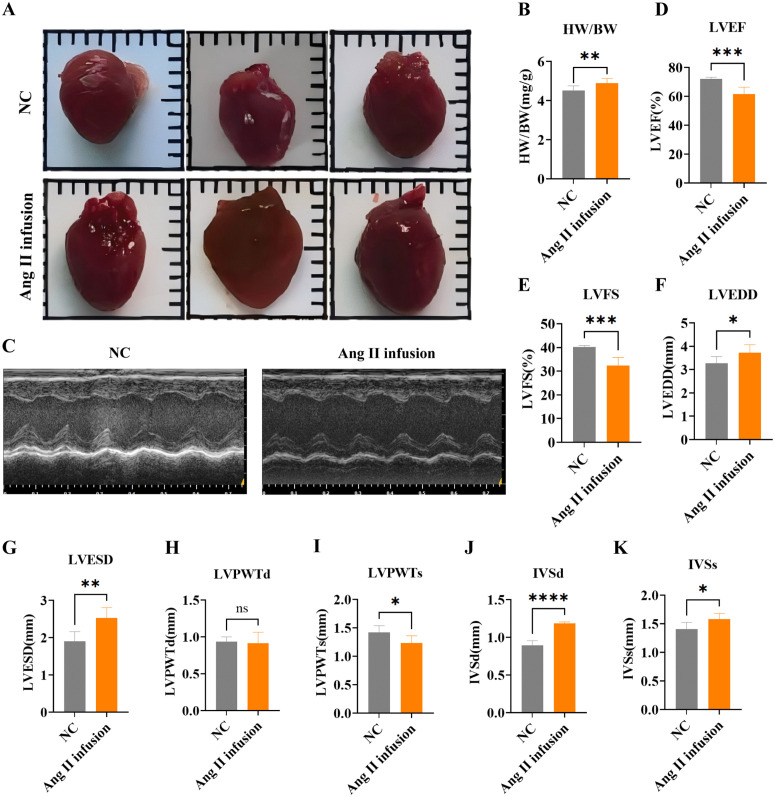
Successful establishment of the Ang II-induced HF model in mice. (A) Representative gross heart images showing cardiac enlargement. (B) HW/BW. (C) Representative M-mode echocardiograms. (D-G) Key functional and structural parameters: left ventricular ejection fraction (LVEF, D), fractional shortening (LVFS, E), end-diastolic diameter (LVEDD, F), and end-systolic diameter (LVESD, G). (H-K) Wall thickness parameters: diastolic left ventricular posterior wall (LVPWTd, H), systolic left ventricular posterior wall (LVPWTs, I), diastolic interventricular septum (IVSd, J) systolic interventricular septum (IVSs, K). (*P < 0.05, **P < 0.01, ***P < 0.001, ****P < 0.0001).

Cardiac function and structure were evaluated using echocardiography and gross morphological assessments. Mice subjected to Ang II infusion demonstrated cardiac enlargement and a significantly increased HW/BW (*P* < 0.01, Fig 11A, B). Analysis of cardiac function revealed systolic dysfunction, characterized by a significant decrease in LVEF (*P <* 0.001, Fig 11D). Additionally, structural remodeling was evident, as indicated by significant increases in both LVEDD and LVESD (both *P* < 0.001, Fig 11F, G). The interventricular septum displayed a tendency towards hypertrophy, marked by significant increases in IVSd (*P* < 0.0001, Fig 11J) and IVSs (*P* < 0.05, Fig 11K), whereas the LVPWTd remained unchanged (Fig 11H). Collectively, these functional and structural changes confirm the successful establishment of the Ang II-induced murine HF model.

### Key genes expression

**Fig 12:**
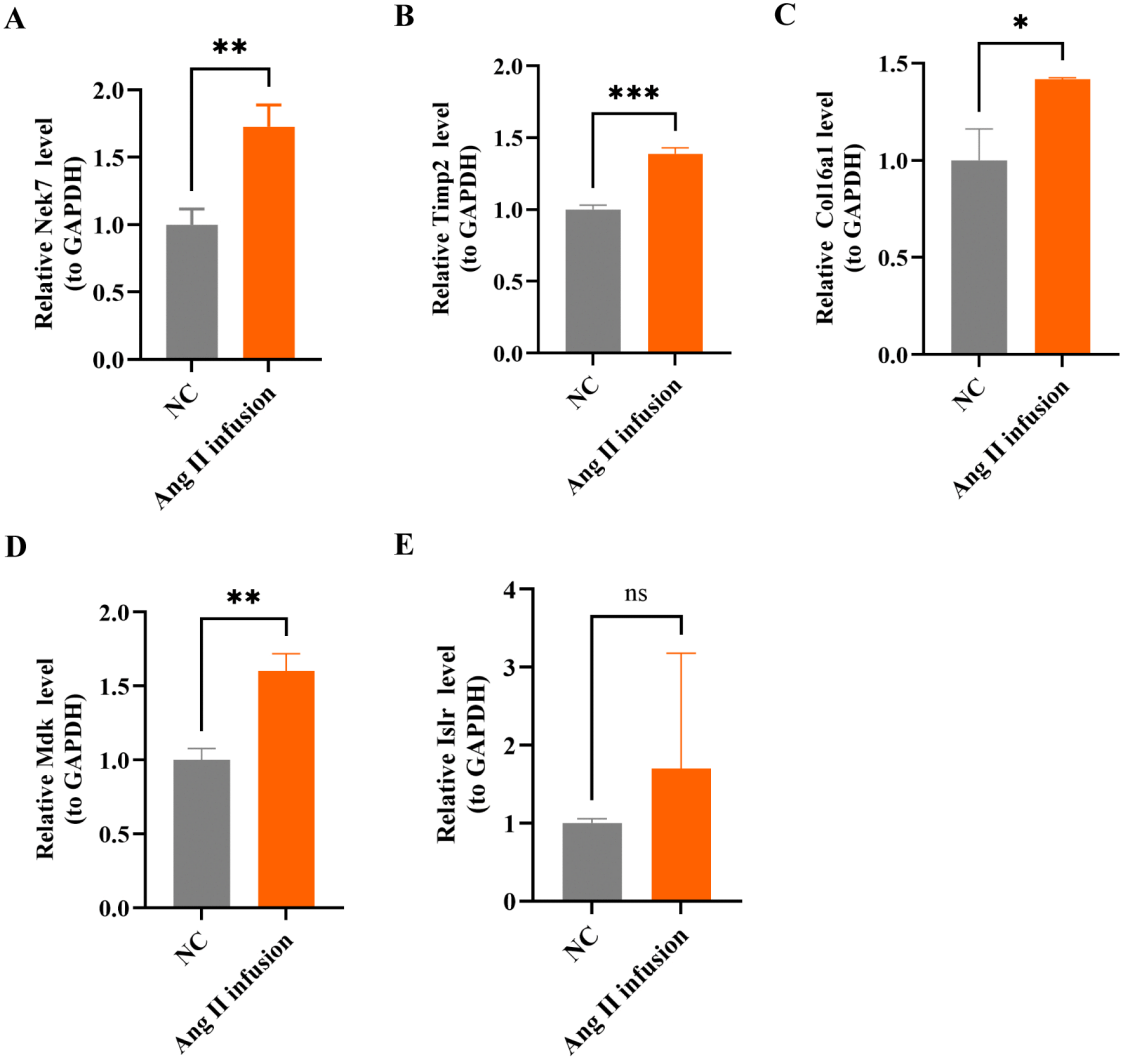
Relative mRNA expression levels of key genes in Ang II-induced HF model. The relative mRNA expression levels of *Nek7* (A), *Timp2* (B), *Col16a1* (C), *Mdk* (D), and *Islr* (E) were quantified by qPCR, normalized against *GAPDH*, and presented as mean±SEM. The results show significant upregulation of *Nek7* (*P* < 0.01), *Timp2* (*P <* 0.001), *Col16a1* (*P* < 0.05), and *Mdk* (*P <* 0.01) in the Ang II infusion group compared to the controls. The expression of *Islr* displayed an upward trend but was not statistically significant (ns, *P* = 0.5691). (*P < 0.05, **P < 0.01, ***P < 0.001, ****P < 0.0001). Note: Animal experiments in this section had a sample size of n = 3 per group, and only mRNA-level detection was performed; the results are preliminary findings.

Following the validation of the HF model (Fig 11), we evaluated the transcriptional changes in myocardial tissue. The results from RT-qPCR indicated that, in comparison to the NC group, the Ang II infusion group exhibited significantly higher expression levels of *Nek7* (Table 3, Fig 12A), *Timp2* (Fig 12B), *C0l16a1* (Fig 12C), and *Mdk* (Fig 12D) (*P* < 0.05). However, the expression of *Islr* (Fig 12E), although elevated, did not reach statistical significance (primer sequences are provided in S19 Table). These expression patterns of the key genes in the HF group were consistent with the findings from the analysis of public databases.

**Table 3:** Differential gene expression in Ang II-induced HF.

Gene	NC	Ang II infusion	*P* value
*Nek7*	1.0000 ± 0.1159	1.7252 ± 0.1634	0.0033
*Timp2*	1.0000 ± 0.0315	1.3883 ± 0.0412	0.0002
*C0l16a1*	1.0000 ± 0.1624	1.4185 ± 0.0069	0.0112
*Mdk*	1.0000 ± 0.0779	1.6018 ± 0.1176	0.0018
*Islr*	1.0000 ± 0.0540	1.7017 ± 1.4760	0.5691

Note: Animal experiments in this table had a sample size of n = 3 per group, and only mRNA-level detection was performed; the results are preliminary findings.

Preliminary detection of mRNA expression for key genes in the murine model aligns with trends observed in transcriptomes from human HF tissues (*NEK7, TIMP2, COL16A1, MDK, ISLR*), providing preliminary support for the pivotal roles these genes play in the pathogenesis of HF. The lack of significant difference in Islr expression, however, warrants further investigation. Nevertheless, the animal experimental results provide preliminary support for the conclusions drawn from bioinformatics analyses, emphasizing the critical roles of these key genes in the development of HF.

## Discussion

NEK7 mediates NLRP3 activation under various pathological conditions, thereby promoting pathological myocardial remodeling—a core mechanism underlying heart failure [45,46]. Given that NLRP3 has been established to play a critical role in the pathogenesis of heart failure [47], it is plausible that NEK7 contributes to heart failure progression through the regulation of NLRP3 inflammasome signaling. Patients with chronic HF exhibit elevated markers of platelet activation [48]; however, whether these two pathological processes are functionally interconnected—and if so, through which molecular mediators—remains largely unexplored. In this study, we identified a four-gene signature (*TIMP2*, *COL16A1*, *MDK*, and *ISLR*) that exhibits statistically significant co-expression with both NEK7 and a set of platelet activation-related genes in the human HF transcriptome. This co-expression pattern suggests a potential correlative association of these genes at the interface of inflammation, thrombosis, and fibrosis, without direct causal evidence.

A central finding of our study is the consistent and coordinated enrichment of all four key genes within the TGF-β signaling pathway, as revealed by GSEA. This strongly suggests that the TGF-β pathway serves as a common orchestrator, synchronizing the expression and functions of *TIMP2*, *COL16A1*, *MDK*, and *ISLR* into a synergistic pro-fibrotic network. TGF-β is a pivotal regulator of cardiac fibrosis, activating fibroblasts and leading to ECM accumulation and myocardial stiffness [49]. It exhibits biphasic roles: initially exerting anti-inflammatory effects that promote scar formation, but with persistent late-stage activation, it drives maladaptive fibrosis and dysfunction [50,51]. Our data position this four-gene module as a critical executor of TGF-β’s detrimental late-phase effects in HF.

Within this module, each gene contributes distinct yet complementary roles to the fibrotic process, underpinning their collective identification and utility as a diagnostic signature. *TIMP2*, a central ECM regulator, is upregulated under cardiac stress as a compensatory mechanism to delay ECM degradation [52]. Our pseudotime analysis revealed a continuous downregulation of *TIMP2* during fibroblast differentiation, suggesting that its depletion in advanced HF may release MMP inhibition, leading to uncontrolled ECM degradation. COL16A1, a non-fibrillar collagen, is significantly upregulated in HF and stabilizes the matrix structure [53]. Our study confirms its high fibroblast expression and association with M2 macrophage infiltration [54,55], suggesting a cross-talk that influences ECM organization. MDK, known for its dual roles in cardiovascular disease, either facilitates reparative angiogenesis [56] or promotes inflammation and fibrosis [57]. Our findings reveal a significant correlation between MDK expression and myeloid-derived suppressor cell (MDSC) infiltration, suggesting a potential immunomodulatory role in HF.

Bioinformatic analyses consistently implicated *ISLR* within the fibro-inflammatory module; however, its mRNA expression in our murine model of HF, while trending upward, did not achieve statistical significance (*P =* 0.5691). Several factors might have contributed to this result. Firstly, the limited sample size in our animal experiment (n = 3 per group) likely diminished the statistical power necessary to detect subtle, yet biologically significant changes. Secondly, inherent biological variability or potential species-specific differences in the regulation of *ISLR*, compared to its human ortholog, cannot be ruled out. This finding highlights the challenges associated with translating human transcriptomic signatures to animal models. Importantly, its homologous gene, *Meflin*, is a well-established inhibitor of fibrosis [58,59]. Thus, the ISLR/Meflin axis might represent a built-in counter-regulatory mechanism within this module, fine-tuning expression to modulate, rather than solely promote, the fibrotic response. Future studies with larger experimental cohorts are essential to definitively clarify the role and regulation of ISLR in HF. The potential dual role of ISLR adds a layer of complexity to our model and necessitates further investigation at both the protein level and across extended disease time courses. The simultaneous dysregulation of these four interacting genes provides a more comprehensive pathophysiological snapshot than any single biomarker alone.

The clinical relevance of this gene module is further highlighted by its central role in the cellular interactome of HF. Fibroblasts, which constitute approximately 70% of cardiac cells [60], were identified as the primary expressors of our gene signature. The differential expression patterns of these biomarkers across fibroblast developmental stages underscore the spatiotemporal complexity of their roles in HF progression. Cell communication analysis revealed that fibroblasts, equipped with this pro-fibrotic and pro-inflammatory gene module, had the most pronounced interactions with macrophages via the MIF-(CD74 + CD44) ligand-receptor pair in both HF and control groups, suggesting that this module may facilitate critical intercellular dialogue between fibrosis and inflammation. The MIF-(CD74 + CD44) ligand-receptor pair represents the most predominant intercellular communication axis between fibroblasts and macrophages in heart failure, a finding that is highly consistent with the significant upregulation of MIF in fibroblasts and its positive correlation with the expression of the four-gene signature. As a pleiotropic chemokine, MIF has been demonstrated to promote macrophage polarization toward a pro-inflammatory phenotype and impair their tissue-resident migratory capacity, thereby exacerbating myocardial fibrosis [61,62]. This study is the first to link MIF signaling with the co-expression module: this gene module may activate fibroblasts and upregulate MIF secretion, which in turn remodels macrophage function in a paracrine manner, establishing a positive “fibroblast-macrophage” feedback loop that amplifies local inflammation and extracellular matrix deposition. Therefore, MIF not only serves as a hub molecule bridging innate immunity and fibrotic progression but may also function as a downstream effector of the four-gene signature, synergistically driving adverse remodeling in HF. Targeting MIF or its receptor complex could potentially interrupt this vicious cycle, offering a novel strategy for intervening in the downstream effects of the NEK7-platelet-inflammation axis.

Beyond the interplay between fibroblasts and macrophages, our immune infiltration analysis further revealed a significant positive correlation between NEK7 expression and the infiltration of CD56dim NK cells, suggesting a potential regulatory role of this immune subset within the NEK7-NLRP3 inflammasome-platelet axis. Although this correlative finding itself requires direct experimental validation, it generates an integrated hypothesis for a potential “NEK7-platelet-fibrosis” axis in HF: Upon activation, platelets release PAF, which has been shown to activate the NEK7-NLRP3 inflammasome [63] and may also chemoattract and activate CD56dim NK cells. In the HF microenvironment, platelet-derived PAF may serve as a molecular bridge linking platelet hyperreactivity, NEK7-mediated inflammatory signaling, and the recruitment of CD56dim NK cells. The latter, in turn, may exacerbate endothelial injury, fibroblast activation, and extracellular matrix remodeling through the release of perforin, granzymes, and pro-inflammatory cytokines [64]. This hypothesis integrates our immune infiltration findings with the core pathogenic axis and provides a theoretical basis for future intervention strategies targeting this pathway. Notably, the four-gene signature transcriptomic (*TIMP2*, *COL16A1*, *MDK*, and *ISLR*) identified in our study—each significantly enriched in the TGF-β signaling pathway and predominantly expressed in fibroblasts—may be potentially correlated with this putative axis as downstream correlative factors. Their coordinated upregulation in HF and co-expression with *NEK7* and platelet activation-related genes raise the possibility that they execute fibrogenic programs potentially initiated by PAF-mediated crosstalk among platelets, the NEK7 inflammasome, and CD56dim NK cells. While speculative, these observations suggest a potential “NEK7-platelet-fibrosis” axis in HF, wherein platelet-derived PAF may bridge NEK7-mediated inflammation and NK cell recruitment, ultimately contributing to fibroblast activation and ECM remodeling through this TGF-β‑centered gene module. This hypothesis-generating framework integrates inflammation, thrombosis, and fibrosis in HF pathogenesis and warrants direct experimental validation in future studies.

However, several limitations of this study should be acknowledged. Firstly, the proposed “NEK7-platelet-fibrosis” axis is primarily based on bioinformatic co-expression analyses and lacks direct experimental validation, such as platelet activation assays or experiments involving platelet-cardiac cell interactions. All mechanistic inferences are correlative and require direct functional validation. Secondly, although we verified the comparability of GSE5406 with the training set and initial validation set via baseline characteristic comparison and batch effect correction, potential batch effects in public datasets cannot be completely excluded. Therefore, further validation in prospective, multi-center cohorts is necessary. Thirdly, we have mitigated overfitting risk via L2 regularization, 10-fold repeated cross-validation, 1000-times bootstrap optimism correction, and cross-dataset calibration assessment, which suggest model stability and generalizability. Nevertheless, the limited sample size of the discovery dataset means that some degree of overfitting risk remains, and external validation in larger independent cohorts is required. Fourthly, the in vivo validation was constrained by a small sample size (n = 3 per group) and the absence of protein-level validation (e.g., Western blot, immunohistochemistry). Therefore, the animal data should be interpreted as preliminary support rather than confirmatory validation. In addition, although *ISLR* showed a consistent upward trend, it did not reach statistical significance, and potential underlying reasons-such as species differences, temporal dynamics, cellular heterogeneity, or post-transcriptional regulation-were not further explored. Fifthly, while the candidate diagnostic nomogram achieved good performance in our training set, its clinical utility was evaluated only in distinguishing HF patients from healthy controls using myocardial tissue samples. The detectability of the key genes in accessible samples such as blood remains unclear, and the model has not been validated in prospective cohorts or real-world diagnostic settings. Finally, the drug prediction analysis was based solely on molecular docking simulations; candidate compounds identified (e.g., β-heparin, WZ4002) lack functional validation in vitro or in vivo, and their true therapeutic potential remains to be elucidated.

Given the limitations outlined above, future research should systematically advance along two complementary dimensions: mechanistic validation and clinical translation. At the mechanistic level, larger-scale animal studies incorporating multiple HF models and temporal sampling are warranted to validate the robustness and dynamic expression patterns of the four-gene signature. Protein-level validation via Western blot, multiplex immunohistochemistry, and interrogation of public proteomic databases should be performed to confirm the expression and cellular sources of these key genes in myocardial tissue. The causal role of *ISLR* in fibroblast activation and fibrogenesis merits in-depth investigation using CRISPR-Cas9-based approaches. Furthermore, platelet–fibroblast co-culture systems combined with genetic intervention strategies should be employed to directly test whether platelet activation induces this gene module and promotes pro-fibrotic phenotypes. On the clinical translation front, prospective, multicenter cohort studies are needed to systematically evaluate the candidate diagnostic nomogram’s performance in early screening, differential diagnosis of HFpEF, and prognostic prediction. Net reclassification improvement analyses should be conducted to quantify its incremental value over conventional biomarkers. A critical priority is to assess the feasibility of detecting the key genes in accessible samples such as blood (e.g., plasma proteins, exosomal mRNA) and to establish tissue–blood expression correlations, thereby facilitating the transition of this signature from a tissue-dependent model toward a non-invasive liquid biopsy tool. Additionally, future bioinformatic analyses should rigorously apply corrections for multiple testing to control false-positive rates. Finally, the candidate compounds identified through molecular docking warrant functional validation in fibroblast activation assays in vitro and efficacy evaluation in animal models in vivo. Beyond monotherapy approaches, multi-target combination strategies concurrently targeting the NEK7 inflammasome, TGF-β signaling, and ECM remodeling should be explored. Collectively, these systematic investigations will progressively transform the current transcriptomic association-based hypothesis into a multi-dimensionally validated pathophysiological framework, providing a robust evidence base for translating this gene signature into clinical diagnostic tools.

## Conclusions

This study generates a potential integrated mechanistic hypothesis for HF: *NEK7* and platelet activation are potentially correlated with the coordinated upregulation of a TGF-β-centric four-gene transcriptomic signature (*TIMP2*, *COL16A1*, *MDK*, *ISLR*), which may be associated with fibrotic signaling and immune-fibroblast crosstalk correlated with maladaptive remodeling. The strong performance of this candidate gene signature in our cohort underscores its potential clinical significance. Functional analyses have revealed their synergistic involvement in TGF-β signaling and immune-fibroblast crosstalk, with fibroblasts acting as central expressors. Animal experimental results provide preliminary in vivo validation support for the above mechanisms and biomarker conclusions. This study is a hypothesis-generating transcriptomic analysis that identifies a potential transcriptomic signature and provides an integrated correlative framework for HF pathogenesis. All mechanistic inferences require direct functional experimental validation, and the preliminary discrimination model has no clinical diagnostic value. This work provides a direction for subsequent mechanistic research and exploratory biomarker studies.

## Supporting information

S1 Fig Molecular Regulatory Network, Drug Prediction, and Molecular Docking. (A) TF-mRNA-miRNA regulatory network showing interactions between key genes (*NEK7*, *COL16A1*, *MDK*, *ISLR*, *TIMP2*) with TFs and miRNAs. (B) Predicted potential drugs for each key gene. (C) Molecular docking results demonstrating strong binding activities of beta-Heparin with MDK, Oroxylin A with TIMP2, WZ4002 with NEK7, and certain binding activity of Carmustine with ISLR.
(TIF)

S2 Fig Single-cell analysis. (A) Before quality control. Note: nFeature_RNA: the number of genes detected in each cell, nCount_RNA: the expression count of genes detected in each cell. (B) After quality control. (C) Screening of highly variable genes. Note: The horizontal axis represents the expression level of genes, and the vertical axis represents the highly variable status of genes. The red dots in the figure represent the first 2000 highly variable genes. (D) PCA results. Each line represents one principal component. (E) On the right is the scatter plot. The horizontal axis represents the principal component, and the vertical axis represents “Standard Deviation,” which is used to describe the degree of dispersion or volatility of the data.
(TIF)

S3 Fig Single-cell analysis. (A) The results of the secondary clustering of fibroblasts based on the first 30 PCA components. (B) Scatter plot. The abscissa represents the principal component, and the ordinate represents “Standard Deviation,” which is the standard deviation and is used to describe the degree of dispersion or volatility of the data. (C-D) UMAP plots showing the clustering of fibroblasts into nine clusters in the control and HF groups. (E) Pseudotime analysis dividing cell developmental states into 15 stages, with clusters 0, 2, 4, 6, and 8 primarily in early stages and clusters 1, 3, 5, and 7 in late stages. (F) Pseudotemporal expression dynamics of key genes across fibroblast subclusters. (G) Pseudotime expression analysis of key genes (*COL16A1*, *ISLR*, *MDK*, *NEK7*, *TIMP2*) at different time stages in fibroblasts, revealing their expression patterns during differentiation. (H) Heatmap displaying the top 30 DEGs in different subgroups of key cells, highlighting differential expression between early and late differentiation stages. (I) Pseudotemporal expression dynamics of key genes across fibroblast subclusters.
(TIF)

S4 Fig Cell Communication and TF Regulatory Analysis. (A-B) Ligand-receptor interaction networks in the HF and control groups, showing fibroblasts as key cells with the highest number of ligand-receptor pairs and strongest interactions with macrophages. (C-D) Communication probability between fibroblasts and other cell types, highlighting the MIF-(CD74 + CD44) ligand-receptor pair as the primary interaction in both groups. (E) Heatmap displaying the activities of different TFs in the HF and control groups, indicating distinct regulatory effects under different conditions, such as higher activity of HNF4A, ATF3, and EBF1 in the HF group and *SIX2*, *GATA6*, *JUN* in the control group.
(TIF)

S1 Table Complete list of 1,073 DEGs in HF versus controls from GSE116250 dataset. (XLSX)

S2 Table Comprehensive list of 261 PCRGs used for intersection analysis with HF DEGs. (XLSX)

S3 Table ssGSEA enrichment scores of 14 differentially expressed platelet-related genes in HF versus control groups. (XLSX)

S4 Table 1121 key module genes from the WGCNA turquoise module selected by stringent thresholds (|MM| > 0.8 and |GS| > 0.2) for subsequent analysis. (XLSX)

S5 Table Complete list of 237 significantly enriched GO terms for 26 candidate genes. (XLSX)

S6 Table Significantly enriched KEGG pathways for 26 candidate genes. (XLSX)

S7 Table Complete list of 20 significantly enriched pathways for NEK7 from GSEA analysis. (XLSX)

S8 Table 10 significantly enriched pathways for TIMP2 from GSEA analysis. (XLSX)

S9 Table Six significantly enriched pathways for COL16A1 from GSEA analysis. (XLSX)

S10 Table Seven significantly enriched pathways for MDK from GSEA analysis. (XLSX)

S11 Table 12 significantly enriched pathways for ISLR from GSEA analysis. (XLSX)

S12 Table Complete correlation matrix between NEK7 expression and immune cell infiltration profiles in HF versus controls (GSE116250 dataset). (XLSX)

S13 Table Predicted TFs regulating key biomarkers in the TF-mRNA network. (XLSX)

S14 Table Predicted miRNAs targeting key biomarkers in the miRNA-mRNA network. (XLSX)

S15 Table Potential therapeutic drugs targeting key biomarkers identified through drug prediction analysis. (XLSX)

S16 Table Marker genes and expression profiles for 16 cell clusters identified by single-cell RNA sequencing in GSE183852 dataset. (XLSX)

S17 Table Complete GO enrichment for 30 key cell differentiation-related genes across differentiation stages. (XLSX)

S18 Table 80 significantly enriched KEGG pathways for 30 key cell differentiation-related genes. (XLSX)

S19 Table Primer sequences used for RT-qPCR validation of key biomarkers in HF models. (XLSX)
