# Supplementary figures and images for: Integrated NEK7-inflammasome and platelet transcriptomic signature generates mechanistic hypotheses in heart failure
Source: PLoS One. 2026 Jun 26;21(6):e0352124. doi: 10.1371/journal.pone.0352124 (PMC13308797; doi:10.1371/journal.pone.0352124)

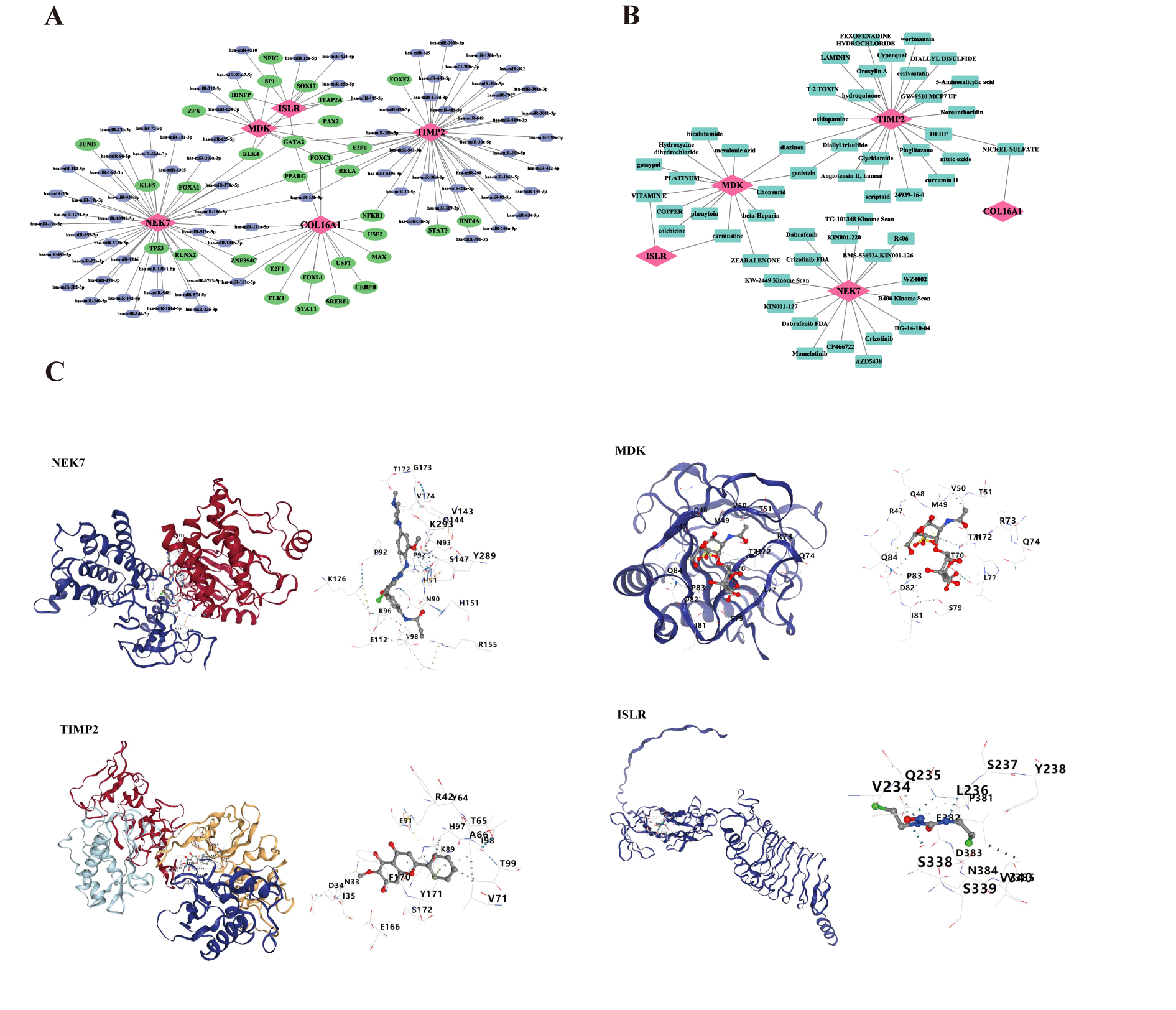

Supplement: S1 Fig — (A) TF-mRNA-miRNA regulatory network showing interactions between key genes (NEK7, COL16A1, MDK, ISLR, TIMP2) with TFs and miRNAs. (B) Predicted potential drugs for each key gene. (C) Molecular docking results demonstrating strong binding activities of beta-Heparin with MDK, Oroxylin A with TIMP2, WZ4002 with NEK7, and certain binding activity of Carmustine with ISLR. (TIF) [file pone.0352124.s001.tif]

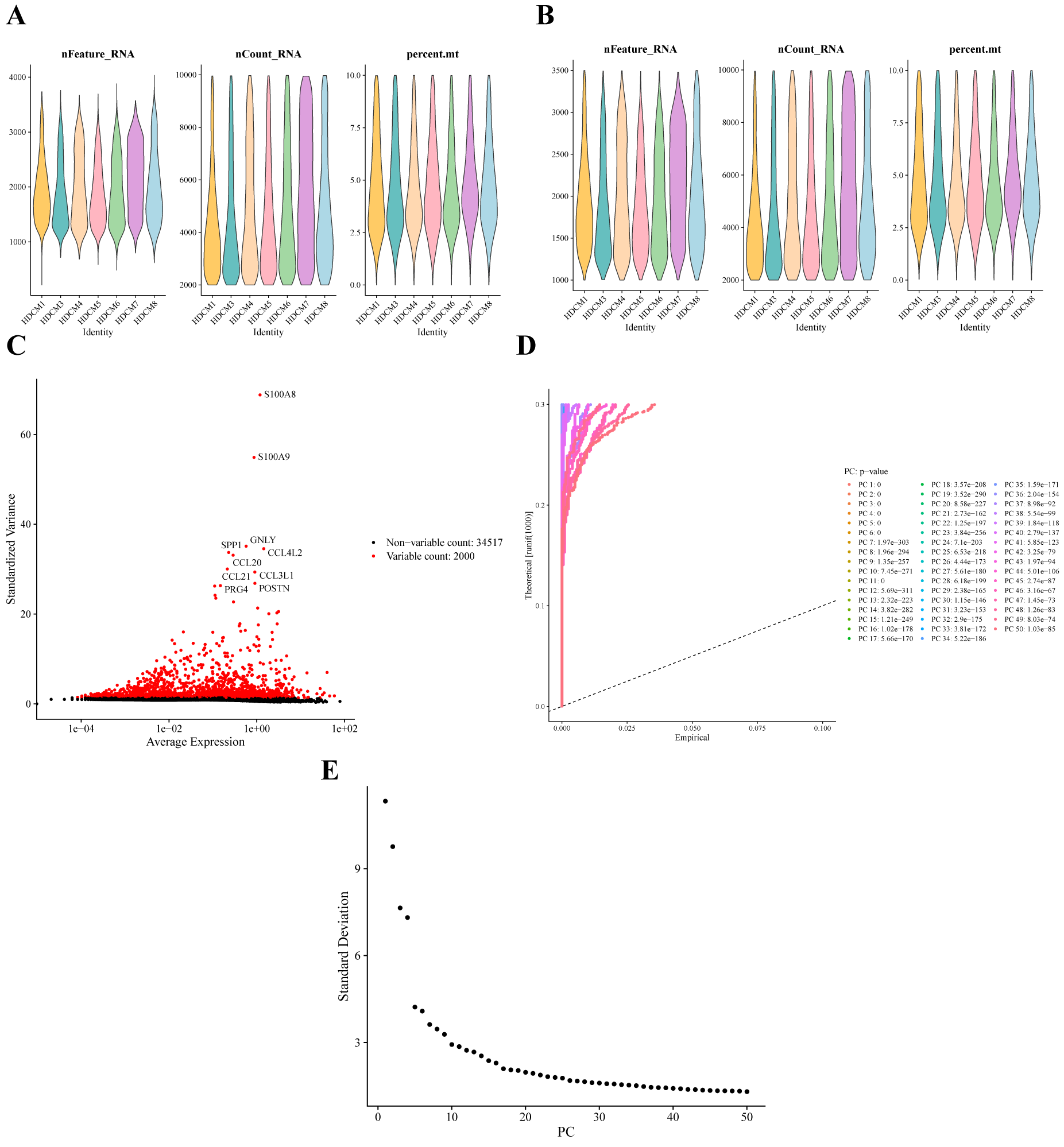

Supplement: S2 Fig — (A) Before quality control. Note: nFeature_RNA: the number of genes detected in each cell, nCount_RNA: the expression count of genes detected in each cell. (B) After quality control. (C) Screening of highly variable genes. Note: The horizontal axis represents the expression level of genes, and the vertical axis represents the highly variable status of genes. The red dots in the figure represent the first 2000 highly variable genes. (D) PCA results. Each line represents one principal component. (E) On the right is the scatter plot. The horizontal axis represents the principal component, and the vertical axis represents “Standard Deviation,” which is used to describe the degree of dispersion or volatility of the data. (TIF) [file pone.0352124.s002.tif]

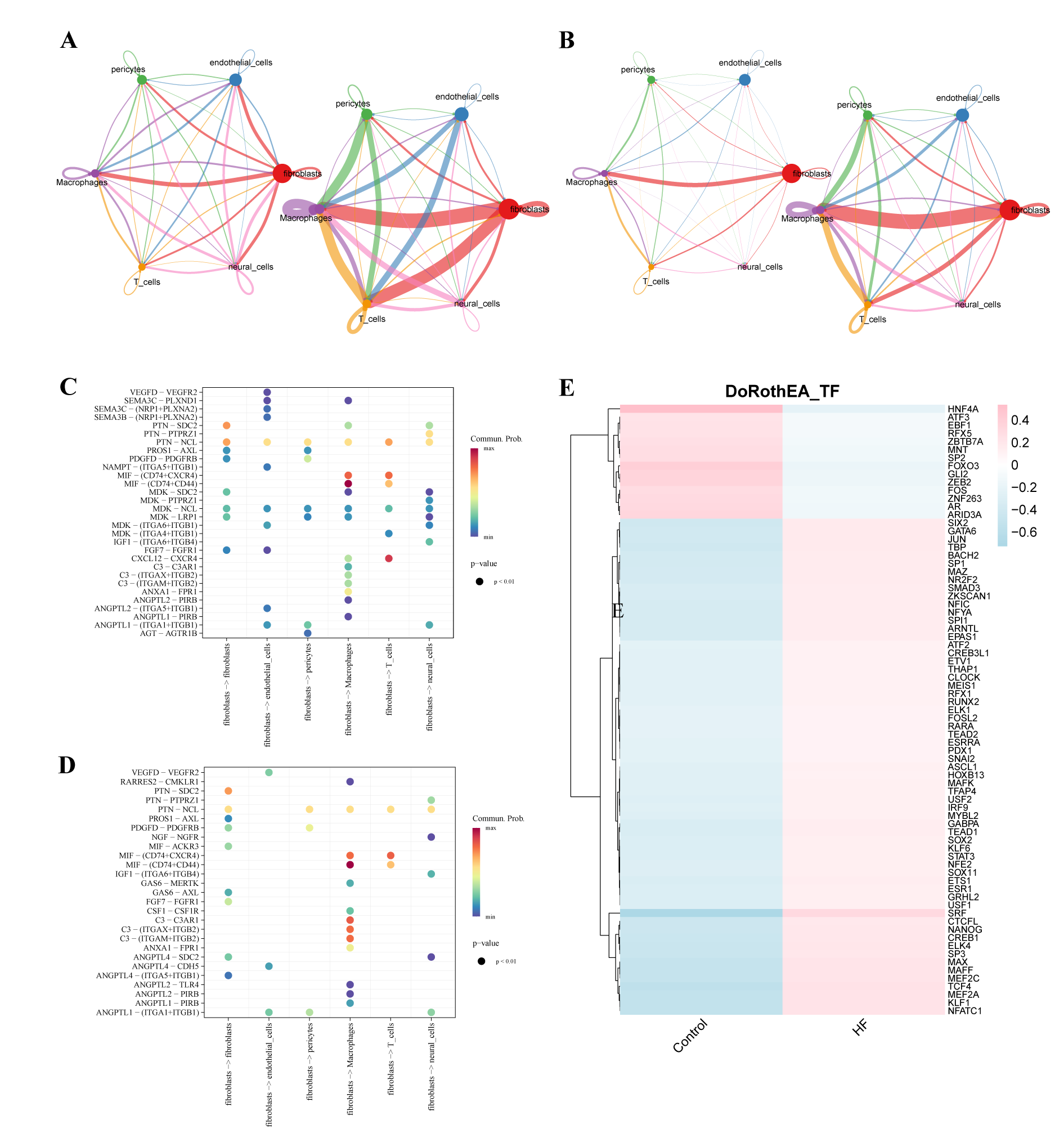

Supplement: S4 Fig — (A-B) Ligand-receptor interaction networks in the HF and control groups, showing fibroblasts as key cells with the highest number of ligand-receptor pairs and strongest interactions with macrophages. (C-D) Communication probability between fibroblasts and other cell types, highlighting the MIF-(CD74 + CD44) ligand-receptor pair as the primary interaction in both groups. (E) Heatmap displaying the activities of different TFs in the HF and control groups, indicating distinct regulatory effects under different conditions, such as higher activity of HNF4A, ATF3, and EBF1 in the HF group and SIX2, GATA6, JUN in the control group. (TIF) [file pone.0352124.s004.tif]
