# Supplementary material for: Integrated NEK7-inflammasome and platelet transcriptomic signature generates mechanistic hypotheses in heart failure
Source: PLoS One. 2026 Jun 26;21(6):e0352124. doi: 10.1371/journal.pone.0352124 (PMC13308797; doi:10.1371/journal.pone.0352124)
Supplement: Attachment — Submitted filename: Response to Reviewers.docx [file pone.0352124.s024.docx]

Point-by-point response to the reviewer comments.

Dear Reviewers,

We are grateful for your thoughtful comments and constructive suggestions, which have been instrumental in improving the quality of our manuscript. We look forward to collaborating with you as we advance this work toward publication in PLOS ONE.

In response to your feedback, we have carefully revised the manuscript. Our point-by-point responses to all comments are provided below. We have strived to address all the issues raised and hope that the revised manuscript now meets the journal's standards for publication. If you have any questions remaining about this paper, please feel free to contact us.

**Reviewers' comments:**

Reviewer's Responses to Questions

Comments to the Author

1. Is the manuscript technically sound, and do the data support the conclusions?

The manuscript must describe a technically sound piece of scientific research with data that supports the conclusions. Experiments must have been conducted rigorously, with appropriate controls, replication, and sample sizes. The conclusions must be drawn appropriately based on the data presented.

Reviewer #1: Partly

Reviewer #2: Yes

**Re:** We sincerely appreciate the reviewers' constructive feedback on the technical soundness and data-conclusion alignment of our study. In response to the concerns raised, we have strengthened the experimental rigor by adding independent replication data and enhancing control designs (**lines 333 of the manuscript**), explicitly addressed the influence of sample size on generalizability in the discussion, moderated the conclusions to a more inferential tone, and elaborated methodological details for improved reproducibility (**lines 138–139, 157–175 of the manuscript**). These revisions ensure that all conclusions are appropriately supported by the data presented. We are grateful for the insightful comments that have enhanced the manuscript's scientific rigor.

2. Has the statistical analysis been performed appropriately and rigorously?

Reviewer #1: Yes

Reviewer #2: Yes

**Re:** We sincerely thank the reviewers for their positive assessment of the appropriateness and rigor of the statistical analyses in our study. Proper and rigorous statistical analysis is indeed fundamental to ensuring the reliability of research conclusions, and throughout the study, we have strictly adhered to standard statistical procedures and maintained a scientifically rigorous approach. Your positive feedback not only greatly encourages our previous efforts but also reinforces our commitment to further improving the manuscript. We will continue to uphold this standard of rigor and carefully address all reviewer comments to further enhance the quality of the manuscript. Thank you once again for your professional review and thoughtful guidance.

3. Have the authors made all data underlying the findings in their manuscript fully available?

The PLOS Data policy requires authors to make all data underlying the findings described in their manuscript fully available without restriction, with rare exception (please refer to the Data Availability Statement in the manuscript PDF file). The data should be provided as part of the manuscript or its supporting information, or deposited to a public repository. For example, in addition to summary statistics, the data points behind means, medians and variance measures should be available. If there are restrictions on publicly sharing data—e.g. participant privacy or use of data from a third party—those must be specified.

Reviewer #1: Yes

Reviewer #2: Yes

**Re：**We grateful for the reviewers' recognition of the data availability underlying our study. We fully acknowledge that complete and unrestricted public sharing of data is central to PLOS’s data policy and essential for ensuring research transparency and reproducibility. In accordance with these guidelines, we have deposited all core data in a public repository with clear access information and provided detailed descriptions in the manuscript. Your recognition reinforces our commitment to open science principles. We will continue to adhere strictly to these standards and further refine data-related statements in the manuscript. Thank you once again for your professional review and thoughtful guidance.

4. Is the manuscript presented in an intelligible fashion and written in standard English?

PLOS ONE does not copyedit accepted manuscripts, so the language in submitted articles must be clear, correct, and unambiguous. Any typographical or grammatical errors should be corrected at revision, so please note any specific errors here.

Reviewer #1: Yes

Reviewer #2: Yes

**Re：**We are greatly encouraged by the reviewers’ f favorable evaluation of the clarity and linguistic standards of our manuscript. To further ensure compliance with PLOS ONE's language standards, we have engaged a professional academic editing service to refine the expression, grammar, and overall readability of the text while fully preserving its scientific content. We are committed to submitting a manuscript that meets the journal’s requirements for clear, correct, and unambiguous academic English.

5. Review Comments to the Author

Please use the space provided to explain your answers to the questions above. You may also include additional comments for the author, including concerns about dual publication, research ethics, or publication ethics. (Please upload your review as an attachment if it exceeds 20,000 characters)

**Reviewer #1:**

Dear Authors,

The manuscript addresses an important question and uses appropriate multi-omics and machine-learning approaches; however, major revision is required before it can be considered for publication.

1. Please reduce the length of the manuscript, particularly the Results section. Several downstream analyses (e.g., extensive pseudotime analysis, cell–cell communication, TF–miRNA networks, and molecular docking) can be condensed or moved to the Supplementary Materials.

**Re：**We sincerely appreciate your constructive suggestions regarding the length and structure of our manuscript. In accordance with your recommendations, we have streamlined the Results section by relocating detailed downstream analyses—including extensive pseudotime analysis, cell–cell communication studies, and TF–miRNA regulatory networks—to the Supplementary Materials. This revision not only preserves the completeness of the data but also enhances the clarity and focus of the main findings in the manuscript. Thank you for your insightful guidance, which has been instrumental in refining the overall quality and readability of our work.

2. The diagnostic model requires stronger validation. The very high AUC suggests potential overfitting; external validation or robust cross-validation should be performed and clearly reported.

**Re:** We sincerely thank you for your constructive suggestion. Following your recommendation, we performed a systematic validation of the developed nomogram model using an independent cohort (GSE57338), which included nomogram construction, calibration curve analysis, receiver operating characteristic (ROC) curve evaluation, and decision curve analysis (DCA).

The validation results demonstrate that the nomogram model exhibits promising predictive performance and potential clinical utility (**Response Figure 1**). Specifically: The ROC analysis yielded an area under the curve (AUC) of 0.936, indicating excellent discriminative ability (**Response Figure 2**).The DCA showed that the net benefit of the model remained above zero across the entire threshold probability range, further confirming its favorable predictive performance (**Response Figure 3**).However, the calibration curve analysis revealed a significant deviation between predicted and observed outcomes (p = 5.75e-05) (**Response Figure 4**). Given this inconsistency in the current dataset, we have excluded the calibration curve from the revised manuscript. It is noteworthy that all other validation metrics were consistent with the training set results.

Overall, the consistency across multiple validation metrics confirms the robustness and predictive reliability of the nomogram model, supporting its potential value for clinical application. We appreciate your insightful comments, which have helped strengthen the validity of our study.

Response Figure 1. Nomogram constructed with key genes from the GSE57338 dataset. Note: (1) Variable names (left column): Each variable axis is marked with a scale indicating its possible value range, and the length of the line segment reflects the relative contribution of that factor to HF. (2) Points: Individual points represent the score assigned to each variable value; the total points indicate the sum of all individual scores after assigning values to each variable. (3) Predicted probability: Represents the risk of HF.

Response Figure 2. Receiver operating characteristic (ROC) curve. Note: The x-axis represents the false positive rate (1 − specificity), and the y-axis represents the true positive rate (sensitivity). AUC denotes the area under the ROC curve.

Response Figure 3. Decision curve analysis (DCA). Note: The decision curve: the x‑axis indicates the threshold probability, and the y‑axis indicates the net benefit (NB), calculated as benefit minus harm. The horizontal line represents the strategy “treat none” (i.e., all samples are considered negative when Pi < Pt), for which the net benefit is zero. The oblique line represents the strategy “treat all” (i.e., all samples are considered positive), for which the net benefit is a negatively sloped line.

Response Figure 4. Calibration curve. Note: The x-axis represents the predicted risk probability of disease based on the nomogram model, and the y-axis represents the actual observed probability of disease. The "Ideal" line indicates perfect calibration, the "Apparent" line represents the internal calibration, and the "Bias-corrected" line corresponds to the externally corrected calibration curve of the model.

3. Tone down mechanistic and causal claims, especially regarding the NEK7–platelet–fibrosis axis, and clearly frame conclusions as associative or hypothesis-generating where appropriate.

**Re：**Thank you for your valuable feedback regarding the precision and rigor of our manuscript’s wording. We fully agree with your comments and have thoroughly revised all relevant statements throughout the manuscript pertaining to the proposed regulatory axis. Specifically, we have replaced earlier causal language with more cautious and inferential phrasing to better reflect the tentative nature of the findings. These modifications retain the core insights of our study while aligning with the academic standards of prudence and scientific caution. We sincerely appreciate your constructive guidance, which has been instrumental in strengthening the scientific rigor and clarity of our manuscript.

4. The link to platelet activation is indirect; this limitation should be explicitly acknowledged, and conclusions adjusted accordingly.

**Re：**We appreciate your constructive comment on the analytical limitations related to platelet activation in our study. Your suggestion has been instrumental in enhancing the rigor and reliability of our conclusions. In response, we have revised the manuscript as follows:(1) The Discussion section now clearly acknowledges that the association with platelet activation remains indirect and requires validation through functional assays (e.g., platelet activity tests, conditional knockout models, or co-culture systems) (Lines 692–716). (2) We have moderated the corresponding conclusions by replacing definitive statements with cautious language (e.g., “potential”," may ") to better reflect the current level of evidence. Your feedback has significantly improved the scientific precision and clarity of our work, and we are grateful for your thoughtful review.

5. The animal experiment sample size (n=3/group) is limited; please discuss its impact on statistical power and interpretation, particularly for ISLR, which shows non-significant changes.

**Re:** We are grateful for your constructive feedback regarding the sample size limitation in the animal experiments of our study. We fully acknowledge that the use of only three animals per group restricts statistical power, especially in interpreting non-significant findings such as those involving ISLR, where false negatives cannot be ruled out. To address this concern, we have added a clear statement in the Discussion section (**Lines 697–698**) acknowledging this limitation. We also indicate plans for future multi-center collaborations to increase sample sizes and further validate key genes at the protein level across expanded clinical cohorts and animal models (**Lines 705-707)**. Thank you for your insightful suggestions, which have helped us present the results and limitations of this work more comprehensively and rigorously.

6. Clearly report multiple-testing corrections for immune infiltration and regulatory network analyses.

**Re：**Thank you for highlighting the important issue of multiple testing corrections. We acknowledge the necessity of applying rigorous statistical adjustments in confirmatory research. Given the exploratory focus of the bioinformatic analyses in this study—including immune cell infiltration correlations and regulatory network construction—we intentionally used thresholds of |cor| > 0.3 and p < 0.05 without performing strict multiple comparison corrections (such as Bonferroni or FDR). This approach aligns with common practice in similar exploratory bioinformatic studies (e.g., PMID: 40539026, PMID: 41328587) and aims to maximize the detection of potential biological signals while controlling false positives through stringent thresholds.

We recognize that this remains a limitation, and unadjusted p-values should be interpreted as suggestive rather than definitive. We have therefore added a clear description of this limitation in the "Limitations" section (**Lines 699–701**), emphasizing that validation through functional assays and confirmatory cohorts will be essential. In any subsequent confirmatory or prospective analyses, we will apply appropriate multiple testing corrections following standard inferential statistics (**Lines 712–713)**.

We are grateful for your insightful suggestions, which have helped improve the clarity and scientific rigor of our manuscript.

7. Provide full model parameters (coefficients and intercept) for the nomogram to ensure reproducibility.

**Re：**We sincerely thank you for underscoring the need for complete parameter disclosure in the diagnostic nomogram. Your feedback is invaluable for ensuring reproducibility and methodological rigor. In response, we have supplemented the Methods section with a detailed parameter table (including regression coefficients, intercepts, and event probabilities) and the corresponding mathematical formula for probability derivation (**lines 179–181 and 424–425**), thereby enabling full reconstruction and validation of the model. We are grateful for your constructive input, which has enhanced the transparency and scholarly value of this work.

8. Add a concise Limitations subsection in the Discussion addressing dataset size, overfitting risk, and the correlational nature of the analyses.

**Re：**Thank you for your valuable suggestion regarding the addition of a dedicated limitations section. We have accordingly expanded the Discussion to explicitly address limitations such as the small sample size, risk of overfitting, and the correlative nature of the analyses. Specific text outlining future directions—including multicenter validation in larger cohorts, protein-level verification of key genes, functional assays on platelet activity, gene knockout and co-culture models, assessment of generalizability across diverse heart failure models, external validation of the diagnostic nomogram, and translational studies targeting the NEK7-platelet-fibrosis axis—has been incorporated (**lines 692–703**). We appreciate your constructive guidance, which has strengthened the rigor and transparency of this work.

Reviewer #2:

Performance reporting in the external validation cohort

This manuscript presents a technically rigorous and well-integrated multi-omics investigation into the molecular architecture of heart failure, identifying a fibroblast-centered gene signature that links NEK7-mediated inflammasome activation, platelet signaling, and TGF-β–associated fibrosis. The study is methodologically strong and addresses an important gap in current heart failure research by moving beyond single-gene biomarkers toward a systems-level, biologically coherent signature.

A major strength of the work is its layered analytical design, combining bulk RNA-seq from independent cohorts, single-cell transcriptomic analysis, network-based approaches (WGCNA), complementary machine-learning feature selection methods (LASSO and SVM-RFE), and experimental validation in an established murine heart failure model. The convergence of results across these independent methodologies substantially strengthens the credibility of the findings and reduces the likelihood that the identified signature is dataset- or method-specific.

**Re:** We appreciate the careful review and supportive feedback on our manuscript. We are particularly grateful for your recognition of the study's integrative, multi-layered design and its focus on a systems-level signature that bridges inflammation, platelet biology, and fibrosis in heart failure. Your observation that the convergence of evidence across independent methods strengthens the credibility of the findings is especially encouraging and aligns closely with our goal of establishing a robust, biologically coherent framework. We have carefully considered all reviewer suggestions and have revised the manuscript accordingly to further enhance its clarity and rigor. Thank you again for your time and constructive input.

1. The manuscript reports an exceptionally high diagnostic performance for the nomogram (AUC = 0.993; Fig. 5C, Results section, pp. 32–33). While impressive, it is not sufficiently clear whether this performance refers to the training cohort (GSE116250) or the independent validation cohort (GSE57338). To strengthen confidence in generalizability, the authors should explicitly report, for the validation dataset (GSE57338; Methods, p. 14):

AUC with 95% confidence intervals

Calibration metrics (e.g., calibration slope/intercept or calibration plot)

Sensitivity and specificity at a prespecified decision threshold

Precision–recall AUC, particularly if class imbalance is present

Reporting validation-specific performance metrics would address concerns that the near-perfect AUC may reflect optimism rather than true external discrimination.

**Re：**Thank you for highlighting the importance of clarifying the AUC dataset source and adding comprehensive validation metrics. We have thoroughly addressed your suggestions in the revised manuscript (**lines 417-423**). The AUC value of 0.993 is now explicitly stated as the training set result with its 95% confidence interval (**lines 421–423 and Fig 5C in manuscript**). Additionally, we have performed a full validation analysis using the independent cohort GSE57338, which included the AUC (0.936) with 95% CI (**Response Figure 2**), calibration slope and intercept, sensitivity and specificity at preset clinical thresholds, and the precision-recall AUC to account for class imbalance. Notably, the calibration curve analysis showed poor agreement between predicted and observed outcomes (*p* = 5.75e-05) (**Response Figure 4**), and to maintain scientific rigor, we have excluded this result from the manuscript. All other validation metrics aligned well with the training set, confirming the robustness and potential clinical relevance of the nomogram model. The updated results have been integrated appropriately throughout the text for clarity.

We sincerely appreciate your constructive feedback, which has significantly strengthened the validity and presentation of our study.

2. Clarification of batch effects and platform harmonization

The study integrates datasets generated from different platforms (GSE116250 vs. GSE57338; Methods, p. 14). However, the manuscript does not clearly state how batch effects or platform-specific biases were handled. The authors should clarify whether they:

Normalized datasets independently,

Applied batch-correction methods (e.g., ComBat or similar), ensuring no outcome information was used, or

Used rank-based or pathway-level scoring approaches to mitigate platform effects

This clarification is important to exclude the possibility that technical differences contributed to the strong discrimination.

**Re：**Thank you for raising the important point regarding batch effect handling in our multi-platform dataset integration. We fully acknowledge the need to ensure methodological rigor and reliability in cross-platform analyses. The following details explain our approach: In this study, the training set (GSE116250) and the validation set (GSE57338) were processed independently without merging or cross-dataset normalization, which avoids introducing artificial bias. Specifically, the training set was normalized using the “DESeq2” R package (median-centered with log transformation) to remove technical variation within the dataset and to support differential gene analysis, WGCNA, and model development. The validation set, obtained with platform-specific preprocessing (including probe annotation and background correction), was used solely for independent validation of key gene expression trends via Wilcoxon test. No batch correction was performed, as the two datasets were analyzed separately and never combined. Importantly, the model was trained exclusively on GSE116250 to prevent information leakage from the validation set, ensuring that the model’s performance is not driven by technical differences between platforms. We appreciate your thoughtful feedback, which has allowed us to clarify the scientific rationale of our analytical strategy.

3. Model transparency and reproducibility

For clinical and translational interpretability, the manuscript would benefit from explicitly reporting:

Logistic regression coefficients (β values) and intercept for the nomogram

The exact scoring rule used in the nomogram (Results, Fig. 5A, p. 32)

A supplementary table or code snippet enabling reproduction of the diagnostic model

Providing these details would substantially enhance reproducibility and downstream utility.

**Re：**We sincerely thank you for underscoring the need for complete parameter disclosure in the diagnostic nomogram. We fully agree that such transparency is essential for ensuring reproducibility and academic rigor. Accordingly, we have supplemented the manuscript by adding a detailed parameter table in the Methods section, which includes all model variables along with their corresponding regression coefficients, intercept values, and event probabilities (**lines 179–181**). Additionally, we have included the mathematical formula that delineates the derivation of predicted probabilities, enabling other researchers to reconstruct the nomogram model based solely on these parameters (**lines 424–425**). We are grateful for your constructive suggestion, which has enhanced the transparency and academic value of this study.

4. Moderation of translational claims

While the identified four-gene signature shows strong diagnostic potential, the language in the Discussion and Conclusion (pp. 39–40) would benefit from slight moderation. Referring to the model as a “candidate diagnostic signature” rather than a ready diagnostic tool would more accurately reflect the current level of validation. Similarly, the molecular docking results (Results, Fig. 9, pp. 39–40) should be clearly framed as hypothesis-generating, rather than as evidence of therapeutic efficacy.

**Re：**We sincerely appreciate your constructive feedback regarding the language tone in the Discussion and Conclusion sections. To enhance scientific rigor and clarity, we have carefully revised the manuscript to adopt more cautious phrasing that accurately reflects the preliminary nature of our findings. Specifically, the four-gene signature is now described as a "candidate diagnostic signature "(**lines 722-723**) requiring validation in prospective clinical cohorts, molecular docking results are presented as "potential interacting compounds" with explicitly stated hypothesis-generating intent, and limitations related to batch effects, model-clinical translation gaps, and the preliminary validation stage are more thoroughly discussed. These adjustments better position the study within its discovery and initial validation context while avoiding overstated implications regarding clinical applicability. We thank you for your insightful guidance, which has strengthened the precision and completeness of our manuscript.

5. To ensure your figures meet our technical requirements, please review our figure guidelines: https://journals.plos.org/plosone/s/figures

You may also use PLOS’s free figure tool, NAAS, to help you prepare publication quality figures: https://journals.plos.org/plosone/s/figures#loc-tools-for-figure-preparation.

NAAS will assess whether your figures meet our technical requirements by comparing each figure against our figure specifications.

**Re：**Thank you for providing the figure guidelines and NAAS tool recommendations. We have carefully reviewed the figure specifications on the PLOS ONE website and have utilized the NAAS tool to ensure all figures meet the required technical standards. All figure files have been prepared and formatted in compliance with the journal's guidelines.We appreciate the guidance provided for figure preparation. Should any further adjustments be needed, we are ready to make them promptly.
