# Supplementary material for: Integrated NEK7-inflammasome and platelet transcriptomic signature generates mechanistic hypotheses in heart failure
Source: PLoS One. 2026 Jun 26;21(6):e0352124. doi: 10.1371/journal.pone.0352124 (PMC13308797; doi:10.1371/journal.pone.0352124)
Supplement: Attachment — Submitted filename: response to the reviewers.docx [file pone.0352124.s025.docx]

Point-by-point response to the reviewer comments.

Dear Reviewers,

Thank you for your thoughtful suggestions and insights, which have benefited from the manuscript. I am looking forward to working with you to move this manuscript closer to publication in PLOS ONE.

The manuscript has been rechecked and the necessary changes have been made in accordance with your suggestions. The responses to all comments have been prepared and attached below. We have tried our best to solve the problems you proposed, and we hope that the revised manuscript is now suitable for publication in the journal PLOS ONE. If you have any questions remained about this paper, please feel free to contact us.

Comments to the Author

1. If the authors have adequately addressed your comments raised in a previous round of review and you feel that this manuscript is now acceptable for publication, you may indicate that here to bypass the “Comments to the Author” section, enter your conflict of interest statement in the “Confidential to Editor” section, and submit your "Accept" recommendation.

Reviewer #1: All comments have been addressed

Reviewer #3: All comments have been addressed

**Re:** We sincerely appreciate your acknowledgment that all our revisions have adequately addressed the comments raised in the previous round. We are deeply honored and encouraged by your recognition of our point-by-point revisions and responses. Your rigorous, meticulous, and professional guidance throughout the review process has substantially enhanced the scientific rigor and academic value of our study. We will strictly adhere to the journal's requirements for final manuscript preparation and formatting. Once again, we extend our most sincere gratitude for your invaluable review.

2. Is the manuscript technically sound, and do the data support the conclusions?

The manuscript must describe a technically sound piece of scientific research with data that supports the conclusions. Experiments must have been conducted rigorously, with appropriate controls, replication, and sample sizes. The conclusions must be drawn appropriately based on the data presented.

Reviewer #1: Partly

Reviewer #3: Yes

**Re：**We sincerely thank you for your recognition of the technical soundness of our study and the supportive nature of our data for the conclusions. Your confirmation that our experimental design includes appropriate controls, replication, and adequate sample sizes, and that our conclusions are rigorously and appropriately drawn from the presented data, serves as tremendous encouragement and validation of our research efforts. Throughout this study and revision process, we have consistently adhered to the principles of technical rigor, data reliability, and objective conclusion-drawing, strictly following academic norms to ensure the scientific credibility of our work. We once again express our heartfelt gratitude for your thorough, professional, and meticulous guidance throughout the review process.

3. Has the statistical analysis been performed appropriately and rigorously?

Reviewer #1: Yes

Reviewer #3: Yes

**Re：**We are genuinely grateful for your full endorsement of the statistical analyses performed in this study. Your confirmation that appropriate and rigorous statistical methods have been employed is a strong affirmation of the scientific rigor of our methodological approach, and we are truly encouraged by your positive evaluation. We have always placed high priority on statistical rigor and analytical precision, strictly adhering to academic standards in all statistical tests to ensure the reliability and credibility of our findings. We wish to express our sincere gratitude for your professional, meticulous, and rigorous guidance throughout the review process..

4. Have the authors made all data underlying the findings in their manuscript fully available?

The PLOS Data policy requires authors to make all data underlying the findings described in their manuscript fully available without restriction, with rare exception (please refer to the Data Availability Statement in the manuscript PDF file). The data should be provided as part of the manuscript or its supporting information, or deposited to a public repository. For example, in addition to summary statistics, the data points behind means, medians and variance measures should be available. If there are restrictions on publicly sharing data—e.g. participant privacy or use of data from a third party—those must be specified.

Reviewer #1: Yes

Reviewer #3: Yes

**Re：**We greatly appreciate your recognition of our data availability practices. Your confirmation that we have fully complied with the PLOS Data Policy requirements by making all supporting data publicly available without restriction, depositing data in public repositories, and clearly specifying any reasonable limitations on data sharing, affirms our commitment to research transparency and reproducibility. Your positive assessment serves as tremendous encouragement for our dedication to strictly adhering to academic norms and journal requirements. We are truly thankful for your thorough and meticulous guidance throughout the review process.

5. Is the manuscript presented in an intelligible fashion and written in standard English?

PLOS ONE does not copyedit accepted manuscripts, so the language in submitted articles must be clear, correct, and unambiguous. Any typographical or grammatical errors should be corrected at revision, so please note any specific errors here.

Reviewer #1: Yes

Reviewer #3: Yes

**Re：**We sincerely appreciate your confirmation that our manuscript is presented in an intelligible fashion and written in standard English. We have always placed high priority on linguistic clarity, readability, and formatting precision. We have carefully proofread the entire manuscript to correct any typographical or grammatical errors, ensuring full compliance with PLOS ONE requirements. Your affirmation is tremendous encouragement for our efforts in manuscript preparation. We warmly thank you for your rigorous and professional guidance.

Reviewer #1: This manuscript presents a comprehensive multi-omics investigation into a NEK7-associated fibro-inflammatory gene signature in heart failure. The integrative analytical framework—combining bulk RNA-seq, single-cell analysis, machine learning, and experimental validation—is methodologically solid and addresses a biologically relevant question. The convergence of findings across independent datasets strengthens the credibility of the identified four-gene signature.

However, several issues warrant attention. The exceptionally high training AUC suggests potential overfitting, and although external validation was performed, the reported poor calibration in the validation cohort raises concerns regarding model generalizability. This should be transparently reported and discussed rather than minimized. The animal validation is limited by small sample size (n=3/group) and lack of protein-level confirmation, reducing biological robustness. In addition, immune infiltration and regulatory network analyses were conducted without formal multiple-testing correction, which increases the risk of false-positive findings. Mechanistic interpretations of the NEK7–platelet–fibrosis axis remain largely correlational and should be framed cautiously.

No concerns regarding dual publication, research ethics, or competing interests are apparent based on the materials provided. Overall, the study is scientifically interesting and potentially publishable after minor-to-moderate revision focusing on transparency, calibration reporting, and cautious interpretation of translational implications.

**Re：**We sincerely thank you for your highly positive evaluation of the scientific value, methodological design, and integrative analytical framework of our study. Your recognition of our systematic approach combining bulk RNA-seq, single-cell sequencing, machine learning, and experimental validation, as well as the biological relevance of our research question and the critical value of independent dataset validation, is tremendously encouraging. We are also grateful for your confirmation that our study raises no concerns regarding duplicate publication, research ethics, or competing interests. Your rigorous identification of four core issues has been instrumental in guiding our efforts to enhance model generalizability, experimental robustness, statistical rigor, and mechanistic interpretability. We fully acknowledge and accept these valuable comments and have systematically addressed each one as detailed below. We remain sincerely grateful for your comprehensive evaluation and the invaluable insights that have guided our revisions.

**In response to your concerns about potential model overfitting,** including the high training AUC, the suboptimal calibration in external validation, and the need for more transparent discussion of these limitations, we have taken significant steps to strengthen our model validation. Specifically, we incorporated an additional independent dataset, GSE5406, to perform further external validation of our nomogram. The results from this new validation support the reliability of our model. These additions and corresponding discussions are detailed in the revised manuscript (**Lines 114-116, 201-204, 443-452)**.

**Regarding the limitations of our animal experiments,** particularly the small sample size and the absence of protein-level validation, we fully concur with your assessment and sincerely appreciate your understanding. As we explain in the revised manuscript, the Ang II-induced heart failure model used in this study involves a prolonged modeling period. Unfortunately, the remaining cardiac tissue from this batch was allocated to another transcriptomic study, leaving no residual samples for Western Blot or other protein-level validation. We fully recognize that expanding the sample size and performing protein-level confirmation are essential to further substantiate our conclusions. However, given the impending deadline for my doctoral graduation, we are unable to complete a new round of animal modeling and tissue collection in the short term. This constitutes an objective limitation of the current study, which we have transparently acknowledged in the revised manuscript **(Lines 779-785**). We have also outlined future directions, including multi-center clinical collaboration to expand sample size, and systematic validation of core gene expression and underlying mechanisms at the protein level in diverse heart failure animal models, to enhance the robustness and generalizability of our findings. We thank you again for your valuable comments, which are instrumental in guiding our critical self-reflection and clarifying future research priorities.

**With respect to the statistical rigor of our bioinformatics analyses,** we agree that the absence of multiple testing correction in the immune infiltration and regulatory network analyses could increase the risk of false-positive findings. To address this, we have now re-performed these analyses strictly applying the Benjamini–Hochberg (BH) multiple testing correction method. This approach enables us to rigorously control for false positives from a statistical standpoint, thereby ensuring the scientific validity of our bioinformatics results. Detailed revisions are provided in **Lines 230–235, 501-514.**

**Finally, we have carefully reconsidered our interpretation of the NEK7–platelet–fibrosis axis,** as you rightly pointed out that the current evidence is largely correlational and requires more cautious framing. We have now refined the mechanistic discussion to more objectively distinguish between correlation and potential causality, and to clearly delineate the boundaries of our mechanistic inferences. These revisions substantially enhance the rigor and credibility of our mechanistic interpretations, as detailed in **Lines 638-650**. Once again, we extend our heartfelt gratitude for your professional guidance and invaluable feedback, which have led to a qualitative leap in the overall quality of our manuscript.

Reviewer #3: Review of Manuscript PONE-D-25-65980: "Integrated NEK7-inflammasome and platelet signature delineates a diagnostic model in heart failure"

Overall Assessment:

This is a well-conceived and comprehensive study that integrates multi-omics data and machine learning to identify a novel four-gene signature (TIMP2, COL16A1, MDK, ISLR) associated with NEK7-mediated inflammation and platelet activation in heart failure (HF). The work is analytically robust, employs appropriate and sophisticated bioinformatic methodologies, and presents a compelling narrative linking fibrosis, inflammation, and thrombosis in HF pathogenesis. The construction of a high-accuracy diagnostic nomogram (AUC=0.993) is a significant finding. However, several major and minor concerns must be addressed to strengthen the manuscript's validity, clarity, and translational impact before publication.

Major Concerns

1. Validation and Overfitting Risk:

The reported diagnostic accuracy (AUC 0.993) is exceptionally high. While internal validation is performed on GSE57338, this is still a retrospective, public dataset. There is a significant risk of model overfitting to the specific cohorts used (GSE116250 for training/feature selection). The authors must:

Explicitly discuss the potential for overfitting in the Limitations section.

Provide more robust validation. Ideally, this should involve an independent, prospective clinical cohort with blood or tissue samples to test the signature's diagnostic performance in a real-world setting. If this is not feasible within the current study, the limitations section must strongly emphasize this as a critical next step and temper claims of immediate clinical translatability.

Perform cross-validation (e.g., leave-one-out or repeated k-fold) during the model-building process in the training set and report those metrics.

**Re：**We sincerely thank you for your rigorous and constructive comments, which have critically pointed out the key issue regarding the exceptionally high AUC value of 0.993 in our diagnostic model and the associated significant risk of overfitting. In response to your essential requests for additional model validation, explicit discussion of overfitting risks, and toning down claims related to clinical translation, we have taken the following actions:

(1) **Additional Validation:** We have incorporated a new independent dataset, GSE5406, to validate the nomogram model. The results demonstrate the reliability of our findings. For details, please refer to **lines 114–116, 201–204,** **and 443–452** in the revised manuscript.

**(2) Discussion of Overfitting and Future Validation:** In the revised manuscript, we have directly, thoroughly, and objectively analyzed the risk of overfitting and the underlying reasons for the high AUC in the discussion section. We have also outlined our plans to rigorously evaluate the diagnostic model's performance in future large-scale prospective cohorts through external validation and cross-validation. This will aim to enhance its clinical robustness and generalizability, and to assess its value in differential diagnosis, risk stratification, and prognosis prediction **(lines 786–795 in the revised manuscript**).

**(3) Refined Claims:** We have strictly moderated and standardized the language regarding clinical translation and diagnostic applicability. The scientific research value and practical application boundaries of the model are now more appropriately defined, thereby enhancing the rigor and credibility of the study.

Once again, we are deeply grateful for your valuable insights and professional guidance.

2. Functional Link Between Signature and Platelet Activation:

The core hypothesis links the gene signature to platelet activation. However, the evidence for this link is primarily bioinformatic (correlation with a platelet gene set). There is a lack of direct experimental or causal evidence connecting the upregulation of TIMP2, COL16A1, MDK, or ISLR in cardiac fibroblasts to platelet hyperactivity or its consequences in HF.

The authors should either:

Acknowledge this as a key limitation and frame the signature as "associated with a platelet activation gene program" rather than implying a direct mechanistic role in platelet biology.

Or, propose and (if possible, preliminarily) test a more direct connection. For example, could conditioned media from fibroblasts overexpressing these genes activate platelets in vitro? Are protein levels of these markers correlated with plasma markers of platelet activation (e.g., soluble P-selectin, PF4) in available patient serum/plasma data?

**Re：**We greatly appreciate your insightful and rigorous critique, which has astutely identified a pivotal limitation in our work: the core hypothesis linking the four-gene signature (TIMP2, COL16A1, MDK, ISLR) to platelet activation is currently supported only by bioinformatic correlation with a platelet activation gene set, without direct experimental or causal evidence to connect the upregulation of these genes in cardiac fibroblasts to platelet hyperactivation or its pathological consequences in heart failure. Your expert guidance is invaluable for addressing this mechanistic gap and strengthening the scientific foundation of our study. In accordance with your recommendations to either refine the claims and emphasize this limitation or to provide direct experimental validation, we have taken the following actions: (1) We have carefully revised the description of the mechanistic hypothesis throughout the manuscript to clearly distinguish correlational findings from direct causal evidence, as reflected in **lines 92-94 o**f the revised manuscript. (2) In the Discussion section, we have explicitly acknowledged that the current evidence is primarily derived from bioinformatic co-expression analysis and lacks direct experimental verification. We have also outlined a concrete plan for future investigations—including multicenter collaborations to expand sample sizes, protein-level validation of the core genes and proposed mechanisms in diverse heart failure animal models, and functional assays such as platelet function tests and gene knockout models to elucidate the role of platelet activation—as detailed in **lines 801-810** of the original manuscript. We are once again deeply grateful for your thoughtful guidance and meticulous review, which have substantially improved the clarity and rigor of our study.

3. Animal Model Validation:

The in vivo validation is limited to mRNA expression in an Ang-II model (n=3/group). The sample size is very small, limiting statistical power. More importantly, protein-level validation (western blot, immunohistochemistry) of the key genes in the murine hearts and, if possible, in human HF tissue samples is crucial to confirm the bioinformatic predictions.

The non-significant result for Islr (P=0.5691) is appropriately discussed but warrants deeper investigation. Is this due to species differences, timing, or post-transcriptional regulation? IHC could clarify if ISLR protein is localized differently or expressed in a relevant cell type.

**Re：**We are truly grateful for your thorough and constructive critique, which has insightfully highlighted several critical weaknesses in our in vivo validation experiments. Specifically, you pointed out that our reliance on mRNA expression data from an Ang-II induced heart failure model with only three mice per group severely limits statistical power; that the lack of protein-level validation and corroboration from human HF tissue samples undermines the confirmation of bioinformatic predictions; and that our analysis of the non-significant result for Islr$P=0.5691$ remains superficial, failing to explore potential underlying mechanisms such as species differences, timing of tissue collection, post-transcriptional regulation, or the specific cellular localization and expression patterns of ISLR protein. Your expert guidance is immensely valuable for strengthening the methodological rigor and scientific credibility of our work. In response, we have systematically addressed these concerns in the revised manuscript: we have explicitly acknowledged these limitations in the Discussion section, provided a more in-depth analysis of the possible factors contributing to the Islr expression result, and clearly outlined a comprehensive plan for future investigations—including expanding animal sample sizes, performing Western blot and immunohistochemistry for protein-level validation, conducting validation studies in human HF tissue samples, and elucidating the tissue localization and dominant cell types expressing ISLR protein—as detailed in **lines 779-785, 801-810** of the revised manuscript. These revisions have substantially enhanced the completeness and rigor of our study. We sincerely thank you once again for your invaluable review and professional guidance.

4. Diagnostic Utility and Clinical Context:

The discussion comparing the nomogram's AUC (0.993) to BNP/NT-proBNP (AUC ~0.95-0.99 for HF vs. healthy) requires more nuance. The critical clinical challenge is not distinguishing severe HF from healthy controls, but early detection, differential diagnosis (e.g., HFpEF vs. pulmonary disease), or prognostication. The authors should:

Test or discuss how their signature performs in these more challenging, clinically relevant scenarios using available data (e.g., stratifying within HF subgroups).

Clarify the intended clinical specimen for this test. Is it intended for cardiac tissue (limiting utility) or is there evidence these genes are detectable in peripheral blood (e.g., in circulating fibroblasts, extracellular vesicles, or as soluble proteins)?

**Re：**We truly appreciate your insightful comments, which have highlighted critical gaps in our analysis of diagnostic utility and clinical context. In response, we have systematically addressed these concerns in the revised manuscript by explicitly acknowledging these limitations in the Discussion section, providing a candid assessment of the current analytical shortcomings and practical constraints on clinical application. Furthermore, we have substantially expanded and refined our future research directions, clearly outlining plans to systematically validate the signature's incremental value across multiple clinical scenarios in prospective cohorts, with a particular focus on evaluating the feasibility of blood-based detection (including plasma proteins and exosomal mRNA) to facilitate its translation toward non-invasive liquid biopsy and broader clinical implementation—as detailed in **lines 787-795, 810-819** of the revised manuscript. These revisions have significantly enhanced the completeness and translational clarity of our study. We are deeply grateful once again for your thorough review and invaluable guidance.

Minor Concerns

5. Methods Clarity:

WGCNA Parameters: Please specify the network type (signed/unsigned) chosen for WGCNA and the rationale.

Single-cell Analysis: For Seurat analysis, state the normalization method used (`LogNormalize` or `SCTransform`). The criteria for selecting 30 PCs should be better justified (e.g., elbow plot shown in Suppl.).

Molecular Docking: Briefly describe the preparation steps for the protein structures (e.g., missing loops, protonation state). The threshold Vina score ≤ -5.0 kcal/mol is acceptable but consider that stronger binders typically have scores < -7.0 kcal/mol; comment on the predicted binding affinities.

**Re：**We sincerely thank you for your meticulous and professional suggestions regarding the methodological details of our study. Your comments—specifically requesting clarification of unspecified analytical parameters for WGCNA, Seurat single-cell analysis, and molecular docking, as well as strengthening the rationale for parameter selection—are essential for enhancing the methodological transparency, rigor, and reproducibility of our work. In accordance with your guidance, we have carefully reviewed and comprehensively supplemented all previously unspecified parameters in the Methods section, provided detailed scientific justifications for each parameter choice, and elaborated on the key analytical steps. These revisions are presented in **lines** **142-150, 248-253, and 259-265** of the revised manuscript. We are once again deeply grateful for your thorough review and invaluable guidance.

6. Results Presentation:

Figure 2G: The heatmap shows module-trait correlations. Please ensure the color scale is clearly labeled, and the statistical significance (p-values) for the key turquoise module correlation with both traits is indicated in the figure or legend.

Immune Infiltration: The positive correlation between NEK7 and CD56dim NK cells is interesting. Could the authors speculate briefly in the discussion on the potential role of this immune subset in the NEK7-inflammasome-platelet axis?

CellChat Analysis: The finding of MIF-(CD74+CD44) as a key ligand-receptor pair is notable. Is MIF expression part of the identified gene signature or upregulated in HF fibroblasts? A brief comment would connect this communication finding back to the core signature.

**Re：**We are sincerely grateful for your precise, professional, and highly constructive comments regarding the refinement of our results presentation and the extension of core findings through associative analysis. Your suggestions have been invaluable in enhancing the rigor and logical coherence of our study.

**(1) Regarding Figure 2G,** you pointed out that the module-trait correlation heatmap lacks a clearly labeled color scale and does not display the statistical significance (*p*-values) for the key turquoise module's correlation with both traits. In response, we have revised Figure 2G to include a fully labeled color scale and have added the corresponding p-values to the figure or legend, thereby improving the clarity and rigor of our results presentation.

**(2) With regard to your query on the immune infiltration analysis**, you noted the interesting positive correlation between NEK7 and CD56dim NK cells and suggested speculating on the potential role of this immune subset within the NEK7-inflammasome-platelet axis. In response, we have added a brief discussion in the revised manuscript elaborating on the possible functional involvement of CD56dim NK cells in this regulatory axis, thereby strengthening the logical connection among these key findings—as detailed in **lines 720-745** of the revised manuscript.

**(3) Turning to your comment on the CellChat analysis**, you highlighted that the MIF-(CD74+CD44) ligand-receptor pair is notable and questioned whether MIF expression is part of the identified gene signature or upregulated in HF fibroblasts, which would connect this intercellular communication finding back to our core signature. In response, we have supplemented our analysis by characterizing MIF expression patterns and clarifying its relationship with the four-gene signature, thereby more fully leveraging the value of our existing data—as detailed in **lines 703-719** of the revised manuscript.

We once again extend our deepest gratitude for your rigorous review and invaluable guidance, which have substantially enhanced the systematic presentation and logical coherence of our study's results.

7. Discussion & Limitations:

The limitations section is good but should be expanded as per the major points above (overfitting, lack of functional platelet link, preliminary animal data).

The therapeutic implications (β-Heparin/MDK, WZ4002/NEK7) are speculative but interesting. Acknowledge that molecular docking is a predictive starting point requiring extensive experimental validation.

**Re：**We sincerely appreciate your positive acknowledgment of the existing limitations section in our study, as well as your insightful suggestions for its precise expansion and refinement. Your guidance is of paramount importance for further enhancing the rigor of our discussion and objectively delineating the boundaries of our research.

In accordance with your request to integrate the core issues raised in your previous comments, we have systematically supplemented the Limitations section to elaborate on the potential impact of each critical issue—including overfitting risk, lack of functional platelet link, and preliminary animal data—on the reliability, generalizability, and mechanistic interpretability of our conclusions, thereby presenting an objective assessment of the current constraints and shortcomings of our study（**lines 774-798**）.

Regarding your concern that the therapeutic implications of β-Heparin/MDK and WZ4002/NEK7 are speculative, we have explicitly addressed this in the Discussion section by clearly stating that molecular docking serves merely as a predictive starting point, and we have emphasized that the proposed therapeutic potential requires extensive subsequent in vitro and in vivo experimental validation. These revisions help to rigorously define the boundaries of our conclusions and avoid overinterpretation, as detailed in lines **795-798** of the revised manuscript.

We are once again deeply grateful for your meticulous review and professional guidance, which have significantly contributed to making our discussion more objective, comprehensive, and rigorous.

8. Writing and Formatting:

Minor grammatical errors and typos are present (e.g., "offers" should be "offer" in Conclusion, Page 10 line 43; "data abases" Page 21). A thorough proofread is needed.

Ensure all figure and table citations in the text are correct (e.g., reference to S1-S4 Figs., S1-S19 Tables).

The Data Availability Statement is slightly confusing and repetitive. It mentions Figshare deposition "under moderation" and a Mendeley Data DOI. Please consolidate to one definitive, publicly accessible repository and provide the final, working DOI/accession.

**Re：**We sincerely thank you for your meticulous and rigorous guidance regarding the writing and formatting details of our manuscript. We fully acknowledge that these issues—including minor grammatical errors, inconsistent figure/table citations, and a confusing Data Availability Statement—can be readily resolved through careful proofreading, uniform formatting, and concise revision, and we have strictly implemented all your suggestions accordingly.

We have conducted a thorough, line-by-line proofread of the entire manuscript to correct all grammatical errors and typos. Additionally, we have carefully verified every citation of supplementary figures S1-S4 Figs and tables (S1-S19 Tables) throughout the text to ensure accurate numbering and formatting, with no missing or incorrect references. Furthermore, we have streamlined the Data Availability Statement by consolidating the deposition information into a single, definitive, publicly accessible repository, providing the final working DOI/accession number and removing all redundant or ambiguous phrasing, in full compliance with journal requirements. These revisions are detailed in **lines 860-868** of the revised manuscript.

We are once again deeply grateful for your thorough review and professional guidance, which have substantially enhanced the standardization and readability of our manuscript.
