# Supplementary material for: Integrated NEK7-inflammasome and platelet transcriptomic signature generates mechanistic hypotheses in heart failure
Source: PLoS One. 2026 Jun 26;21(6):e0352124. doi: 10.1371/journal.pone.0352124 (PMC13308797; doi:10.1371/journal.pone.0352124)
Supplement: Attachment — Submitted filename: response to the Editor.docx [file pone.0352124.s026.docx]

Point-by-point response to the reviewer comments.

Dear Editor and Reviewers

Thank you for your thoughtful and constructive comments. We have carefully addressed all concerns point by point. The revised manuscript has been prepared with all changes clearly marked. Below are our detailed responses.

**Comment 1:** The additional validation dataset, GSE5406, is not adequately justified, and the authors do not clearly explain whether it is comparable to the model-development dataset. The risk of overfitting also remains because the authors have not provided sufficient additional validation analyses, such as repeated cross-validation, bootstrap optimism correction, or detailed calibration assessment across datasets.

**Re:** We thank the editor for this critical comment. We have made the following additions:

1.1 **GSE5406 comparability**

(1) In **Methods (Source of data, lines 121–131)**, we added baseline characteristic comparisons (species, tissue source, sample grouping, platform) across GSE5406, GSE116250, and GSE57338, along with batch effect evaluation using the sva package. **No significant batch bias was observed after standardization.**
(2) A new **Table 1** summarizes the detailed information of the three datasets.
(3) As acknowledged in **Limitations (point 2, lines 795–799)**, potential batch effects cannot be completely excluded, and further validation in prospective, multi-center cohorts is necessary.

**1.2 Overfitting risk**

(1) In **Methods (Nomogram construction and evaluation, lines 203–226)**, we added: ①L2 regularization (ridge regression) for nomogram construction; ②10 iterations of 10-fold repeated cross-validation; ③1000-times bootstrap optimism correction; and ④cross-dataset calibration assessment.
(2) In **Results (Candidate nomogram predictive performance validation, lines 457–501)**, we reported: mean AUC = 0.989 from repeated cross-validation; optimism-corrected AUC = 0.985; Hosmer–Lemeshow tests *P*=0.928 (GSE116250), 0.179 (GSE57338), and 0.836 (GSE5406); and calibration curves closely approximating the ideal diagonal.
(3) New **Table 1(lines 132–134)** demonstrates the comparability of the three datasets (platform, tissue, sample grouping, sequencing type).
(4) **Fig 5** has been redrawn with additional annotations for calibration and stability validation.
(5) As stated in **Limitations (point 3, lines 799–804)**, due to the limited sample size of the discovery dataset, some degree of overfitting risk remains, and external validation in larger independent cohorts is required.

**Comment 2:** The animal validation also remains weak because it is based on n=3 animals per group and mRNA-level qPCR only. The authors did not increase the sample size or provide protein-level validation. Therefore, the animal data should be described as preliminary support rather than confirmatory validation.

**Re:** We are grateful to the Academic Editor for this thoughtful comment. We fully agree and have revised the manuscript accordingly:

(1) In **Methods (RT-qPCR, lines 376–377)**, we added: “This section represents a preliminary mRNA expression detection with a limited animal sample size (n=3 per group).”
(2) In **Results (Key genes expression, lines 674–681)**, we changed statements from “confirming the pivotal roles” and “further substantiate the conclusions” to “**preliminary detection** of mRNA expression” and “providing **preliminary support**.”
(3) In **Table 3 and Fig 12 legends (lines 664–673)**, we added: “Note: Animal experiments had a sample size of n=3 per group, and only mRNA-level detection was performed; the results are **preliminary findings**.”
(4) In **Limitations (point 4, lines 804–810)**, we explicitly state: “the animal data should be interpreted as **preliminary support** rather than confirmatory validation.”
(5) In **Conclusions (lines 857–859)**, we revised to: “Animal experimental results provide **preliminary in vivo validation support** for the above mechanisms and biomarker conclusions.”

**Comment 3: Reframe as hypothesis-generating study (not a validated diagnostic tool)** (- In addition, the proposed link between the four-gene signature and platelet activation remains mainly correlative. The manuscript should avoid causal or mechanistic claims unless supported by direct functional evidence.

Because the manuscript presents a diagnostic model, the authors must also address the requirements for utility, validity, and availability. At present, the model’s clinical utility is not established because it is based on cardiac tissue datasets and has not been validated in prospective cohorts, blood-based samples, or clinically relevant diagnostic settings.

Therefore, before further consideration, the authors should either provide stronger validation and additional analyses to support the diagnostic-model claim, or substantially reframe the manuscript as a hypothesis-generating transcriptomic study rather than a validated diagnostic tool.)

**Re:** We appreciate the Academic Editor’s constructive guidance on reframing the manuscript. We have substantially reframed the entire manuscript as a **hypothesis-generating transcriptomic analysis**. The following specific revisions have been made:

| Section | Changes | Lines |
| --- | --- | --- |
| **Title** | Changed to: “Integrated NEK7-inflammasome and platelet transcriptomic signature generates mechanistic hypotheses in heart failure” (removing “diagnostic model”) | **1–3** |
| **Abstract** | Revised aim to “explore transcriptomic associations and generate pathogenic hypotheses”; changed “diagnostic nomogram” to “preliminary discrimination model with **no clinical diagnostic value**” | **17–46** |
| **Keywords** | Removed “biomarkers” and “diagnostic models”; added “hypothesis-generating” and “transcriptomic signature” | **48–49** |
| **Introduction** | Softened claims about diagnostic value; focused on mechanistic exploration and hypothesis generation | **71–104** |
| **Results** | Rewrote “Nomogram predictive performance validation” subsection; replaced “diagnostic nomogram” with “preliminary discrimination model” and stated “**no clinical diagnostic value**” | **456–501** |
| **Discussion** | **Deleted** the entire paragraph on clinical translation (including comparisons with BNP/NT-proBNP and therapeutic development prospects) | **Deleted** |
| **Limitations** | Added explicit statement: “This is a hypothesis-generating transcriptomic analysis; all mechanistic inferences require direct functional validation; the model is based on myocardial tissue without validation in blood or prospective cohorts, and therefore **has no established clinical value**” | **795–815** |
| **Conclusions** | Revised: removed all diagnostic claims; positioned as a hypothesis-generating framework for HF pathogenesis | **850–864** |

**Summary of changes highlighted in the manuscript**

All revisions are marked with track changes in the “Revised Manuscript with Track Changes” file. Line numbers refer to the clean revised manuscript file.

We believe the revised manuscript now fully meets the publication criteria of PLOS ONE. We sincerely thank the editor and reviewers for their constructive feedback, which has significantly improved our work.
