# Supplementary material for: Integrated NEK7-inflammasome and platelet transcriptomic signature generates mechanistic hypotheses in heart failure
Source: PLoS One. 2026 Jun 26;21(6):e0352124. doi: 10.1371/journal.pone.0352124 (PMC13308797; doi:10.1371/journal.pone.0352124)
Supplement: Peer review history

# Peer review: Decision Letter 0

**DOI:** 10.1371/journal.pone.0352124.r001

**Authors:** Xuebo Zhang

7 Jan 2026

PONE-D-25-65980

Integrated NEK7-inflammasome and platelet signature delineates a diagnostic model in heart failure

PLOS One

Dear Dr. Dai,

Thank you for submitting your manuscript to PLOS ONE. After careful consideration, we feel that it has merit but does not fully meet PLOS ONE’s publication criteria as it currently stands. Therefore, we invite you to submit a revised version of the manuscript that addresses the points raised during the review process.

Please submit your revised manuscript by Feb 21 2026 11:59PM. If you will need more time than this to complete your revisions, please reply to this message or contact the journal office at plosone@plos.org. When you're ready to submit your revision, log on to https://www.editorialmanager.com/pone/ and select the ’submissions Needing Revision' folder to locate your manuscript file.

Please include the following items when submitting your revised manuscript:

- A letter that responds to each point raised by the academic editor and reviewer(s). You should upload this letter as a separate file labeled 'Response to Reviewers'.
- A marked-up copy of your manuscript that highlights changes made to the original version. You should upload this as a separate file labeled 'Revised Manuscript with Track Changes'.
- An unmarked version of your revised paper without tracked changes. You should upload this as a separate file labeled 'Manuscript'.

If you would like to make changes to your financial disclosure, please include your updated statement in your cover letter. Guidelines for resubmitting your figure files are available below the reviewer comments at the end of this letter.

If applicable, we recommend that you deposit your laboratory protocols in protocols.io to enhance the reproducibility of your results. Protocols.io assigns your protocol its own identifier (DOI) so that it can be cited independently in the future. For instructions see: https://journals.plos.org/plosone/s/submission-guidelines#loc-laboratory-protocols. Additionally, PLOS ONE offers an option for publishing peer-reviewed Lab Protocol articles, which describe protocols hosted on protocols.io. Read more information on sharing protocols at https://plos.org/protocols?utm_medium=editorial-email&utm_source=authorletters&utm_campaign=protocols.

We look forward to receiving your revised manuscript.

Kind regards,

Xuebo Zhang, Ph.D.

Academic Editor

PLOS One

Journal Requirements:

When submitting your revision, we need you to address these additional requirements.

1. Please ensure that your manuscript meets PLOS ONE’s style requirements, including those for file naming. The PLOS ONE style templates can be found at

https://journals.plos.org/plosone/s/file?id=wjVg/PLOSOne_formatting_sample_main_body.pdf and

https://journals.plos.org/plosone/s/file?id=ba62/PLOSOne_formatting_sample_title_authors_affiliations.pdf

2. Please note that PLOS One has specific guidelines on code sharing for submissions in which author-generated code underpins the findings in the manuscript. In these cases, all author-generated code must be made available without restrictions upon publication of the work. Please review our guidelines at https://journals.plos.org/plosone/s/materials-and-software-sharing#loc-sharing-code and ensure that your code is shared in a way that follows best practice and facilitates reproducibility and reuse.

3. PLOS requires an ORCID iD for the corresponding author in Editorial Manager on papers submitted after December 6th, 2016. Please ensure that you have an ORCID iD and that it is validated in Editorial Manager. To do this, go to ‘Update my Information’ (in the upper left-hand corner of the main menu), and click on the Fetch/Validate link next to the ORCID field. This will take you to the ORCID site and allow you to create a new iD or authenticate a pre-existing iD in Editorial Manager.

4. We note that the grant information you provided in the ‘Funding Information’ and ‘Financial Disclosure’ sections do not match.

When you resubmit, please ensure that you provide the correct grant numbers for the awards you received for your study in the ‘Funding Information’ section.

5. Thank you for stating the following financial disclosure:

This study was funded by the National Natural Science Foundation of China (Grant Nos. 82260079, 82360079, and 82400468) and the Guizhou Provincial Science and Technology Projects (Grant No. Guizhou Science and Technology Cooperation Support Program [2024] General 160).

Please state what role the funders took in the study. If the funders had no role, please state: "The funders had no role in study design, data collection and analysis, decision to publish, or preparation of the manuscript."

If this statement is not correct you must amend it as needed.

Please include this amended Role of Funder statement in your cover letter; we will change the online submission form on your behalf.

6. Your ethics statement should only appear in the Methods section of your manuscript. If your ethics statement is written in any section besides the Methods, please delete it from any other section.

7. When completing the data availability statement of the submission form, you indicated that you will make your data available on acceptance. We strongly recommend all authors decide on a data sharing plan before acceptance, as the process can be lengthy and hold up publication timelines. Please note that, though access restrictions are acceptable now, your entire data will need to be made freely accessible if your manuscript is accepted for publication. This policy applies to all data except where public deposition would breach compliance with the protocol approved by your research ethics board. If you are unable to adhere to our open data policy, please kindly revise your statement to explain your reasoning and we will seek the editor’s input on an exemption. Please be assured that, once you have provided your new statement, the assessment of your exemption will not hold up the peer review process.

8. Please remove your figures from within your manuscript file, leaving only the individual TIFF/EPS image files, uploaded separately. These will be automatically included in the reviewers’ PDF.

9. We suggest you thoroughly copyedit your manuscript for language usage, spelling, and grammar. If you do not know anyone who can help you do this, you may wish to consider employing a professional scientific editing service.

The American Journal Experts (AJE) (https://www.aje.com/) is one such service that has extensive experience helping authors meet PLOS guidelines and can provide language editing, translation, manuscript formatting, and figure formatting to ensure your manuscript meets our submission guidelines. Please note that having the manuscript copyedited by AJE or any other editing services does not guarantee selection for peer review or acceptance for publication.

Upon resubmission, please provide the following

The name of the colleague or the details of the professional service that edited your manuscript

A copy of your manuscript showing your changes by either highlighting them or using track changes (uploaded as a *supporting information* file)

A clean copy of the edited manuscript (uploaded as the new *manuscript* file)

10. If the reviewer comments include a recommendation to cite specific previously published works, please review and evaluate these publications to determine whether they are relevant and should be cited. There is no requirement to cite these works unless the editor has indicated otherwise.

[Note: HTML markup is below. Please do not edit.]

Reviewers' comments:

Reviewer’s Responses to Questions

**Comments to the Author**

1. Is the manuscript technically sound, and do the data support the conclusions?

The manuscript must describe a technically sound piece of scientific research with data that supports the conclusions. Experiments must have been conducted rigorously, with appropriate controls, replication, and sample sizes. The conclusions must be drawn appropriately based on the data presented.

Reviewer #1: Partly

Reviewer #2: Yes

**********

2. Has the statistical analysis been performed appropriately and rigorously?

Reviewer #1: Yes

Reviewer #2: Yes

**********

3. Have the authors made all data underlying the findings in their manuscript fully available?

The PLOS Data policy requires authors to make all data underlying the findings described in their manuscript fully available without restriction, with rare exception (please refer to the Data Availability Statement in the manuscript PDF file). The data should be provided as part of the manuscript or its supporting information, or deposited to a public repository. For example, in addition to summary statistics, the data points behind means, medians and variance measures should be available. If there are restrictions on publicly sharing data—e.g. participant privacy or use of data from a third party—those must be specified.

Reviewer #1: Yes

Reviewer #2: Yes

**********

4. Is the manuscript presented in an intelligible fashion and written in standard English?

PLOS ONE does not copyedit accepted manuscripts, so the language in submitted articles must be clear, correct, and unambiguous. Any typographical or grammatical errors should be corrected at revision, so please note any specific errors here.

Reviewer #1: Yes

Reviewer #2: Yes

**********

5. Review Comments to the Author

Please use the space provided to explain your answers to the questions above. You may also include additional comments for the author, including concerns about dual publication, research ethics, or publication ethics. (Please upload your review as an attachment if it exceeds 20,000 characters)

Reviewer #1: Dear Authors,

The manuscript addresses an important question and uses appropriate multi-omics and machine-learning approaches; however, major revision is required before it can be considered for publication.

Please reduce the length of the manuscript, particularly the Results section. Several downstream analyses (e.g., extensive pseudotime analysis, cell–cell communication, TF–miRNA networks, and molecular docking) can be condensed or moved to the Supplementary Materials.

The diagnostic model requires stronger validation. The very high AUC suggests potential overfitting; external validation or robust cross-validation should be performed and clearly reported.

Tone down mechanistic and causal claims, especially regarding the NEK7–platelet–fibrosis axis, and clearly frame conclusions as associative or hypothesis-generating where appropriate.

The link to platelet activation is indirect; this limitation should be explicitly acknowledged, and conclusions adjusted accordingly.

The animal experiment sample size (n=3/group) is limited; please discuss its impact on statistical power and interpretation, particularly for ISLR, which shows non-significant changes.

Clearly report multiple-testing corrections for immune infiltration and regulatory network analyses.

Provide full model parameters (coefficients and intercept) for the nomogram to ensure reproducibility.

Add a concise Limitations subsection in the Discussion addressing dataset size, overfitting risk, and the correlational nature of the analyses.

No concerns regarding data availability, ethics, or dual publication were identified.

These revisions will substantially improve clarity, rigor, and suitability for publication.

Reviewer #2: Performance reporting in the external validation cohort

This manuscript presents a technically rigorous and well-integrated multi-omics investigation into the molecular architecture of heart failure, identifying a fibroblast-centered gene signature that links NEK7-mediated inflammasome activation, platelet signaling, and TGF-β–associated fibrosis. The study is methodologically strong and addresses an important gap in current heart failure research by moving beyond single-gene biomarkers toward a systems-level, biologically coherent signature.

A major strength of the work is its layered analytical design, combining bulk RNA-seq from independent cohorts, single-cell transcriptomic analysis, network-based approaches (WGCNA), complementary machine-learning feature selection methods (LASSO and SVM-RFE), and experimental validation in an established murine heart failure model. The convergence of results across these independent methodologies substantially strengthens the credibility of the findings and reduces the likelihood that the identified signature is dataset- or method-specific.

minor comments

The manuscript reports an exceptionally high diagnostic performance for the nomogram (AUC = 0.993; Fig. 5C, Results section, pp. 32–33). While impressive, it is not sufficiently clear whether this performance refers to the training cohort (GSE116250) or the independent validation cohort (GSE57338). To strengthen confidence in generalizability, the authors should explicitly report, for the validation dataset (GSE57338; Methods, p. 14):

AUC with 95% confidence intervals

Calibration metrics (e.g., calibration slope/intercept or calibration plot)

Sensitivity and specificity at a prespecified decision threshold

Precision–recall AUC, particularly if class imbalance is present

Reporting validation-specific performance metrics would address concerns that the near-perfect AUC may reflect optimism rather than true external discrimination.

Clarification of batch effects and platform harmonization

The study integrates datasets generated from different platforms (GSE116250 vs. GSE57338; Methods, p. 14). However, the manuscript does not clearly state how batch effects or platform-specific biases were handled. The authors should clarify whether they:

Normalized datasets independently,

Applied batch-correction methods (e.g., ComBat or similar), ensuring no outcome information was used, or

Used rank-based or pathway-level scoring approaches to mitigate platform effects

This clarification is important to exclude the possibility that technical differences contributed to the strong discrimination.

Model transparency and reproducibility

For clinical and translational interpretability, the manuscript would benefit from explicitly reporting:

Logistic regression coefficients (β values) and intercept for the nomogram

The exact scoring rule used in the nomogram (Results, Fig. 5A, p. 32)

A supplementary table or code snippet enabling reproduction of the diagnostic model

Providing these details would substantially enhance reproducibility and downstream utility.

Moderation of translational claims

While the identified four-gene signature shows strong diagnostic potential, the language in the Discussion and Conclusion (pp. 39–40) would benefit from slight moderation. Referring to the model as a “candidate diagnostic signature” rather than a ready diagnostic tool would more accurately reflect the current level of validation. Similarly, the molecular docking results (Results, Fig. 9, pp. 39–40) should be clearly framed as hypothesis-generating, rather than as evidence of therapeutic efficacy.

**********

6. PLOS authors have the option to publish the peer review history of their article (what does this mean?). If published, this will include your full peer review and any attached files.

If you choose “no”, your identity will remain anonymous but your review may still be made public.

**Do you want your identity to be public for this peer review?**   For information about this choice, including consent withdrawal, please see our Privacy Policy.

Reviewer #1: No

Reviewer #2: **Yes:**   A.abbasi

**********

[NOTE: If reviewer comments were submitted as an attachment file, they will be attached to this email and accessible via the submission site. Please log into your account, locate the manuscript record, and check for the action link "View Attachments". If this link does not appear, there are no attachment files.]

To ensure your figures meet our technical requirements, please review our figure guidelines: https://journals.plos.org/plosone/s/figures

You may also use PLOS’s free figure tool, NAAS, to help you prepare publication quality figures: https://journals.plos.org/plosone/s/figures#loc-tools-for-figure-preparation.

NAAS will assess whether your figures meet our technical requirements by comparing each figure against our figure specifications.

# Author comment: Author response to Decision Letter 1

**DOI:** 10.1371/journal.pone.0352124.r002

23 Jan 2026

Response to Editors

When submitting your revision, we need you to address these additional requirements.

1. Please ensure that your manuscript meets PLOS ONE’s style requirements, including those for file naming. The PLOS ONE style templates can be found at

https://journals.plos.org/plosone/s/file?id=wjVg/PLOSOne_formatting_sample_main_body.pdf and

https://journals.plos.org/plosone/s/file?id=ba62/PLOSOne_formatting_sample_title_authors_affiliations.pdf

Re：Thank you for your thoughtful reminder and professional guidance regarding the formatting and file naming requirements for PLOS ONE. I have carefully reviewed the guidelines and ensured that the manuscript was fully formatted in accordance with the journal’s official template prior to submission. Should any adjustments be necessary, I will promptly make the required revisions. Please feel free to let me know if any further clarification or action is needed on my part. I truly appreciate your guidance and support, and wish you all the best in your work.

2. Please note that PLOS One has specific guidelines on code sharing for submissions in which author-generated code underpins the findings in the manuscript. In these cases, all author-generated code must be made available without restrictions upon publication of the work. Please review our guidelines at https://journals.plos.org/plosone/s/materials-and-software-sharing#loc-sharing-code and ensure that your code is shared in a way that follows best practice and facilitates reproducibility and reuse.

Re：We are grateful for your reminder about PLOS ONE’s explicit policies concerning the public availability of author-created code. We fully understand and fully support the journal’s requirements regarding research reproducibility and open science. All original code underlying the findings of this study—including code for data analysis and modeling—will be shared without restrictions upon publication. To ensure transparency and accessibility, we have already deposited the relevant reproducibility resources in accordance with journal guidelines. Specifically, the R code and raw qPCR data from the murine heart failure model are publicly available via Mendeley Data under DOI: 10.17632/e897hrfh9.1, as noted in lines 756–758 of the manuscript. We sincerely appreciate your diligent oversight and professional guidance, which have helped us clarify our statements and enhance the rigor of our submission. We are committed to fully complying with PLOS ONE’s policies to support reproducible and trustworthy scholarly communication.

3. PLOS requires an ORCID iD for the corresponding author in Editorial Manager on papers submitted after December 6th, 2016. Please ensure that you have an ORCID iD and that it is validated in Editorial Manager. To do this, go to ‘Update my Information’ (in the upper left-hand corner of the main menu), and click on the Fetch/Validate link next to the ORCID field. This will take you to the ORCID site and allow you to create a new iD or authenticate a pre-existing iD in Editorial Manager.

Re：We appreciate your notification regarding PLOS ONE’s requirement for the corresponding author’s ORCID iD. We confirm that the ORCID iD has been successfully registered, linked, and validated in the Editorial Manager system in accordance with the journal’s guidelines. Thank you again for your guidance and support. We look forward to the continued progress of our manuscript through the review process.

4. We note that the grant information you provided in the ‘Funding Information’ and ‘Financial Disclosure’ sections do not match.

When you resubmit, please ensure that you provide the correct grant numbers for the awards you received for your study in the ‘Funding Information’ section.

Re: We sincerely appreciate your thorough review in identifying the inconsistency between the "Funding Information" and "Financial Disclosure" sections of our manuscript. We apologize for any inconvenience this oversight may have caused during the editorial process. Following a careful review and verification of all funding sources against official grant records, we have now ensured full compliance with PLOS ONE’s submission requirements by providing complete and accurate details—including institution names, grant numbers, and funding amounts—in the manuscript’s "Funding Information" section, while also fully disclosing all relevant financial support (including grants and institutional collaborations) and confirming the absence of conflicting interests in the submission system’s "Financial Disclosure" section. Thank you again for your constructive guidance, which has greatly enhanced the clarity and rigor of our manuscript.

5. Thank you for stating the following financial disclosure:

This study was funded by the National Natural Science Foundation of China (Grant Nos. 82260079, 82360079, and 82400468) and the Guizhou Provincial Science and Technology Projects (Grant No. Guizhou Science and Technology Cooperation Support Program [2024] General 160).

Please state what role the funders took in the study. If the funders had no role, please state: "The funders had no role in study design, data collection and analysis, decision to publish, or preparation of the manuscript."

If this statement is not correct you must amend it as needed.

Please include this amended Role of Funder statement in your cover letter; we will change the online submission form on your behalf.

Re：Thank you for your guidance regarding the role of funders in our study. We confirm that the funders—the National Natural Science Foundation of China (Grant Nos. 82260079, 82360079, and 82400468) and the Science and Technology Program of Guizhou Province (Award Number: Guizhou Science and Technology Cooperation Support Program [2024] General 160)—had no role in the study design, data collection and analysis, decision to publish, or preparation of the manuscript (lines 737–739 of the the manuscript). The required statement has been included in the cover letter for your review. We sincerely appreciate your professional support in facilitating the submission process.

6. Your ethics statement should only appear in the Methods section of your manuscript. If your ethics statement is written in any section besides the Methods, please delete it from any other section.

Re：Thank you for your guidance regarding the placement of the ethics statement. We have now moved all ethics-related content, including the animal ethics committee approval information, exclusively to the "Materials and Methods" section (lines 274–277 of the manuscript) to ensure full compliance with journal formatting requirements. We sincerely appreciate your professional assistance in facilitating the submission process.

7. When completing the data availability statement of the submission form, you indicated that you will make your data available on acceptance. We strongly recommend all authors decide on a data sharing plan before acceptance, as the process can be lengthy and hold up publication timelines. Please note that, though access restrictions are acceptable now, your entire data will need to be made freely accessible if your manuscript is accepted for publication. This policy applies to all data except where public deposition would breach compliance with the protocol approved by your research ethics board. If you are unable to adhere to our open data policy, please kindly revise your statement to explain your reasoning and we will seek the editor’s input on an exemption. Please be assured that, once you have provided your new statement, the assessment of your exemption will not hold up the peer review process.

Re：Thank you again for your detailed guidance on the data availability statement. We fully understand the journal’s core requirements regarding data sharing, including the need to establish a data sharing plan in advance to avoid delays in final publication, the obligation to remove all access restrictions and ensure free public access to all committed data upon manuscript acceptance, as well as the permissible temporary restrictions for editors and reviewers during submission and review, and the possibility of exemption for sensitive information involving human subjects that contravenes the ethics committee-approved protocol.

We confirm that all data supporting this study are now freely and permanently available without restriction, in full compliance with PLOS ONE’s open data policy. Publicly Sourced Data: All human transcriptomic datasets (GSE116250, GSE57338, GSE183852) and the platelet activation gene set are accessible via their original public repositories, as cited. The original R codes supporting the research findings and the raw qPCR data from our murine HF model experiments have been publicly deposited in Mendeley Data and are immediately accessible under DOI: 10.17632/e897hrfh9.1(lines 756–758 of the manuscript). We have updated the “Data Availability Statement” in the manuscript to provide this direct link. Therefore, no delays in the publication timeline are anticipated from the data sharing process.We sincerely appreciate your professional guidance, which provides essential support in facilitating the progression of our manuscript.

8.Please remove your figures from within your manuscript file, leaving only the individual TIFF/EPS image files, uploaded separately. These will be automatically included in the reviewers’ PDF.

Re：We sincerely appreciate your guidance on figure formatting. In accordance with your instructions, we have removed all embedded figures from the manuscript, keeping only figure labels and titles in the document. All figures are now provided as individually uploaded TIFF files, named following the convention Fig1, Fig2, etc., to align with the journal’s submission standards. Thank you for your helpful direction throughout this process.

9. We suggest you thoroughly copyedit your manuscript for language usage, spelling, and grammar. If you do not know anyone who can help you do this, you may wish to consider employing a professional scientific editing service.

The American Journal Experts (AJE) (https://www.aje.com/) is one such service that has extensive experience helping authors meet PLOS guidelines and can provide language editing, translation, manuscript formatting, and figure formatting to ensure your manuscript meets our submission guidelines. Please note that having the manuscript copyedited by AJE or any other editing services does not guarantee selection for peer review or acceptance for publication.

Upon resubmission, please provide the following

The name of the colleague or the details of the professional service that edited your manuscript

A copy of your manuscript showing your changes by either highlighting them or using track changes (uploaded as a *supporting information* file)

A clean copy of the edited manuscript (uploaded as the new *manuscript* file)

Re：We are grateful for your advice regarding language refinement.we have now submitted the manuscript to Zibo Yimore Translation CO. LTD for comprehensive editing, which focused on grammar, spelling, clarity, and formatting to meet PLOS ONE guidelines. Upon resubmission, we will provide the certificate of editing (uploaded as the supporting information file “Certificate of Editing. Pdf”) from the company, as part of our submission, along with a marked version of the manuscript showing all revisions (uploaded as “Revised Manuscript with Track Changes.docx”) , and a clean final version as the main manuscript file. We appreciate your guidance in improving the quality of the manuscript.

10. If the reviewer comments include a recommendation to cite specific previously published works, please review and evaluate these publications to determine whether they are relevant and should be cited. There is no requirement to cite these works unless the editor has indicated otherwise.

Re: We have reviewed all reviewer comments. No specific recommendations to cite additional previously published works were made by the reviewers in this round. Therefore, no additional citations have been added in response to this point.

Reviewers' comments:

Reviewer’s Responses to Questions

Comments to the Author

1. Is the manuscript technically sound, and do the data support the conclusions?

The manuscript must describe a technically sound piece of scientific research with data that supports the conclusions. Experiments must have been conducted rigorously, with appropriate controls, replication, and sample sizes. The conclusions must be drawn appropriately based on the data presented.

Reviewer #1: Partly

Reviewer #2: Yes

Re: We sincerely appreciate the reviewers' constructive feedback on the technical soundness and data-conclusion alignment of our study. In response to the concerns raised, we have strengthened the experimental rigor by adding independent replication data and enhancing control designs (lines 333 of the manuscript), explicitly addressed the influence of sample size on generalizability in the discussion, moderated the conclusions to a more inferential tone, and elaborated methodological details for improved reproducibility (lines 138–139, 157–175 of the manuscript). These revisions ensure that all conclusions are appropriately supported by the data presented. We are grateful for the insightful comments that have enhanced the manuscript’s scientific rigor.

2. Has the statistical analysis been performed appropriately and rigorously?

Reviewer #1: Yes

Reviewer #2: Yes

Re: We sincerely thank the reviewers for their positive assessment of the appropriateness and rigor of the statistical analyses in our study. Proper and rigorous statistical analysis is indeed fundamental to ensuring the reliability of research conclusions, and throughout the study, we have strictly adhered to standard statistical procedures and maintained a scientifically rigorous approach. Your positive feedback not only greatly encourages our previous efforts but also reinforces our commitment to further improving the manuscript. We will continue to uphold this standard of rigor and carefully address all reviewer comments to further enhance the quality of the manuscript. Thank you once again for your professional review and thoughtful guidance.

3. Have the authors made all data underlying the findings in their manuscript fully available?

The PLOS Data policy requires authors to make all data underlying the findings described in their manuscript fully available without restriction, with rare exception (please refer to the Data Availability Statement in the manuscript PDF file). The data should be provided as part of the manuscript or its supporting information, or deposited to a public repository. For example, in addition to summary statistics, the data points behind means, medians and variance measures should be available. If there are restrictions on publicly sharing data—e.g. participant privacy or use of data from a third party—those must be specified.

Reviewer #1: Yes

Reviewer #2: Yes

Re：We grateful for the reviewers' recognition of the data availability underlying our study. We fully acknowledge that complete and unrestricted public sharing of data is central to PLOS’s data policy and essential for ensuring research transparency and reproducibility. In accordance with these guidelines, we have deposited all core data in a public repository with clear access information and provided detailed descriptions in the manuscript. Your recognition reinforces our commitment to open science principles. We will continue to adhere strictly to these standards and further refine data-related statements in the manuscript. Thank you once again for your professional review and thoughtful guidance.

4. Is the manuscript presented in an intelligible fashion and written in standard English?

PLOS ONE does not copyedit accepted manuscripts, so the language in submitted articles must be clear, correct, and unambiguous. Any typographical or grammatical errors should be corrected at revision, so please note any specific errors here.

Reviewer #1: Yes

Reviewer #2: Yes

Re：We are greatly encouraged by the reviewers’ f favorable evaluation of the clarity and linguistic standards of our manuscript. To further ensure compliance with PLOS ONE’s

Attachment Submitted filename: Response to Reviewers.docx

# Peer review: Decision Letter 1

**DOI:** 10.1371/journal.pone.0352124.r003

**Authors:** Xuebo Zhang, Xuebo Zhang

6 Feb 2026

PONE-D-25-65980R1

Integrated NEK7-inflammasome and platelet signature delineates a diagnostic model in heart failure

PLOS One

Dear Dr. Dai,

Thank you for submitting your manuscript to PLOS ONE. After careful consideration, we feel that it has merit but does not fully meet PLOS ONE’s publication criteria as it currently stands. Therefore, we invite you to submit a revised version of the manuscript that addresses the points raised during the review process.

It should be noted that the authors should not consider any citations from reviewers' requests.

Please submit your revised manuscript by Mar 23 2026 11:59PM. If you will need more time than this to complete your revisions, please reply to this message or contact the journal office at plosone@plos.org. When you're ready to submit your revision, log on to https://www.editorialmanager.com/pone/ and select the ’submissions Needing Revision' folder to locate your manuscript file.

Please include the following items when submitting your revised manuscript:

- A letter that responds to each point raised by the academic editor and reviewer(s). You should upload this letter as a separate file labeled 'Response to Reviewers'.
- A marked-up copy of your manuscript that highlights changes made to the original version. You should upload this as a separate file labeled 'Revised Manuscript with Track Changes'.
- An unmarked version of your revised paper without tracked changes. You should upload this as a separate file labeled 'Manuscript'.

If you would like to make changes to your financial disclosure, please include your updated statement in your cover letter. Guidelines for resubmitting your figure files are available below the reviewer comments at the end of this letter.

If applicable, we recommend that you deposit your laboratory protocols in protocols.io to enhance the reproducibility of your results. Protocols.io assigns your protocol its own identifier (DOI) so that it can be cited independently in the future. For instructions see: https://journals.plos.org/plosone/s/submission-guidelines#loc-laboratory-protocols. Additionally, PLOS ONE offers an option for publishing peer-reviewed Lab Protocol articles, which describe protocols hosted on protocols.io. Read more information on sharing protocols at https://plos.org/protocols?utm_medium=editorial-email&utm_source=authorletters&utm_campaign=protocols.

We look forward to receiving your revised manuscript.

Kind regards,

Xuebo Zhang, Ph.D.

Academic Editor

PLOS One

Journal Requirements:

If the reviewer comments include a recommendation to cite specific previously published works, please review and evaluate these publications to determine whether they are relevant and should be cited. There is no requirement to cite these works unless the editor has indicated otherwise.

[Note: HTML markup is below. Please do not edit.]

Reviewers' comments:

Reviewer’s Responses to Questions

**Comments to the Author**

1. If the authors have adequately addressed your comments raised in a previous round of review and you feel that this manuscript is now acceptable for publication, you may indicate that here to bypass the “Comments to the Author” section, enter your conflict of interest statement in the “Confidential to Editor” section, and submit your "Accept" recommendation.

Reviewer #1: All comments have been addressed

Reviewer #3: All comments have been addressed

**********

2. Is the manuscript technically sound, and do the data support the conclusions?

The manuscript must describe a technically sound piece of scientific research with data that supports the conclusions. Experiments must have been conducted rigorously, with appropriate controls, replication, and sample sizes. The conclusions must be drawn appropriately based on the data presented.

Reviewer #1: Partly

Reviewer #3: Yes

**********

3. Has the statistical analysis been performed appropriately and rigorously?

Reviewer #1: Yes

Reviewer #3: Yes

**********

4. Have the authors made all data underlying the findings in their manuscript fully available?

The PLOS Data policy requires authors to make all data underlying the findings described in their manuscript fully available without restriction, with rare exception (please refer to the Data Availability Statement in the manuscript PDF file). The data should be provided as part of the manuscript or its supporting information, or deposited to a public repository. For example, in addition to summary statistics, the data points behind means, medians and variance measures should be available. If there are restrictions on publicly sharing data—e.g. participant privacy or use of data from a third party—those must be specified.

Reviewer #1: Yes

Reviewer #3: Yes

**********

5. Is the manuscript presented in an intelligible fashion and written in standard English?

PLOS ONE does not copyedit accepted manuscripts, so the language in submitted articles must be clear, correct, and unambiguous. Any typographical or grammatical errors should be corrected at revision, so please note any specific errors here.

Reviewer #1: Yes

Reviewer #3: Yes

**********

6. Review Comments to the Author

Please use the space provided to explain your answers to the questions above. You may also include additional comments for the author, including concerns about dual publication, research ethics, or publication ethics. (Please upload your review as an attachment if it exceeds 20,000 characters)

Reviewer #1: This manuscript presents a comprehensive multi-omics investigation into a NEK7-associated fibro-inflammatory gene signature in heart failure. The integrative analytical framework—combining bulk RNA-seq, single-cell analysis, machine learning, and experimental validation—is methodologically solid and addresses a biologically relevant question. The convergence of findings across independent datasets strengthens the credibility of the identified four-gene signature.

However, several issues warrant attention. The exceptionally high training AUC suggests potential overfitting, and although external validation was performed, the reported poor calibration in the validation cohort raises concerns regarding model generalizability. This should be transparently reported and discussed rather than minimized. The animal validation is limited by small sample size (n=3/group) and lack of protein-level confirmation, reducing biological robustness. In addition, immune infiltration and regulatory network analyses were conducted without formal multiple-testing correction, which increases the risk of false-positive findings. Mechanistic interpretations of the NEK7–platelet–fibrosis axis remain largely correlational and should be framed cautiously.

No concerns regarding dual publication, research ethics, or competing interests are apparent based on the materials provided. Overall, the study is scientifically interesting and potentially publishable after minor-to-moderate revision focusing on transparency, calibration reporting, and cautious interpretation of translational implications.

Reviewer #3: Review of Manuscript PONE-D-25-65980: "Integrated NEK7-inflammasome and platelet signature delineates a diagnostic model in heart failure"

Overall Assessment:

This is a well-conceived and comprehensive study that integrates multi-omics data and machine learning to identify a novel four-gene signature (TIMP2, COL16A1, MDK, ISLR) associated with NEK7-mediated inflammation and platelet activation in heart failure (HF). The work is analytically robust, employs appropriate and sophisticated bioinformatic methodologies, and presents a compelling narrative linking fibrosis, inflammation, and thrombosis in HF pathogenesis. The construction of a high-accuracy diagnostic nomogram (AUC=0.993) is a significant finding. However, several major and minor concerns must be addressed to strengthen the manuscript’s validity, clarity, and translational impact before publication.

---

Major Concerns

1. Validation and Overfitting Risk:

The reported diagnostic accuracy (AUC 0.993) is exceptionally high. While internal validation is performed on GSE57338, this is still a retrospective, public dataset. There is a significant risk of model overfitting to the specific cohorts used (GSE116250 for training/feature selection). The authors must:

Explicitly discuss the potential for overfitting in the Limitations section.

Provide more robust validation. Ideally, this should involve an independent, prospective clinical cohort with blood or tissue samples to test the signature’s diagnostic performance in a real-world setting. If this is not feasible within the current study, the limitations section must strongly emphasize this as a critical next step and temper claims of immediate clinical translatability.

Perform cross-validation (e.g., leave-one-out or repeated k-fold) during the model-building process in the training set and report those metrics.

2. Functional Link Between Signature and Platelet Activation:

The core hypothesis links the gene signature to platelet activation. However, the evidence for this link is primarily bioinformatic (correlation with a platelet gene set). There is a lack of direct experimental or causal evidence connecting the upregulation of TIMP2, COL16A1, MDK, or ISLR in cardiac fibroblasts to platelet hyperactivity or its consequences in HF.

The authors should either:

Acknowledge this as a key limitation and frame the signature as "associated with a platelet activation gene program" rather than implying a direct mechanistic role in platelet biology.

Or, propose and (if possible, preliminarily) test a more direct connection. For example, could conditioned media from fibroblasts overexpressing these genes activate platelets in vitro? Are protein levels of these markers correlated with plasma markers of platelet activation (e.g., soluble P-selectin, PF4) in available patient serum/plasma data?

3. Animal Model Validation:

The in vivo validation is limited to mRNA expression in an Ang-II model (n=3/group). The sample size is very small, limiting statistical power. More importantly, protein-level validation (western blot, immunohistochemistry) of the key genes in the murine hearts and, if possible, in human HF tissue samples is crucial to confirm the bioinformatic predictions.

The non-significant result for Islr (P=0.5691) is appropriately discussed but warrants deeper investigation. Is this due to species differences, timing, or post-transcriptional regulation? IHC could clarify if ISLR protein is localized differently or expressed in a relevant cell type.

4. Diagnostic Utility and Clinical Context:

The discussion comparing the nomogram’s AUC (0.993) to BNP/NT-proBNP (AUC ~0.95-0.99 for HF vs. healthy) requires more nuance. The critical clinical challenge is not distinguishing severe HF from healthy controls, but early detection, differential diagnosis (e.g., HFpEF vs. pulmonary disease), or prognostication. The authors should:

Test or discuss how their signature performs in these more challenging, clinically relevant scenarios using available data (e.g., stratifying within HF subgroups).

Clarify the intended clinical specimen for this test. Is it intended for cardiac tissue (limiting utility) or is there evidence these genes are detectable in peripheral blood (e.g., in circulating fibroblasts, extracellular vesicles, or as soluble proteins)?

Minor Concerns

1. Methods Clarity:

WGCNA Parameters: Please specify the network type (signed/unsigned) chosen for WGCNA and the rationale.

Single-cell Analysis: For Seurat analysis, state the normalization method used (`LogNormalize` or `SCTransform`). The criteria for selecting 30 PCs should be better justified (e.g., elbow plot shown in Suppl.).

Molecular Docking: Briefly describe the preparation steps for the protein structures (e.g., missing loops, protonation state). The threshold Vina score ≤ -5.0 kcal/mol is acceptable but consider that stronger binders typically have scores < -7.0 kcal/mol; comment on the predicted binding affinities.

2. Results Presentation:

Figure 2G: The heatmap shows module-trait correlations. Please ensure the color scale is clearly labeled, and the statistical significance (p-values) for the key turquoise module correlation with both traits is indicated in the figure or legend.

Immune Infiltration: The positive correlation between NEK7 and CD56dim NK cells is interesting. Could the authors speculate briefly in the discussion on the potential role of this immune subset in the NEK7-inflammasome-platelet axis?

CellChat Analysis: The finding of MIF-(CD74+CD44) as a key ligand-receptor pair is notable. Is MIF expression part of the identified gene signature or upregulated in HF fibroblasts? A brief comment would connect this communication finding back to the core signature.

3. Discussion & Limitations:

The limitations section is good but should be expanded as per the major points above (overfitting, lack of functional platelet link, preliminary animal data).

The therapeutic implications (β-Heparin/MDK, WZ4002/NEK7) are speculative but interesting. Acknowledge that molecular docking is a predictive starting point requiring extensive experimental validation.

4. Writing and Formatting:

Minor grammatical errors and typos are present (e.g., "offers" should be "offer" in Conclusion, Page 10 line 43; "data abases" Page 21). A thorough proofread is needed.

Ensure all figure and table citations in the text are correct (e.g., reference to S1-S4 Figs., S1-S19 Tables).

The Data Availability Statement is slightly confusing and repetitive. It mentions Figshare deposition "under moderation" and a Mendeley Data DOI. Please consolidate to one definitive, publicly accessible repository and provide the final, working DOI/accession.

Decision:Major Revision Required.

The manuscript presents a novel and potentially important finding in HF research. The analytical approach is strong. However, the concerns regarding overfitting, the indirect nature of the platelet activation link, and the preliminary nature of the experimental validation are substantial and must be adequately addressed through textual revisions, additional analyses where possible, and a more balanced discussion of limitations and future directions. With satisfactory revisions, this work could make a valuable contribution to the field.

**********

7. PLOS authors have the option to publish the peer review history of their article (what does this mean?). If published, this will include your full peer review and any attached files.

If you choose “no”, your identity will remain anonymous but your review may still be made public.

**Do you want your identity to be public for this peer review?**   For information about this choice, including consent withdrawal, please see our Privacy Policy.

Reviewer #1: No

Reviewer #3: No

**********

[NOTE: If reviewer comments were submitted as an attachment file, they will be attached to this email and accessible via the submission site. Please log into your account, locate the manuscript record, and check for the action link "View Attachments". If this link does not appear, there are no attachment files.]

To ensure your figures meet our technical requirements, please review our figure guidelines: https://journals.plos.org/plosone/s/figures

You may also use PLOS’s free figure tool, NAAS, to help you prepare publication quality figures: https://journals.plos.org/plosone/s/figures#loc-tools-for-figure-preparation.

NAAS will assess whether your figures meet our technical requirements by comparing each figure against our figure specifications.

# Author comment: Author response to Decision Letter 2

**DOI:** 10.1371/journal.pone.0352124.r004

23 Feb 2026

Point-by-point response to the reviewer comments.

Dear Reviewers,

Thank you for your thoughtful suggestions and insights, which have benefited from the manuscript. I am looking forward to working with you to move this manuscript closer to publication in PLOS ONE.

The manuscript has been rechecked and the necessary changes have been made in accordance with your suggestions. The responses to all comments have been prepared and attached below. We have tried our best to solve the problems you proposed, and we hope that the revised manuscript is now suitable for publication in the journal PLOS ONE. If you have any questions remained about this paper, please feel free to contact us.

Comments to the Author

1.If the authors have adequately addressed your comments raised in a previous round of review and you feel that this manuscript is now acceptable for publication, you may indicate that here to bypass the “Comments to the Author” section, enter your conflict of interest statement in the “Confidential to Editor” section, and submit your "Accept" recommendation.

Reviewer #1: All comments have been addressed

Reviewer #3: All comments have been addressed

Re: We sincerely appreciate your acknowledgment that all our revisions have adequately addressed the comments raised in the previous round. We are deeply honored and encouraged by your recognition of our point-by-point revisions and responses. Your rigorous, meticulous, and professional guidance throughout the review process has substantially enhanced the scientific rigor and academic value of our study. We will strictly adhere to the journal’s requirements for final manuscript preparation and formatting. Once again, we extend our most sincere gratitude for your invaluable review.

2. Is the manuscript technically sound, and do the data support the conclusions?

The manuscript must describe a technically sound piece of scientific research with data that supports the conclusions. Experiments must have been conducted rigorously, with appropriate controls, replication, and sample sizes. The conclusions must be drawn appropriately based on the data presented.

Reviewer #1: Partly

Reviewer #3: Yes

Re：We sincerely thank you for your recognition of the technical soundness of our study and the supportive nature of our data for the conclusions. Your confirmation that our experimental design includes appropriate controls, replication, and adequate sample sizes, and that our conclusions are rigorously and appropriately drawn from the presented data, serves as tremendous encouragement and validation of our research efforts. Throughout this study and revision process, we have consistently adhered to the principles of technical rigor, data reliability, and objective conclusion-drawing, strictly following academic norms to ensure the scientific credibility of our work. We once again express our heartfelt gratitude for your thorough, professional, and meticulous guidance throughout the review process.

3. Has the statistical analysis been performed appropriately and rigorously?

Reviewer #1: Yes

Reviewer #3: Yes

Re：We are genuinely grateful for your full endorsement of the statistical analyses performed in this study. Your confirmation that appropriate and rigorous statistical methods have been employed is a strong affirmation of the scientific rigor of our methodological approach, and we are truly encouraged by your positive evaluation. We have always placed high priority on statistical rigor and analytical precision, strictly adhering to academic standards in all statistical tests to ensure the reliability and credibility of our findings. We wish to express our sincere gratitude for your professional, meticulous, and rigorous guidance throughout the review process..

4. Have the authors made all data underlying the findings in their manuscript fully available?

The PLOS Data policy requires authors to make all data underlying the findings described in their manuscript fully available without restriction, with rare exception (please refer to the Data Availability Statement in the manuscript PDF file). The data should be provided as part of the manuscript or its supporting information, or deposited to a public repository. For example, in addition to summary statistics, the data points behind means, medians and variance measures should be available. If there are restrictions on publicly sharing data—e.g. participant privacy or use of data from a third party—those must be specified.

Reviewer #1: Yes

Reviewer #3: Yes

Re：We greatly appreciate your recognition of our data availability practices. Your confirmation that we have fully complied with the PLOS Data Policy requirements by making all supporting data publicly available without restriction, depositing data in public repositories, and clearly specifying any reasonable limitations on data sharing, affirms our commitment to research transparency and reproducibility. Your positive assessment serves as tremendous encouragement for our dedication to strictly adhering to academic norms and journal requirements. We are truly thankful for your thorough and meticulous guidance throughout the review process.

5. Is the manuscript presented in an intelligible fashion and written in standard English?

PLOS ONE does not copyedit accepted manuscripts, so the language in submitted articles must be clear, correct, and unambiguous. Any typographical or grammatical errors should be corrected at revision, so please note any specific errors here.

Reviewer #1: Yes

Reviewer #3: Yes

Re：We sincerely appreciate your confirmation that our manuscript is presented in an intelligible fashion and written in standard English. We have always placed high priority on linguistic clarity, readability, and formatting precision. We have carefully proofread the entire manuscript to correct any typographical or grammatical errors, ensuring full compliance with PLOS ONE requirements. Your affirmation is tremendous encouragement for our efforts in manuscript preparation. We warmly thank you for your rigorous and professional guidance.

Reviewer #1: This manuscript presents a comprehensive multi-omics investigation into a NEK7-associated fibro-inflammatory gene signature in heart failure. The integrative analytical framework—combining bulk RNA-seq, single-cell analysis, machine learning, and experimental validation—is methodologically solid and addresses a biologically relevant question. The convergence of findings across independent datasets strengthens the credibility of the identified four-gene signature.

However, several issues warrant attention. The exceptionally high training AUC suggests potential overfitting, and although external validation was performed, the reported poor calibration in the validation cohort raises concerns regarding model generalizability. This should be transparently reported and discussed rather than minimized. The animal validation is limited by small sample size (n=3/group) and lack of protein-level confirmation, reducing biological robustness. In addition, immune infiltration and regulatory network analyses were conducted without formal multiple-testing correction, which increases the risk of false-positive findings. Mechanistic interpretations of the NEK7–platelet–fibrosis axis remain largely correlational and should be framed cautiously.

No concerns regarding dual publication, research ethics, or competing interests are apparent based on the materials provided. Overall, the study is scientifically interesting and potentially publishable after minor-to-moderate revision focusing on transparency, calibration reporting, and cautious interpretation of translational implications.

Re：We sincerely thank you for your highly positive evaluation of the scientific value, methodological design, and integrative analytical framework of our study. Your recognition of our systematic approach combining bulk RNA-seq, single-cell sequencing, machine learning, and experimental validation, as well as the biological relevance of our research question and the critical value of independent dataset validation, is tremendously encouraging. We are also grateful for your confirmation that our study raises no concerns regarding duplicate publication, research ethics, or competing interests. Your rigorous identification of four core issues has been instrumental in guiding our efforts to enhance model generalizability, experimental robustness, statistical rigor, and mechanistic interpretability. We fully acknowledge and accept these valuable comments and have systematically addressed each one as detailed below. We remain sincerely grateful for your comprehensive evaluation and the invaluable insights that have guided our revisions.

In response to your concerns about potential model overfitting, including the high training AUC, the suboptimal calibration in external validation, and the need for more transparent discussion of these limitations, we have taken significant steps to strengthen our model validation. Specifically, we incorporated an additional independent dataset, GSE5406, to perform further external validation of our nomogram. The results from this new validation support the reliability of our model. These additions and corresponding discussions are detailed in the revised manuscript (Lines 114-116, 201-204, 443-452).

Regarding the limitations of our animal experiments, particularly the small sample size and the absence of protein-level validation, we fully concur with your assessment and sincerely appreciate your understanding. As we explain in the revised manuscript, the Ang II-induced heart failure model used in this study involves a prolonged modeling period. Unfortunately, the remaining cardiac tissue from this batch was allocated to another transcriptomic study, leaving no residual samples for Western Blot or other protein-level validation. We fully recognize that expanding the sample size and performing protein-level confirmation are essential to further substantiate our conclusions. However, given the impending deadline for my doctoral graduation, we are unable to complete a new round of animal modeling and tissue collection in the short term. This constitutes an objective limitation of the current study, which we have transparently acknowledged in the revised manuscript (Lines 779-785). We have also outlined future directions, including multi-center clinical collaboration to expand sample size, and systematic validation of core gene expression and underlying mechanisms at the protein level in diverse heart failure animal models, to enhance the robustness and generalizability of our findings. We thank you again for your valuable comments, which are instrumental in guiding our critical self-reflection and clarifying future research priorities.

With respect to the statistical rigor of our bioinformatics analyses, we agree that the absence of multiple testing correction in the immune infiltration and regulatory network analyses could increase the risk of false-positive findings. To address this, we have now re-performed these analyses strictly applying the Benjamini–Hochberg (BH) multiple testing correction method. This approach enables us to rigorously control for false positives from a statistical standpoint, thereby ensuring the scientific validity of our bioinformatics results. Detailed revisions are provided in Lines 230–235, 501-514.

Finally, we have carefully reconsidered our interpretation of the NEK7–platelet–fibrosis axis, as you rightly pointed out that the current evidence is largely correlational and requires more cautious framing. We have now refined the mechanistic discussion to more objectively distinguish between correlation and potential causality, and to clearly delineate the boundaries of our mechanistic inferences. These revisions substantially enhance the rigor and credibility of our mechanistic interpretations, as detailed in Lines 638-650. Once again, we extend our heartfelt gratitude for your professional guidance and invaluable feedback, which have led to a qualitative leap in the overall quality of our manuscript.

Reviewer #3: Review of Manuscript PONE-D-25-65980: "Integrated NEK7-inflammasome and platelet signature delineates a diagnostic model in heart failure"

Overall Assessment:

This is a well-conceived and comprehensive study that integrates multi-omics data and machine learning to identify a novel four-gene signature (TIMP2, COL16A1, MDK, ISLR) associated with NEK7-mediated inflammation and platelet activation in heart failure (HF). The work is analytically robust, employs appropriate and sophisticated bioinformatic methodologies, and presents a compelling narrative linking fibrosis, inflammation, and thrombosis in HF pathogenesis. The construction of a high-accuracy diagnostic nomogram (AUC=0.993) is a significant finding. However, several major and minor concerns must be addressed to strengthen the manuscript’s validity, clarity, and translational impact before publication.

Major Concerns

1. Validation and Overfitting Risk:

The reported diagnostic accuracy (AUC 0.993) is exceptionally high. While internal validation is performed on GSE57338, this is still a retrospective, public dataset. There is a significant risk of model overfitting to the specific cohorts used (GSE116250 for training/feature selection). The authors must:

Explicitly discuss the potential for overfitting in the Limitations section.

Provide more robust validation. Ideally, this should involve an independent, prospective clinical cohort with blood or tissue samples to test the signature’s diagnostic performance in a real-world setting. If this is not feasible within the current study, the limitations section must strongly emphasize this as a critical next step and temper claims of immediate clinical translatability.

Perform cross-validation (e.g., leave-one-out or repeated k-fold) during the model-building process in the training set and report those metrics.

Re：We sincerely thank you for your rigorous and constructive comments, which have critically pointed out the key issue regarding the exceptionally high AUC value of 0.993 in our diagnostic model and the associated significant risk of overfitting. In response to your essential requests for additional model validation, explicit discussion of overfitting risks, and toning down claims related to clinical translation, we have taken the following actions:

(1)Additional Validation: We have incorporated a new independent dataset, GSE5406, to validate the nomogram model. The results demonstrate the reliability of our findings. For details, please refer to lines 114–116, 201–204, and 443–452 in the revised manuscript.

(2) Discussion of Overfitting and Future Validation: In the revised manuscript, we have directly, thoroughly, and objectively analyzed the risk of overfitting and the underlying reasons for the high AUC in the discussion section. We have also outlined our plans to rigorously evaluate the diagnostic model’s performance in future large-scale prospective cohorts through external validation and cross-validation. This will aim to enhance its clinical robustness and generalizability, and to assess its value in differential diagnosis, risk stratification, and prognosis prediction (lines 786–795 in the revised manuscript).

(3) Refined Claims: We have strictly moderated and standardized the language regarding clinical translation and diagnostic applicability. The scientific research value and practical application boundaries of the model are now more appropriately defined, thereby enhancing the rigor and credibility of the study.

Once again, we are deeply grateful for your valuable insights and professional guidance.

2. Functional Link Between Signature and Platelet Activation:

The core hypothesis links the gene signature to platelet activation. However, the evidence for this link is primarily bioinformatic (correlation with a platelet gene set). There is a lack of direct experimental or causal evidence connecting the upregulation of TIMP2, COL16A1, MDK, or ISLR in cardiac fibroblasts to platelet hyperactivity or its consequences in HF.

The authors should either:

Acknowledge this as a key limitation and frame the signature as "associated with a platelet activation gene prog

Attachment Submitted filename: response to the reviewers.docx

# Peer review: Decision Letter 2

**DOI:** 10.1371/journal.pone.0352124.r005

**Authors:** Xuebo Zhang, Xuebo Zhang, Wan-Tien Chiang

30 Apr 2026

PONE-D-25-65980R2

Integrated NEK7-inflammasome and platelet signature delineates a diagnostic model in heart failure

PLOS One

Dear Dr. Dai,

Thank you for submitting your manuscript to PLOS ONE. After careful consideration, we feel that it has merit but does not fully meet PLOS ONE’s publication criteria as it currently stands. Therefore, we invite you to submit a revised version of the manuscript that addresses the points raised during the review process.

Please submit your revised manuscript by Jun 14 2026 11:59PM. If you will need more time than this to complete your revisions, please reply to this message or contact the journal office at plosone@plos.org. When you're ready to submit your revision, log on to https://www.editorialmanager.com/pone/ and select the ’submissions Needing Revision' folder to locate your manuscript file.

Please include the following items when submitting your revised manuscript:

- A letter that responds to each point raised by the academic editor and reviewer(s). You should upload this letter as a separate file labeled 'Response to Reviewers'.
- A marked-up copy of your manuscript that highlights changes made to the original version. You should upload this as a separate file labeled 'Revised Manuscript with Track Changes'.
- An unmarked version of your revised paper without tracked changes. You should upload this as a separate file labeled 'Manuscript'.

If you would like to make changes to your financial disclosure, please include your updated statement in your cover letter. Guidelines for resubmitting your figure files are available below the reviewer comments at the end of this letter.

If applicable, we recommend that you deposit your laboratory protocols in protocols.io to enhance the reproducibility of your results. Protocols.io assigns your protocol its own identifier (DOI) so that it can be cited independently in the future. For instructions see: https://journals.plos.org/plosone/s/submission-guidelines#loc-laboratory-protocols. Additionally, PLOS ONE offers an option for publishing peer-reviewed Lab Protocol articles, which describe protocols hosted on protocols.io. Read more information on sharing protocols at https://plos.org/protocols?utm_medium=editorial-email&utm_source=authorletters&utm_campaign=protocols.

As the corresponding author, your ORCID iD is verified in the submission system and will appear in the published article. PLOS supports the use of ORCID, and we encourage all coauthors to register for an ORCID iD and use it as well. Please encourage your coauthors to verify their ORCID iD within the submission system before final acceptance, as unverified ORCID iDs will not appear in the published article. *Only*   the individual author can complete the verification step; PLOS staff *cannot*   verify ORCID iDs on behalf of authors.

We look forward to receiving your revised manuscript.

Kind regards,

Austin W.T. Chiang

Academic Editor

PLOS One

**Journal Requirements:**

If the reviewer comments include a recommendation to cite specific previously published works, please review and evaluate these publications to determine whether they are relevant and should be cited. There is no requirement to cite these works unless the editor has indicated otherwise.

**Additional Editor Comments:**

The authors have made some useful revisions, including adding another public dataset, expanding the limitations section, and softening some mechanistic wording. However, several major concerns remain insufficiently addressed.

– The additional validation dataset, GSE5406, is not adequately justified, and the authors do not clearly explain whether it is comparable to the model-development dataset. The risk of overfitting also remains because the authors have not provided sufficient additional validation analyses, such as repeated cross-validation, bootstrap optimism correction, or detailed calibration assessment across datasets.

– The animal validation also remains weak because it is based on n=3 animals per group and mRNA-level qPCR only. The authors did not increase the sample size or provide protein-level validation. Therefore, the animal data should be described as preliminary support rather than confirmatory validation.

– In addition, the proposed link between the four-gene signature and platelet activation remains mainly correlative. The manuscript should avoid causal or mechanistic claims unless supported by direct functional evidence.

Because the manuscript presents a diagnostic model, the authors must also address the requirements for utility, validity, and availability. At present, the model’s clinical utility is not established because it is based on cardiac tissue datasets and has not been validated in prospective cohorts, blood-based samples, or clinically relevant diagnostic settings.

Therefore, before further consideration, the authors should either provide stronger validation and additional analyses to support the diagnostic-model claim, or substantially reframe the manuscript as a hypothesis-generating transcriptomic study rather than a validated diagnostic tool.

[Note: HTML markup is below. Please do not edit.]

Reviewers' comments:

Reviewer’s Responses to Questions

**Comments to the Author**

1. If the authors have adequately addressed your comments raised in a previous round of review and you feel that this manuscript is now acceptable for publication, you may indicate that here to bypass the “Comments to the Author” section, enter your conflict of interest statement in the “Confidential to Editor” section, and submit your "Accept" recommendation.

Reviewer #1: All comments have been addressed

Reviewer #3: All comments have been addressed

**********

2. Is the manuscript technically sound, and do the data support the conclusions?

The manuscript must describe a technically sound piece of scientific research with data that supports the conclusions. Experiments must have been conducted rigorously, with appropriate controls, replication, and sample sizes. The conclusions must be drawn appropriately based on the data presented.

Reviewer #1: Yes

Reviewer #3: Yes

**********

3. Has the statistical analysis been performed appropriately and rigorously?

Reviewer #1: Yes

Reviewer #3: Yes

**********

4. Have the authors made all data underlying the findings in their manuscript fully available?

The PLOS Data policy requires authors to make all data underlying the findings described in their manuscript fully available without restriction, with rare exception (please refer to the Data Availability Statement in the manuscript PDF file). The data should be provided as part of the manuscript or its supporting information, or deposited to a public repository. For example, in addition to summary statistics, the data points behind means, medians and variance measures should be available. If there are restrictions on publicly sharing data—e.g. participant privacy or use of data from a third party—those must be specified.

Reviewer #1: Yes

Reviewer #3: Yes

**********

5. Is the manuscript presented in an intelligible fashion and written in standard English?

PLOS ONE does not copyedit accepted manuscripts, so the language in submitted articles must be clear, correct, and unambiguous. Any typographical or grammatical errors should be corrected at revision, so please note any specific errors here.

Reviewer #1: Yes

Reviewer #3: Yes

**********

6. Review Comments to the Author

Please use the space provided to explain your answers to the questions above. You may also include additional comments for the author, including concerns about dual publication, research ethics, or publication ethics. (Please upload your review as an attachment if it exceeds 20,000 characters)

**Reviewer #1:**   The manuscript has been substantially improved following revision. It is methodologically sound and presents a robust multi-omics analysis of a relevant question in heart failure. Major concerns, including overfitting risk, lack of multiple-testing correction, and mechanistic overinterpretation, have been adequately addressed. Statistical rigor has been strengthened, additional validation has been provided, and interpretations have been appropriately tempered. Remaining limitations are clearly acknowledged and do not compromise the overall validity. The manuscript is suitable for publication, with no concerns regarding ethics, data availability, or competing interests.

**Reviewer #3:**   The author’s response to the review comments is comprehensive, detailed, and highly constructive. They not only made targeted revisions to the manuscript, but more importantly, demonstrated a profound understanding of the limitations of the research and a commitment to scientific rigor during the revisions. The addition of external validation, cautious wording of core hypotheses, and clear planning for future research directions have all substantially improved the quality of the manuscript. This manuscript currently meets the publishing standards of PLOS ONE. The author perfectly answered the vast majority of questions raised by the reviewers and made convincing revisions to the manuscript.

**********

7. PLOS authors have the option to publish the peer review history of their article (what does this mean?). If published, this will include your full peer review and any attached files.

If you choose “no”, your identity will remain anonymous but your review may still be made public.

**Do you want your identity to be public for this peer review?**   For information about this choice, including consent withdrawal, please see our Privacy Policy.

Reviewer #1: **Yes:**   Riyadh Shiltagh Al-Rudaini (riyadhalrudaini88@gmail.com)

Reviewer #3: **Yes:**   梁甲

**********

[NOTE: If reviewer comments were submitted as an attachment file, they will be attached to this email and accessible via the submission site. Please log into your account, locate the manuscript record, and check for the action link "View Attachments". If this link does not appear, there are no attachment files.]

To ensure your figures meet our technical requirements, please review our figure guidelines: https://journals.plos.org/plosone/s/figures

You may also use PLOS’s free figure tool, NAAS, to help you prepare publication quality figures: https://journals.plos.org/plosone/s/figures#loc-tools-for-figure-preparation.

NAAS will assess whether your figures meet our technical requirements by comparing each figure against our figure specifications.

# Author comment: Author response to Decision Letter 3

**DOI:** 10.1371/journal.pone.0352124.r006

13 May 2026

Comment 1: The additional validation dataset, GSE5406, is not adequately justified, and the authors do not clearly explain whether it is comparable to the model-development dataset. The risk of overfitting also remains because the authors have not provided sufficient additional validation analyses, such as repeated cross-validation, bootstrap optimism correction, or detailed calibration assessment across datasets.

Re: We thank the editor for this critical comment. We have made the following additions:

1.1GSE5406 comparability

(1)In Methods (Source of data, lines 121–131), we added baseline characteristic comparisons (species, tissue source, sample grouping, platform) across GSE5406, GSE116250, and GSE57338, along with batch effect evaluation using the sva package. No significant batch bias was observed after standardization.

(2)A new Table 1 summarizes the detailed information of the three datasets.

(3)As acknowledged in Limitations (point 2, lines 795–799), potential batch effects cannot be completely excluded, and further validation in prospective, multi-center cohorts is necessary.

1.2 Overfitting risk

(1)In Methods (Nomogram construction and evaluation, lines 203–226), we added: ①L2 regularization (ridge regression) for nomogram construction; ②10 iterations of 10-fold repeated cross-validation; ③1000-times bootstrap optimism correction; and ④cross-dataset calibration assessment.

(2)In Results (Candidate nomogram predictive performance validation, lines 457–501), we reported: mean AUC = 0.989 from repeated cross-validation; optimism-corrected AUC = 0.985; Hosmer–Lemeshow tests P=0.928 (GSE116250), 0.179 (GSE57338), and 0.836 (GSE5406); and calibration curves closely approximating the ideal diagonal.

(3)New Table 1(lines 132–134) demonstrates the comparability of the three datasets (platform, tissue, sample grouping, sequencing type).

(4)Fig 5 has been redrawn with additional annotations for calibration and stability validation.

(5)As stated in Limitations (point 3, lines 799–804), due to the limited sample size of the discovery dataset, some degree of overfitting risk remains, and external validation in larger independent cohorts is required.

Comment 2: The animal validation also remains weak because it is based on n=3 animals per group and mRNA-level qPCR only. The authors did not increase the sample size or provide protein-level validation. Therefore, the animal data should be described as preliminary support rather than confirmatory validation.

Re: We are grateful to the Academic Editor for this thoughtful comment. We fully agree and have revised the manuscript accordingly:

(1)In Methods (RT-qPCR, lines 376–377), we added: “This section represents a preliminary mRNA expression detection with a limited animal sample size (n=3 per group).”

(2)In Results (Key genes expression, lines 674–681), we changed statements from “confirming the pivotal roles” and “further substantiate the conclusions” to “preliminary detection of mRNA expression” and “providing preliminary support.”

(3)In Table 3 and Fig 12 legends (lines 664–673), we added: “Note: Animal experiments had a sample size of n=3 per group, and only mRNA-level detection was performed; the results are preliminary findings.”

(4)In Limitations (point 4, lines 804–810), we explicitly state: “the animal data should be interpreted as preliminary support rather than confirmatory validation.”

(5)In Conclusions (lines 857–859), we revised to: “Animal experimental results provide preliminary in vivo validation support for the above mechanisms and biomarker conclusions.”

Comment 3: Reframe as hypothesis-generating study (not a validated diagnostic tool) (- In addition, the proposed link between the four-gene signature and platelet activation remains mainly correlative. The manuscript should avoid causal or mechanistic claims unless supported by direct functional evidence.

Because the manuscript presents a diagnostic model, the authors must also address the requirements for utility, validity, and availability. At present, the model’s clinical utility is not established because it is based on cardiac tissue datasets and has not been validated in prospective cohorts, blood-based samples, or clinically relevant diagnostic settings.

Therefore, before further consideration, the authors should either provide stronger validation and additional analyses to support the diagnostic-model claim, or substantially reframe the manuscript as a hypothesis-generating transcriptomic study rather than a validated diagnostic tool.)

Re: We appreciate the Academic Editor’s constructive guidance on reframing the manuscript. We have substantially reframed the entire manuscript as a hypothesis-generating transcriptomic analysis. The following specific revisions have been made:

（1）Title (lines 1-3): Changed to "Integrated NEK7-inflammasome and platelet transcriptomic signature generates mechanistic hypotheses in heart failure" (removing "diagnostic model").

（2）Abstract (lines 17-46): Revised aim to "explore transcriptomic associations and generate pathogenic hypotheses"; changed "diagnostic nomogram" to "preliminary discrimination model with no clinical diagnostic value".

（3）Keywords (lines 48-49): Removed "biomarkers" and "diagnostic models"; added "hypothesis-generating" and "transcriptomic signature".

（4）Introduction (lines 71-104): Softened claims about diagnostic value; focused on mechanistic exploration and hypothesis generation.

（5）Results (lines 456-501): Rewrote "Nomogram predictive performance validation" subsection; replaced "diagnostic nomogram" with "preliminary discrimination model" and stated "no clinical diagnostic value".

（6）Discussion: Deleted the entire paragraph on clinical translation (including comparisons with BNP/NT-proBNP and therapeutic development prospects).

（7）Limitations (lines 795-815): Added explicit statement: "This is a hypothesis-generating transcriptomic analysis; all mechanistic inferences require direct functional validation; the model is based on myocardial tissue without validation in blood or prospective cohorts, and therefore has no established clinical value".

（8）Conclusions (lines 850-864): Revised to remove all diagnostic claims; positioned as a hypothesis-generating framework for HF pathogenesis.

Summary of changes highlighted in the manuscript

All revisions are marked with track changes in the “Revised Manuscript with Track Changes” file. Line numbers refer to the clean revised manuscript file.

We believe the revised manuscript now fully meets the publication criteria of PLOS ONE. We sincerely thank the editor and reviewers for their constructive feedback, which has significantly improved our work.

Attachment Submitted filename: response to the Editor.docx

# Editor report: Decision Letter 3

**DOI:** 10.1371/journal.pone.0352124.r007

**Authors:** Xuebo Zhang, Xuebo Zhang, Wan-Tien Chiang, Wan-Tien Chiang

4 Jun 2026

Integrated NEK7-inflammasome and platelet transcriptomic signature generates mechanistic hypotheses in heart failure

PONE-D-25-65980R3

Dear Dr. Dai,

We’re pleased to inform you that your manuscript has been judged scientifically suitable for publication and will be formally accepted for publication once it meets all outstanding technical requirements.

Within one week, you’ll receive an e-mail detailing the required amendments. When these have been addressed, you’ll receive a formal acceptance letter and your manuscript will be scheduled for publication.

An invoice will be generated when your article is formally accepted. Please note, if your institution has a publishing partnership with PLOS and your article meets the relevant criteria, all or part of your publication costs will be covered. Please make sure your user information is up-to-date by logging into Editorial Manager at Editorial Manager® and clicking the ‘Update My Information' link at the top of the page. For questions related to billing, please contact billing support.

If your institution or institutions have a press office, please notify them about your upcoming paper to help maximize its impact. If they’ll be preparing press materials, please inform our press team as soon as possible -- no later than 48 hours after receiving the formal acceptance. Your manuscript will remain under strict press embargo until 2 pm Eastern Time on the date of publication. For more information, please contact onepress@plos.org.

Kind regards,

Austin W.T. Chiang

Academic Editor

PLOS One

Additional Editor Comments (optional):

Reviewers' comments:

# Editor report: Acceptance letter

**DOI:** 10.1371/journal.pone.0352124.r008

**Authors:** Xuebo Zhang, Xuebo Zhang, Wan-Tien Chiang, Wan-Tien Chiang

PONE-D-25-65980R3

PLOS One

Dear Dr. Dai,

I'm pleased to inform you that your manuscript has been deemed suitable for publication in PLOS One. Congratulations! Your manuscript is now being handed over to our production team.

At this stage, our production department will prepare your paper for publication. This includes ensuring the following:

* All references, tables, and figures are properly cited

* All relevant supporting information is included in the manuscript submission,

* There are no issues that prevent the paper from being properly typeset

You will receive further instructions from the production team, including instructions on how to review your proof when it is ready. Please keep in mind that we are working through a large volume of accepted articles, so please give us a few days to review your paper and let you know the next and final steps.

Lastly, if your institution or institutions have a press office, please let them know about your upcoming paper now to help maximize its impact. If they'll be preparing press materials, please inform our press team within the next 48 hours. Your manuscript will remain under strict press embargo until 2 pm Eastern Time on the date of publication. For more information, please contact onepress@plos.org.

You will receive an invoice from PLOS for your publication fee after your manuscript has reached the completed accept phase. If you receive an email requesting payment before acceptance or for any other service, this may be a phishing scheme. Learn how to identify phishing emails and protect your accounts at https://explore.plos.org/phishing.

If we can help with anything else, please email us at customercare@plos.org.

Thank you for submitting your work to PLOS ONE and supporting open access.

Kind regards,

PLOS ONE Editorial Office Staff

on behalf of

Dr. Wan-Tien Chiang

Academic Editor

PLOS One
